# Robust Target Gene Discovery through Transcriptome Perturbations and Genome-Wide Enhancer Predictions in *Drosophila* Uncovers a Regulatory Basis for Sensory Specification

**DOI:** 10.1371/journal.pbio.1000435

**Published:** 2010-07-27

**Authors:** Stein Aerts, Xiao-Jiang Quan, Annelies Claeys, Marina Naval Sanchez, Phillip Tate, Jiekun Yan, Bassem A. Hassan

**Affiliations:** 1 Laboratory of Neurogenetics, Department of Molecular and Developmental Genetics, VIB, Leuven, Belgium; 2 Laboratory of Computational Biology, Katholieke Universiteit (K.U.) Leuven, Leuven, Belgium; 3 Center for Human Genetics, K.U. Leuven, Leuven, Belgium; 4 Doctoral Program in Molecular and Developmental Genetics, K.U. Leuven Group Biomedicine, Leuven, Belgium; University of California, Berkeley, United States of America

## Abstract

A comprehensive systems-level understanding of developmental programs requires the mapping of the underlying gene regulatory networks. While significant progress has been made in mapping a few such networks, almost all gene regulatory networks underlying cell-fate specification remain unknown and their discovery is significantly hampered by the paucity of generalized, in vivo validated tools of target gene and functional enhancer discovery. We combined genetic transcriptome perturbations and comprehensive computational analyses to identify a large cohort of target genes of the proneural and tumor suppressor factor Atonal, which specifies the switch from undifferentiated pluripotent cells to R8 photoreceptor neurons during larval development. Extensive in vivo validations of the predicted targets for the proneural factor Atonal demonstrate a 50% success rate of bona fide targets. Furthermore we show that these enhancers are functionally conserved by cloning orthologous enhancers from *Drosophila ananassae* and *D. virilis* in *D. melanogaster*. Finally, to investigate *cis*-regulatory cross-talk between Ato and other retinal differentiation transcription factors (TFs), we performed motif analyses and independent target predictions for Eyeless, Senseless, Suppressor of Hairless, Rough, and Glass. Our analyses show that *cis*TargetX identifies the correct motif from a set of coexpressed genes and accurately predicts target genes of individual TFs. The validated set of novel Ato targets exhibit functional enrichment of signaling molecules and a subset is predicted to be coregulated by other TFs within the retinal gene regulatory network.

## Abstract 2

CisTarget X is a novel computational method that accurately predicts Atonal governed regulatory networks in the retina of the fruit fly.

## Author Summary

Tens of thousands of regulatory elements determine the spatiotemporal expression pattern of protein-coding genes in the metazoan genome. Each regulatory element, when bound by the appropriate transcription factors, can affect the temporal transcription of a nearby target gene in a particular cell type. Annotating the genome for regulatory elements, as well as determining the input transcription factors for each element, is a key challenge in genome biology. In this study, we introduce a computational method, *cis*TargetX, that predicts transcription factor binding motifs and their target genes through the integration of gene expression data and comparative genomics. We first validate this method in silico using public gene expression data and, then, apply *cis*TargetX to the developmental program governing photoreceptor neuron specification in the retina of *Drosophila melanogaster*. Particularly, we perturbed predicted key transcription factors during the initial steps of neurogenesis; measure gene expression by microarrays; identify motifs and predict target genes; validate the predictions in vivo using transgenic animals; and study several functional and evolutionary aspects of the validated regulatory elements for the proneural factor Atonal. Overall, we show that *cis*TargetX efficiently predicts genetic regulatory interactions and provides mechanistic insight into gene regulatory networks of postembryonic developmental systems.

## Introduction

The development of the structural and functional properties of cells is largely determined via differential extraction of information from the genome by transcription factors (TFs). The first detailed analyses of TF-controlled genetic programs have recently been performed in yeast [1],[2] and in early embryonic development of sea squirt [3], sea urchin [4], and fruitfly [5],[6]. These initial studies revealed an astonishing complexity of regulatory interactions, between TFs and their target genes in the genome. The expression of most genes is regulated by combinations rather than single TFs, and extensive cross-regulations exist amongst TFs, often through feed-forward and feedback loops. These characteristics make it necessary to represent the regulatory blueprint of a cell as a network, for which the emerging properties explain the complement of active genes in that cell. The mapping and characterization of these networks represents a major goal in developmental biology, as they will yield profound mechanistic insights into embryonic and postembryonic developmental programs. However, the elucidation of such networks remains a formidable task for the vast majority of biological processes in most organisms.

Two main approaches are being used for gene regulatory network (GRN) mapping. The first approach relies on chromatin immunoprecipitation (ChIP) with an antibody against a particular TF, followed by hybridization on a chip (ChIP-chip) or next-generation sequencing (ChIP-Seq) to identify the regions bound by the TF. In yeast, a first draft of the entire regulatory network has been described [2] by ChIP-chip for every TF. Importantly, ChIP-chip data alone were not specific enough and required additional computational predictions of conserved TF binding sites in the bound regions. In *Drosophila*, ChIP-chip has been successful in identifying target genes for a few TFs, such as dorsal, Mef2, twist, and biniou [6],[7]. Here also ChIP-chip data alone were not specific enough, but combinations with computational binding-site predictions and with gene expression data under normal and TF perturbation conditions identified a significant number of *bona fide* regulatory interactions. The limitations of this approach are the large amounts of material required for ChIP (hence so far only successful for yeast cultures and large embryo collections) and the need for high quality, “ChIP-grade” antibodies. Therefore, it is not possible today to perform ChIP-Seq for most TFs at most developmental stages in multicellular organisms. The second approach is based on genetic perturbations of a TF, followed by quantitative measurements of expression level changes of downstream genes. Either a selected candidate gene set is measured by quantitative reverse transcription PCR (qRT-PCR), or all genes are measured. In yeast, a complete functional network was uncovered by profiling transcriptional responses of individual deletions of all TFs [1]. In higher eukaryotes, perturbation of multiple TFs (e.g., morpholino knock-down) followed by qRT-PCR or nanostring [8], have lead to several networks by measuring quantitative changes in gene expression upon TF perturbation. Examples of this approach are the endomesoderm network in sea urchin [4]; the network underlying central nervous system compartmentalization in *Ciona intestinalis* [9] and the network underlying mouse T-cell specification [10]. These networks are more complete than the ChIP-based networks because they contain interactions (i.e., targets) for many TFs. However, the limitations of this approach are (1) they are only applied as transient perturbations in early embryo's or in cell culture; and (2) these networks are based on expression changes and usually do not contain *cis*-regulatory evidence. In summary, while significant progress has been made in decoding regulatory interactions in cell culture models and early embryonic patterning, in vivo description of GRNs required during development remains a significant challenge for developmental and regulatory biology.

In order to begin to tackle this challenge, we exploited the second approach, which does not require special molecular reagents, to predict target genes of TFs involved in a specific postembryonic process, namely specification of *D. melanogaster* adult sense organs, and then provide direct in vivo *cis*-regulatory evidence for these interactions. A genetic TF perturbation followed by sample dissection and a microarray experiment, which is in principle feasible for any cell type, yields sets of up- and downregulated genes as candidate target genes for that TF. Bioinformatics methods to discover over-represented motifs across such a set of coexpressed genes, such as Clover, oPOSSUM, PASTAA, or PSCAN, are limited to small sequence search spaces, such as proximal promoters, often (except oPOSSUM) work on single genomes, and do not incorporate motif clustering [11]–[14]. On the other hand, motif scanning approaches that incorporate motif clustering, such as Stubb, SWAN, or Cluster-Buster, do not take gene coexpression information and genomic background information (i.e., genes not differentially expressed by the TF perturbation) into account [15]–[17]. Recently, methods that combine both approaches, namely motif over-representation and motif cluster scoring, like PhylCRM/Lever and ModuleMiner have been successfully used on yeast and human [18]–[20].

We developed a method, called *cis*TargetX, and applied it to *Drosophila*. *cis*TargetX produces high-confidence target predictions that result from statistical correlations between coexpressed gene sets and genome-wide target prioritizations on the basis of rankings of conserved motif cluster predictions. Unlike existing methods, *cis*TargetX allows identifying both the motif and the optimal subset of direct targets of the perturbed TF, and to dissect a set of coexpressed genes into subsets of targets of different TFs. Furthermore, its computational efficiency allows online usage by expert and nonexpert users through a Web-based application.

The developmental system we use as a model is retinal differentiation in *Drosophila*. This system has served as a model for the analysis of postembryonic development and cell-fate specification, and extensive genetic studies have uncovered key TFs and signaling pathways that control this process [21]. During the initial steps of photoreceptor specification, competent neuroepithelial cells specified by eye determination TFs such as Eyeless/Pax6 (Ey) express the proneural TF Atonal (Ato), leading to the specification of individual R8 photoreceptor precursor cells with a determined sensory fate. This process initiates a cascade of signaling events that result in the specification of all retinal cells. However, the regulatory interactions underlying this signaling cascade are unknown. Moreover, the fly retina is used as a cancer model [22]. Hence our endeavor to identify the regulatory environs of Ato may yield insight into the regulatory mechanisms underlying tumor suppression [23].

In order to determine the space of Ato downstream genes, we first generate microarray data using gain-of-function (GOF) and loss-of-function (LOF) genetic perturbation resulting in 451 Ato downstream genes. *cis*TargetX analysis of this set results in the prediction of 74 direct target genes. We then perform extensive in vivo enhancer-reporter validations for 39 predicted Ato enhancers and confirm 20 enhancers as bona fide Ato targets. Next, we apply *cis*TargetX to microarray data sets obtained under different conditions and for other TF perturbations, and dissect sets of coexpressed genes into direct targets of the TFs Ey, Senseless (Sens), Suppressor of Hairless (Su(H)), Rough (Ro), and Glass (gl). Drawing edges between TFs and their targets results in a transcriptional network underlying early retinal differentiation and defines the gene regulatory environs of Ato-dependent retinal differentiation. These data provide evidence for a generalized approach for the prediction and in vivo validation of postembryonic cell-fate specification GRNs.

## Results

### Reliable Genome-Wide Prediction of Transcriptional Targets Using Conserved Binding-Site Clusters and Gene Expression Data

**Figure 1:**
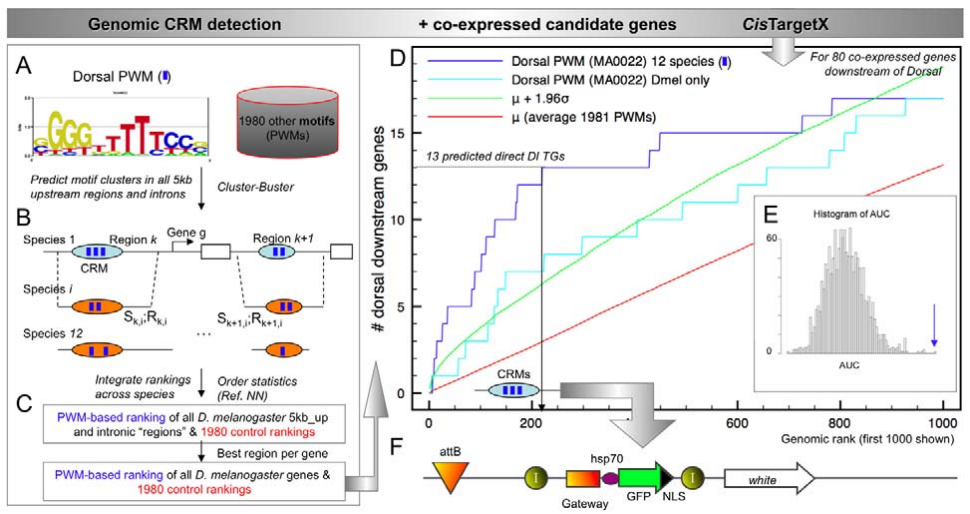
*cis*TargetX predictions of Dorsal target genes. (A) Example of scoring for homotypic clusters of binding sites with Cluster-Buster, using the Dorsal PWM and 1,980 other PWMs. (B) The scoring is applied to all 5-kb upstream sequences and introns (*k* = 1 to *k* = 93,330) of 13,667 genes across 12 *Drosophila* species (*i* = 1 to *i* = 12). (C) For each region *k* of species *i*, the highest score is retained and used to rank all regions in each species independently. The ranks *R*_k,i_ are integrated across the species into one ranking *R*_k_. The highest ranking region for each gene is retained to yield a final ranking of all *D. melanogaster* genes. (D) Using a set of candidate coexpressed genes, a receiver operating characteristic (ROC) curve is drawn using the dl-PWM based ranking on the *x*-axis and the recovery of the candidate genes in the *y*-axis. In this example, a set of 80 genes expressed downstream of dl is used [26]. The blue curve (using 12 species) shows significant enrichment of direct Dl targets within the set of 80 candidates. The optimal cut-off at position 220 yields a subset of 13 direct target predictions. (E) Histogram of AUC for all 1,981 PWMs tested, with the best performing PWMs being Dl PWMs, illustrating the use of motif discovery using ROC curves. (F) Predicted target regions are cloned in an enhancer reporter vector.

We apply a methodology for target gene discovery that combines genome-wide motif cluster predictions with gene set enrichment analysis. The procedure consists of two steps, illustrated in Figure 1 and Text S1 for the *Drosophila* homologue of the nuclear factor-κB (NFκB) TF Dorsal (dl) as a positive control. The dl binding motif is available as a position weight matrix (PWM) (Figure 1A), and many of its direct target genes are known [24]–[26]. Cluster-Buster [17] is used to predict clusters of dl binding sites across the 12 *Drosophila* genomes (Figure 1B). 5 kb upstream regions and introns of all *D. melanogaster* genes are scored, as well as all their respective orthologous regions from the 11 other *Drosophila* species, as determined using *liftover* on the UCSC Genome Browser net alignments [27]. Each *Dmel* reference region *k* receives 12 Cluster-Buster scores (*S*_k,i_, for each species *i*) and 12 corresponding ranks (*R*_k,i_, the rank position out of 93,330 regions). For each region, the 12 independent species ranks are integrated into one final rank (*R*_k_) using order statistics [28],[29], followed by selecting the highest ranking region for each gene, ultimately producing a final ranking of all *Dmel* genes (Figure 1C) [28]. Next, the genomic ranks of a subset of genes are plotted in a cumulative recovery curve (Figure 1D). For the dl example we use 80 coexpressed genes downstream of dl obtained from GOF and LOF Dorsal perturbations [26]. The observed recovery curve for these 80 genes (blue curve in Figure 1D) indicates that they are enriched in the top part of the motif-based gene ranking. This enrichment is higher when predictions are integrated across 12 genomes than for *Dmel* alone (cyan curve in Figure 1D), and is statistically significant (*z* score is 5.61), as determined by comparing the area under the curve (AUC) to the AUCs under 1,980 control curves constructed for an entire motif library (Table S1). The recovery curve yields 13 predicted targets at the optimal cutoff, of which 12 are true dl targets [7]. An important additional feature of this approach is its use for the detection of enriched motifs in the regulatory sequences of predicted target genes. This use is because AUC calculations are performed for all 1,981 motifs, thus allowing motif discovery by selecting the motif(s) with the highest AUC. That motif will have the highest enrichment of coexpressed candidate genes among its top-scoring target predictions. For the 80 genes downstream of dl, the dl motif is identified as the best motif with the highest AUC (Figure 1E; Table S2), together with several variations of the NFκB motif. Other motifs with significant recovery curves are the motif for Tinman, a homeobox NK family TF, and an E-box motif possibly representing binding sites for the basic-Helix-Loop-Helix TFs Twist or Snail. In conclusion, the dl motif together with dl target genes can be identified through homotypic binding-site cluster predictions, even though dl binding sites are usually accompanied in the *cis*-regulatory module (CRM) by binding sites for Twist, Snail, or other TFs [7]. Interestingly, the *cis*TargetX performance using only the dl PWM is similar to the performance when [dl+twi] or [dl+twi+sna] heterotypic cluster predictions are used (Figure S1). Although some bona fide enhancers receive better rankings using multiple PWMs, increasing the specificity, other enhancers are filtered out (namely those where dl works alone or cooperates with other TFs), decreasing the sensitivity. This balance of positive and negative effects of heterotypic versus homotypic models results in comparable recovery curves (Figure S1). Note that the cooperative regulation of target genes can be discovered through first discovering target genes for a single TF and then discovering overrepresented motifs of other TFs within the same target gene space.

To further test the performance of our approach, we performed similar computational experiments for other TFs using various types of input gene sets, such as coexpressed gene clusters from microarray gene expression, in situ gene expression, or literature-curated gene expression data; coregulated genes from chromatin-binding experiments; and functionally related genes from Gene Ontology. We find significant recovery curves, and accordingly high-confidence predictions of target genes, for Mef2, Cf2, Pointed, Serpent, Biniou, Svb, Bcd, Kr, Cad, Hb, and several other TFs (Table 1 and the *cis*TargetX Web site). Interestingly, these analyses yield a number of novel target gene predictions for these TFs, which are publicly available as an online resource for the community. Because *cis*TargetX uses a larger sequence space and a larger motif collection than other motif discovery methods (e.g., PASTAA, Clover, or PSCAN), and because it employs motif clustering and cross-species comparisons, *cis*TargetX identifies the correct motif with higher significance, and in more gene sets than other methods (Text S2).

**Table 1:** Validation of *cis*TargetX on various coexpressed gene sets.

Experiment	*n* Input Genes	Top-scoring PWMs	*z* Score	*n* Target Genes	Example Target Genes
Mef2 LOF [55]	684	MA0052-Mef2 [Jaspar]	3.61	68	aop, Mi-2, sls, Mp20, nau, up, wupA, Mlp84B, Mef2
		M00012-I-CF2II_01	4.97	49	Prm, wupA, up, Mhc, Zeelin1, if
		M00152-V-SRF_01	2.93	28	cher, CG10724, Mhc, tsr, Msp-300, if
Serpent LOF [69]	353	GATAAGC [Elemento]	6.02	56	NdaeI, Tl, Gel, ds, crq, Idgf2, Hph, …
Ey GOF [45]	189	Ey PWM [45]	3.54	14	so, Optix, eya, toy, Fas2, tie, osp, mspo, ey
Biniou LOF + ChIP [70]	144	M00474-V-FOXO1_02 (biniou)	3.42	23	hth, Ptp99A, lola, lbl, pnt, dia, fas, Fas3, bun, otk, vri, inv, EcR
Pointed GOF (“PLE”) [54]	25	M00233-V-MEF2_04	4.40	9	mib2, sty, Dg, drongo, Grip, Ppn, aop,..
		M01103-I-TWI_Q6	3.99	6	sty, aop, Grip, nuf, Dg, CG10275
		M00935-V-NFAT_Q4_01 (pnt)	3.27	4	wgn, nuf, sty, Dg
Dorsal LOF + GOF [26]	80	M00043-I-DL_01	6.04	13	Ths, ed, sna, Doc3, rho, sim, twi, vnd, dpp, sog, Mef2, Ect4, Neu3
polII ChIP early embryo [71]	1325	CAGGTAG (Zelda)	8.99	244	Sdc, Ptr, sisA, ec, aop, Kr, sc, vnd, …
		Bcd	3.57	22	Btd, tll, bnk, RpS30, slps1, eve, …
		Hb	2.11	90	Kni, odd, eve, Kr, nub, run, gt, …
		dl	2.19	41	Sdc, ths, sna, m4, hkb, Doc3, rho, …
GO:0007350	112	Bcd	3.88	15	Nkd, oc, btd, tll, slp1, run, eve, Kr, …
		Hb	2.78	38	Ubx, eve, odd, sog, pum, hth, kni, …
		Kr	3.25	26	Abd-B, hth, odd, pum, kni, abd-A, …
		cad	3.08	44	Slp2, kni, h, gt, tll, pum, odd, btd, …
GO:0009950	26	dl	5.41	6	twi, sna, cact, Tl, dpp, Egfr

Coexpressed gene sets were extracted from published microarray or ChIP-chip experiments, or from the FlyBase Gene Ontology annotation. The complete *cis*TargetX results of these analyses can be found on the *cis*TargetX Web site (http://med.kuleuven.be/cme-mg/lng/cisTargetX).

From these validation experiments we conclude that if a candidate input gene set—usually a set of coexpressed genes—contains a critical number of direct targets for a certain TF, then this procedure can identify the optimal motif for this TF together with the optimal subset of predicted direct target genes. The enhancer predictions that underlie the *cis*TargetX scores are also useful for identifying the actual enhancer regulating each target gene, although this step is more difficult to validate in silico because of limited data availability and because of the possible presence of redundant enhancers [7]. Therefore, validating these predictions requires in vivo testing of the putative enhancers.

### Direct Atonal Target Predictions on Genetic Perturbation Microarray Data

To unravel the GRN underlying sensory cell-fate specification, we turned to the *Drosophila* retina as a model system. The acquisition of neural cell fate in the retina is under the control of the proneural tumor suppressor TF Ato. Loss of Ato results in the complete failure of retinal differentiation [35], and therefore Ato must occupy a key position in the regulatory hierarchy underlying retinal development. However, only four target genes are currently known for Ato, namely *sens*, *dap*, *Brd*, and *mir-7*, yielding a poor explanation of the regulatory network underlying the complex process of Ato-dependent neural fate specification [30]–[33]. We therefore first focused on expanding the regulatory interactions directly downstream of Ato. To this end, we overexpressed Ato in the eye imaginal disc using two Gal4 drivers, namely GAL4-7 and AtoGAL4, verified the downstream effects on known targets by qRT-PCR, and then measured gene expression changes by microarrays (Methods, Figure S2). This GOF experiment results in a set of 204 Ato downstream genes (Methods, Table S3), containing the positive controls *sens* and *dap*, and is furthermore enriched in relevant biological processes, such as nervous system development (*p* = 4.1×10^−9^), and cell-fate commitment (*p* = 1.6×10^−5^).

**Figure 2:**
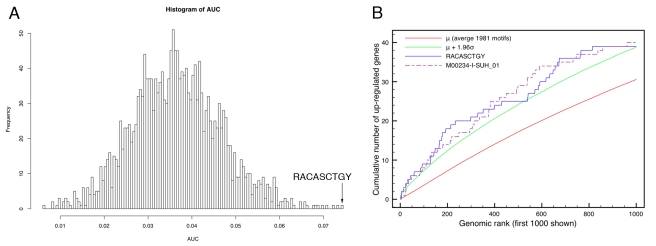
*cis*TargetX predictions of Ato target genes. (A) Histogram of AUC values for all ROC curves generated from the rankings using 1,981 PWMs and 204 upregulated genes under Ato GOF conditions. The six best PWMs are all E-box motifs. (B) ROC curve for the best performing motif, RACASCTGY (blue) and the seventh best motif (M00234 for Su(H)). The arrow indicates the optimal cut-off for RACASCTGat position 674, yielding 36 direct Ato target predictions.

Applying *cis*TargetX on this set of candidate genes identifies two kinds of motifs that produce highly significant recovery curves, namely E-box motifs and Su(H) motifs (Figure 2; Table S4). The best motif among the 1,981 motifs tested is the E-box motif RA**CA**SC**TG**Y from the Stark et al. conserved motif collection [34]. This motif is slightly different from the previously reported Ato binding-site consensus sequence AW**CA**KG**TG**K but preserves the typical CANNTG core [31]. We also constructed our own Ato “phylo-PWM” [28], on the basis of known Ato binding sites and conserved sites in other species (Table S5), which also yields a significant recovery curve (*z* = 2.67). The ROC curve for the RACASCTG motif is shown in Figure 2B. The AUC is significantly higher than expected by chance (*z* = 3.86) and applying the optimal cut-off at position 674 results in 36 direct Ato target-gene predictions (Tables 2 and S6). At this position on the *x*-axis, the observed recovery (*y*-axis; blue curve) of Ato upregulated genes, versus the expected recovery (*y*-axis; red curve), is most significant. We confirmed the specificity of the RACASCTGY motif for Ato by comparing *cis*TargetX results on Ato GOF data to control gene sets and to coexpressed genes enriched for specific targets of Scute, a related bHLH proneural factor. Scute downstream genes also have significant curves for several Su(H) and E-box motifs, but not for RACASCTGY. Using all significant E-boxes in the GOF Ato set (Table S4) yields, in total, 55 direct Ato target gene predictions (Tables 2 and S6).

**Table 2:** Predicted and validated enhancers.

CG	Symbol	BR	Motif (BR)	GOF/LOF	GFP	Location	dTSS	Size
CG3385	nvy	1	RRCAGGTGB-escargot	GOF	−	Intron 1	4 kb	1,000
CG11711	Mob1	3	M00693-V-E12_Q6	GOF and LOF	−	Upstream/Intron1	1–5 kb	1,289
CG31176	CG31176	3	sna	GOF and LOF	−	Intron	10 kb	937
**CG8965**	**CG8965**	**5**	**M00973-V-E2A_Q6**	**GOF and LOF**	**+**	**upstream**	**3 kb**	**619**
**CG31020**	**spdo**	**13**	**AACAGCTG**	**LOF**	**+**	**Upstream**	**1 kb**	**1,007**
CG2556	CG2556	15	RACASCTGY	GOF and LOF	−	Intron 1	2 kb	800
**CG3048**	**Traf1**	**16**	**RACASCTGY**	**LOF**	**+**	**Last Intron**	**3–12 kb**	**1,699**
CG13968	sNPF	16	RRCAGGTGB-escargot	GOF	−	Intron 1	3 kb	1,400
CG6741	a	16	atopwm3	GOF and LOF	−	Intron 3	25 kb	645
**CG17800**	**Dscam**	**18**	**MA0091**	**GOF**	**+**	**Intron 2**	**10 kb**	**1,100**
**CG7892**	**nmo**	**27**	**RACASCTGY**	**LOF**	**+**	**Intron 2**	**50 kb**	**753**
CG8118	mam	28	RACASCTGY	LOF	−	Upstream/Intron	6 kb/14 kb	650
CG7524	Src64B	34	atopwm3	GOF and LOF	−	Intron	12 kb	1,495
CG12806	Teh1	38	M00002-V-E47_01	GOF	−	Intron	6 kb	819
CG1794	Mmp2	38	RACASCTGY	LOF	−	Intron	50 kb	1,087
**CG30492**	**CG30492**	**40**	**M00693-V-E12_Q6**	**GOF**	**+**	**Upstream**	**0 kb**	**600**
CG6464	salm	40	RACASCTGY	LOF	−	Intron	5 kb	1,102
**CG17579**	**sca**	**41**	**M00002-V-E47_01**	**GOF and LOF**	**+**	**Intron 2**	**5 kb**	**1,500**
**CG7508**	**ato**	**47**	**atopwm3**	**GOF**	**+**	**Upstream**	**4 kb**	**300**
CG15138	beat-IIIc	61	RRCAGGTGB-escargot	GOF and LOF	−	Intron 1	8 kb	3,307
**CG11988**	**neur**	**62**	**M00712-V-MYOGENIN_Q6**	**GOF and LOF**	**+**	**Intron 1**	**8–14 kb**	**808**
**CG16757**	**Spn**	**65**	**atopwm3**	**GOF**	**+**	**Intron 1**	**1 kb**	**368**
CG10699	Lim3	96	RRCAGGTGB-escargot	GOF	−	Intron 2	10 kb	2,400
**CG10108**	**phyl**	**103**	**M00973-V-E2A_Q6**	**GOF and LOF**	**+**	**Intron 1**	**1 kb**	**1,125**
**CG33529**	**Rapgap1**	**104**	**atopwm3**	**GOF**	**+**	**Intron/upstream**	**1 kb**	**801**
**CG5411**	**Pde8**	**128**	**M00002-V-E47_01**	**GOF**	**+**	**Intron/upstream**	**0–9 kb**	**1,100**
**CG32434**	**siz**	**143**	**RACASCTGY**	**GOF**	**+**	**Intron**	**8–32 kb**	**1,894**
CG14622	DAAM	145	CAGCTGC	GOF and LOF	−	Intron/upstream	8–18 kb	918
CG9801	CG9801	155	M00973-V-E2A_Q6	GOF	−	Intron	5 kb	513
**CG32120**	**sens**	**164**	**RRCAGGTGB-escargot**	**GOF**	**+**	**Intron 2**	**2.5 kb**	**661**
**CG8365**	**E(spl)**	**167**	**RACASCTGY**	**GOF**	**+**	**Upstream**	**0.2 kb**	**1,103**
CG10076	spir	200	M00804-V-E2A_Q2	GOF	−	Intron 1	4 kb	1,659
CG8174	SRPK	208	MA0091	GOF	−	Intron/upstream	0.5 kb	649
CG6438	amon	209	M00712-V-MYOGENIN_Q6	GOF	−	Intron 1	5 kb	3,307
**CG6099**	**m4**	**212**	**RRCAGGTGB-escargot**	**GOF**	**+**	**Upstream**	**0.2 kb**	**279**
**CG3665**	**Fas2**	**282**	**MA0091**	**GOF**	**+**	**Intron 2**	**13 kb**	**539**
**CG1625**	**CG1625**	**323**	**M00001-V-MYOD_01**	**GOF**	**+**	**Upstream**	**0 kb**	**800**
CG6024	CG6024	618	RACASCTGY	GOF	−	Intron	5 kb	559
**CG1772**	**dap**	**648**	**RACASCTGY**	**GOF**	**+**	**Intron 2**	**2 kb**	**862**

List of *cis*TargetX-predicted Ato target enhancers. The third column, BR, is the best rank obtained in *cis*TargetX for that gene among the rankings of all significant E-box motifs. dTSS is the distance to the transcription start site. Location: an enhancer can be both upstream and intronic depending on alternative transcripts. GFP is “+” if the produced GFP colocalizes with and/or is expressed downstream of Ato, in at least one Ato-dependent tissue. Positive enhancers are shown in bold. Previously known Ato target enhancers were recovered (i.e., positive controls) for *ato*, *sens*, and *dap*. Previously known Scute target enhancers were identified for *siz*, *Traf4*, *m4*, and *E(spl)*.

Next we performed microarray experiments for *ato* ^−/−^ eye discs. Because loss of Atonal results in the complete loss of retinal differentiation [35], more genes are found that change expression, mostly downregulated genes. The most significant motif in a set of 315 downregulated genes (>3-fold downregulation) is Su(H), indicating that this TF is involved in many cell types throughout retinal differentiation. Not surprisingly, E-boxes are ranked lower than in the GOF analysis. RACASCTGY is nevertheless over-represented in the LOF set (*z* = 2.41), and yields 18 target gene predictions of which seven overlap with the GOF target predictions. Using all significant E-boxes (Table S7) and adding the GOF predictions yields a total of 74 predicted Ato target genes (Tables 2, 3, and S6). Analysis of Gene Ontology over-representation among these 74 genes yields biological processes that are not over-represented among the initial 204 Ato-upregulated genes, such as eye development (p = 1.1×10^−6^) and compound eye photoreceptor cell differentiation (*p* = 0.0041), indicating that the target predictions yield an enrichment towards the process under study.

**Table 3:** *cis*TargetX results for eye-developmental coexpressed gene sets.

Gene Set	*n* Input Genes	Data Source	Motif	Motif Rank	*z* Score	*n* Target Genes	Candidate TF
Ato GOF upregulated genes	204	This study	RACASCTGY (E-box)	1	3.86	36	Ato
			M00184-V-MYOD_Q6 (E-box)	2	3.74	22	Ato
			M00693-V-E12_Q6 (E-box)	3	3.63	18	Ato
			M00001-V-MYOD_01 (E-box)	4	3.37	24	Ato
			M00973-V-E2A_Q6 (E-box)	5	3.27	26	Ato
			RRCAGGTGB-escargot (E-box)	6	3.24	18	Ato
			M00234-I-SUH_01	7	3.18	34	Su(H)
			CGTGNGAA	8	3.06	9	Su(H)
			AtoPWM (E-box)	16	2.67	10	Ato
Ato LOF downregulated genes	317	This study	M00234-I-SUH_01	1	3.56	92	Su(H)
			Sens PWM	14	2.64	27	Sens
			M00712-V-MYOGENIN_Q6 (E-box)	16	2.55	46	Ato
			RACASCTGY (E-box)	25	2.40	18	Ato
Sens GOF downregulated genes	95	This study	M00148-V-SRY_01 (match sens core)	17	3.10	49	(Sens)
			Sens PWM (predicted from structure [48])		2.73	24	Sens
Sens GOF upregulated genes	77	This study	AATTAATT	7	3.94	4	Rough
			M00250-V-GFI1_01	28	2.82	12	Sens
			sens-RCWSWGATTTR [72]	29	2.81	7	Sens
Ey GOF (Ato-independent) upregulated genes	189	[45]	ey-PWM [45]	1	3.53	14	Ey
Eye-versus-wing eye-specific genes	723	[45]	CAATGCACTTCTGGGGCTTCCAC-glass [34]	11	2.48	22	Glass

For each gene set, one or more high-scoring motifs are in agreement with eye-developmental TFs and result in a subset of direct targets. Together, the motif-associated TF and its target genes allow mapping a retinal GRN (Figure S11).

### Validation of Predicted Ato Target Enhancers Through In Vivo Reporter Assays, Binding-Site Mutations, and Ectopic Activation

**Figure 3:**
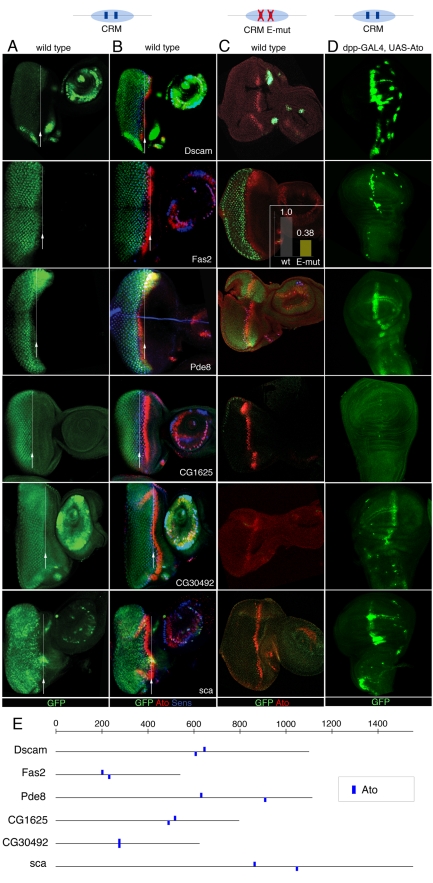
In vivo GFP reporter activities of predicted Ato target enhancers. Enhancer GFP-reporter assays for six positive enhancers. (A,B) Wild-type enhancer activity in wild-type eye-antennal discs showing GFP (A), and GFP plus Ato and Sens protein (B). The arrow and line indicate the initiation of GFP expression. GFP maturation causes a slight delay in GFP appearance posterior to Ato, as observed for positive controls (Figure S4). (C) Activity of the same enhancers with mutated Ato binding sites. (D) Response of the same wild-type enhancers to ectopic expression of Ato along the anterior-posterior boundary in wing imaginal discs of *dppGAL4-UASAto* animals. (E) Schematic of the enhancer sequences indicating the predicted Ato E-boxes in blue (see Methods). These E-boxes were mutated from CANNTG to CGNNCG.

To determine if any of the predicted genes are direct targets of Atonal, we tested 39 predictions by an in vivo enhancer reporter assay using a vector we designed for this purpose (Figure S3; Tables 3, S8, and S9). Of these, three were already known Ato targets, namely *dap*, *sens*, and *ato*, and four others are previously known Scute targets namely *siz*, *Traf4*, *m4*, and *E(spl)*. The enhancers of *dap*, *ato*, and *Traf4* were recloned in our vector, while for *sens*, *siz*, *m4*, and *E(spl)* we used the published lines [36],[37]. For the new enhancers, we selected genomic fragments that encompass high-scoring clusters of Ato binding sites, and we manually extended the fragments on both sides retaining flanking sequence with high *phastCons* [38] conservation scores across 12 *Drosophila* genomes, to prevent potentially fragmenting an enhancer. Most genes have multiple motif clusters, though here we selected only one per gene, usually the highest scoring region for our Ato PWM. Fragments ranging in size from 300 bp to 3,300 bp were cloned upstream of the *Hsp70* minimal promoter driving nuclear green fluorescent protein (GFP), and inserted into predefined genomic positions via ΦC31-mediated transgenesis [39]–[41]. The vector was tested using the previously known *ato* femoral chordotonal organ auto-regulatory enhancer [42] and the *dap* eye enhancer (Figure S3) [30]. In total, 20 enhancers produce reporter GFP expression in Ato-dependent photoreceptor precursor cells in the eye imaginal disc, or in the Ato-dependent chordotonal sensory organ precursors (SOPs), in wild-type animals (Figures 3 and S4; Table 2). These include the three previously known targets *sens*, *dap*, and *ato*; and 17 new Ato targets: *Fas2*, *CG30492*, *CG1626*, *Dscam*, *Pde8*, *sca*, *Rapgap1*, *Spn*, *CG8965*, *nmo*, *spdo*, *phyl*, *Traf4*, *m4*, *E(spl)*, *siz*, and *neur*. Thus, we achieved a 51% target-gene discovery success rate, even though we tested only one candidate region per gene. We note at least two caveats in these enhancer reporter assays. Namely, isolated fragments may lack necessary neighboring coactivating sites. Conversely, relatively short isolated fragments could lack neighboring repressive elements. To test whether this could have biased our findings, we compared the size of the positive and negative enhancers and found no significant difference in size (Figure S5), arguing against the under-representation of repressive elements in the positive versus negative enhancers. Furthermore, longer fragments (2 kb and 5 kb) flanking the *ato* autoregulatory enhancer do not cause loss of enhancer activity (Figure S5).

To investigate whether Ato is sufficient to activate the positive enhancers, we ectopically expressed Ato along the anterior-posterior axis of the wing disc using the *dppGal4* driver. 16 of the 20 tested enhancers show ectopic GFP expression along this boundary in response to Ato (Figures 3 and S6). To investigate whether the enhancers are dependent on the predicted Ato binding sites, we mutated predicted Ato binding sites in six positive enhancers from *Fas2*, *CG30492*, *CG1626*, *Dscam*, *Pde8*, and *sca*. All six enhancers showed altered expression upon the mutation of predicted Ato binding sites. For five of the six enhancers, GFP reporter expression is undetectable (*sca*, *CG30492*, and *Dscam*) or severely reduced (*CG1625*, *Pde8*) in the posterior part of the eye disc (Figure 3). The *Fas2* mutant enhancer does not show strong loss of GFP in the eye disc by immunofluorescence, however *GFP* mRNA levels produced by the mutated *Fas2* enhancers are 3-fold reduced compared to the wild-type enhancer (Figures 3 and 7). Furthermore, the mutant *Fas2* enhancer is no longer ectopically activated by Ato. These data demonstrate that the mutated Ato binding sites were predicted correctly for all target enhancers tested.

Examination of the molecular functions of the newly identified target genes, and the biological processes they are involved in, reveals that whereas several Ato target genes are known to be involved in neuronal specification and retinal differentiation (*nmo*, *Dscam*, *Fas2*, *sca*, *phyl*, *spdo*, *neur*, and *Traf4*), others we associate with these processes for the first time (*Pde8*, *Rapgap1*, and *Spn*), and for the unknown genes we provide a novel functional annotation (*CG1625*, *CG30492*, and *CG8965*).

### Ato Target Enhancers Are Functionally Conserved

**Figure 4:**
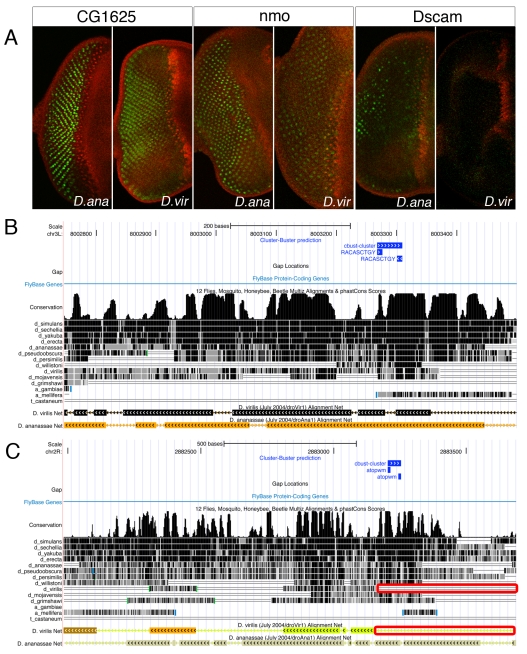
Validation of orthologous Ato target enhancers. (A) GFP reporter activity in the eye-antennal disc produced by *D. yakuba* and *D. virilis* orthologous sequences of the *nmo* and *Dscam* Ato target enhancers. Orthologous sequences were selected from the UCSC Genome Browser Multiz multiple alignments across 12 *Drosophila* genomes. (B) Screenshots from the UCSC Genome Browser showing the *nmo* enhancer with predicted E-boxes (blue track) and sequence constraint across the 12 *Drosophila* genomes as PhastCons scores (black track) and as aligned Nets, where the majority of the *D. melanogaster* sequence is conserved with *D. ananassae* and *D. virilis*, including the predicted E-box cluster. (C) Similar screenshot for the *Dscam* enhancer, showing a fragment of the *D. melanogaster* that is absent in the *D. virilis* orthologous sequence (red box), which contains the predicted E-box cluster.

Our target gene and enhancer predictions are based on high-scoring motif clusters across the 12 sequenced *Drosophila* species, hence the majority of the new Ato target enhancers are highly conserved in sequence. To test whether these enhancers are also functionally conserved, we tested the aligned sequences from two other species, namely D. *annannassae* and *D. virilis*, for three positive Ato target enhancers (*Dscam*, *CG1626*, and *nmo*) by reporter assays in *D. melanogaster*. We find that all enhancers that are conserved in sequence are also conserved in function, in terms of their activity downstream of Atonal in the eye disc (Figure 4). The Dscam enhancer is conserved in sequence between *D. melanogaster* and *D. ananassae*, but the *D. virilis* orthologous sequence lacks the large region where the *D. melanogaster* Ato binding sites are located (red box in Figure 4C). Expression analysis shows that the *D. ananassae* enhancer is active in the eye disc, whereas the *D. virilis* enhancer is not. Therefore, the newly identified regulatory regions are bona fide Ato target enhancers and Ato-dependent enhancer activity is under functional evolutionary constraint.

### Ato Target Enhancer Activity Is Not Restricted to the Retina

**Figure 5:**
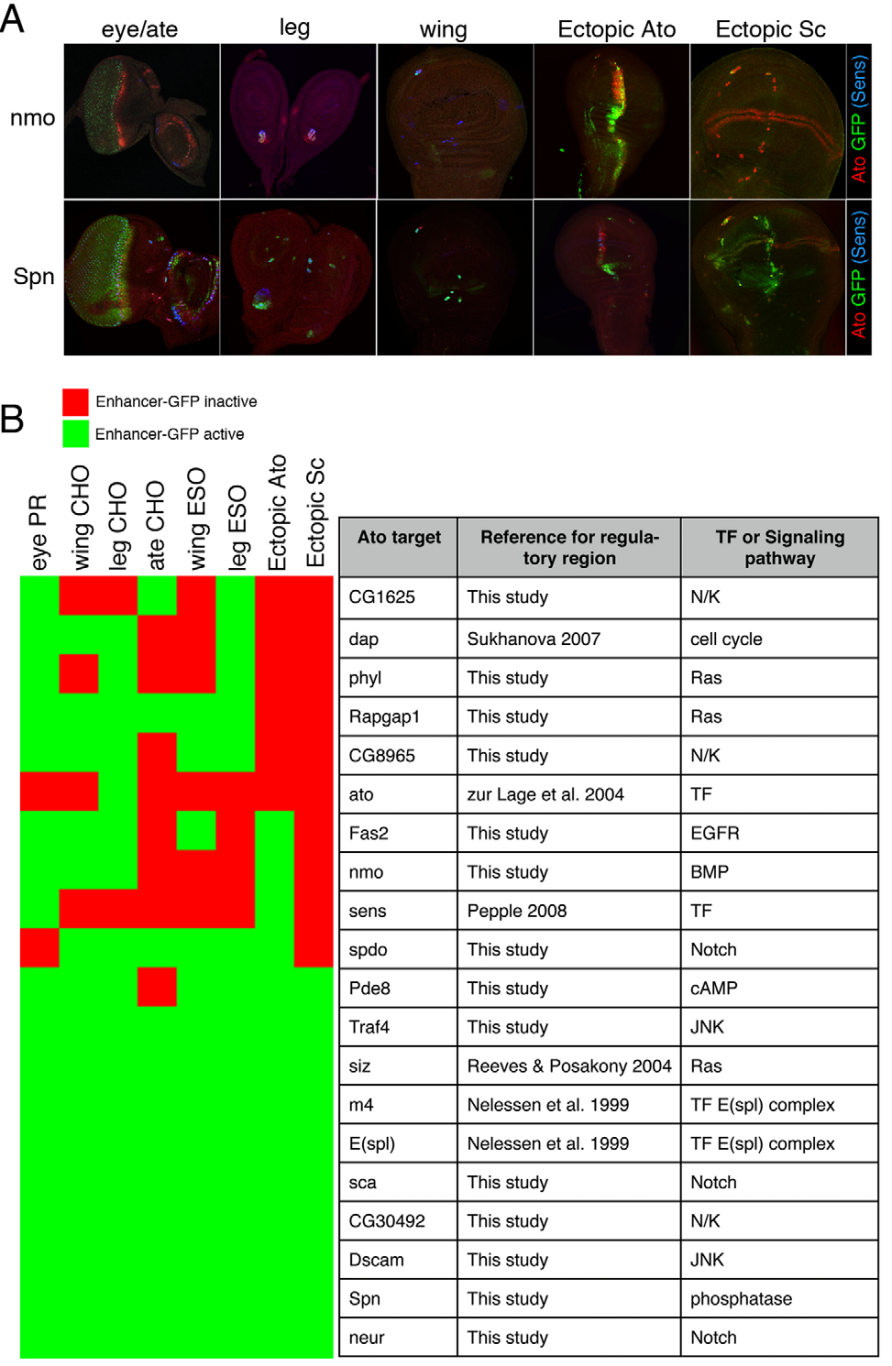
Ato target enhancer activity in other SOPs. (A) Reporter GFP activity for two examples of Ato target enhancers across different imaginal discs. Ato-overlapping activity is found in the photoreceptors, for *nmo* and *Spn*, in the antennal SOPs (*Spn*), in the leg chordotonal SOPs (*nmo* and *Spn*), in the wing chordotonal SOPs (*nmo* and *Spn*), and in other SOPs specified by another proneural factor Scute (*Spn*). Ato- and Scute-dependent activity is shown by ectopic expression of Atonal and Scute (*nmo* and *Spn*). (B) Unique combinations of signaling molecules are activated by Atonal in each sensory organ. The binary active/inactive summaries shown as green and red boxes are derived from GFP-reporter assays for all 20 Ato target enhancers (Figures S8 and S9). Atonal target genes analyzed are signaling molecules or TFs.

Ato not only specifies the visual sensory receptors, but also the hearing, balance, and stretch sensory organs [35],[43]. Our ignorance of the proneural code is highlighted by the fact that no known genes explain how a single proneural TF specifies different sense organs. We reasoned that the large set of Ato targets identified here could provide insight into how diverse specification programs are controlled by the same proneural factor. To this end, we examined the GFP expression patterns of the 20 Ato target enhancers across various imaginal discs under wild-type conditions, and in the wing imaginal disc under ectopic Ato expression conditions (Figures 5, S4, S8, and S9). We find that none of these Ato target enhancers is specific to a single sensory organ subtype. Instead, we observe extensive reuse of targets across multiple organs as depicted in a heatmap plotting enhancer activation per sensory organ subtype (Figure 5B). Particularly, we find that there exist two classes of enhancers. The first class, representing 45% of the targets (nine out of 20, all green in Figure 5), is active in all sense organs examined; is easily ectopically activated by Ato; and contains genes such as *Spn*, *Dscam* and *neur* (Figures S4, S8, and S9). These data suggest that these enhancers form the core of a universal postembryonic Ato-dependent sensory program. Although unlikely, it cannot be fully excluded that some of these enhancers may have more restricted Ato-dependent activity patterns, because fragments cloned in this work lack putative repressor information. The second class of enhancers is restricted to a subset of sense organs and most show weak or no response to ectopic stimulation. This class contains genes such as *Fas2* and *nmo* (Figures S4, S8, and S9). Interestingly, each enhancer of this class has a unique activity pattern (Figure 5B, rows in the heatmap). Combined, these two enhancer subtypes yield a unique combination of targets for each sensory organ developmental program (Figure 5B, columns in the heatmap). Because many proneural target genes are signaling molecules representing a diverse set of major developmental pathways such as BMP, Notch, Wnt, EGFR/Ras, JNK, and small GTPases, the differential transcriptional modulation of these signaling molecules between different sense organs could result in different developmental programs downstream of Atonal [44].

### Using *cis*TargetX for GRN Prediction

**Figure 6:**
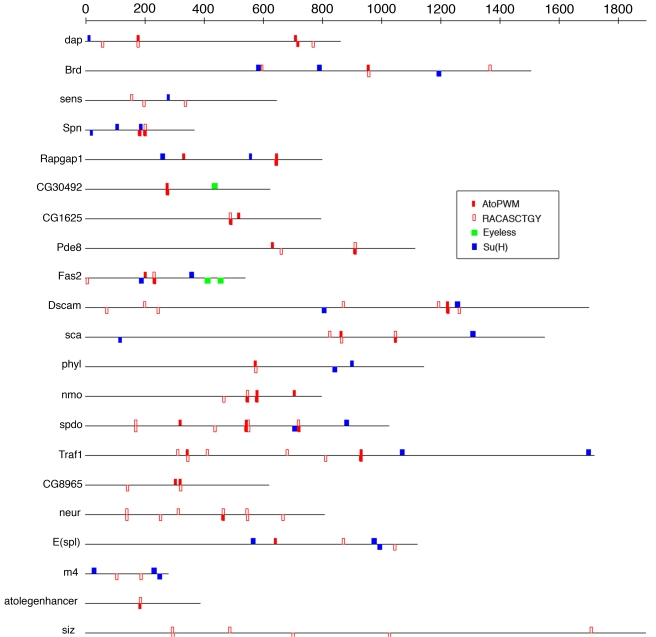
Motif analysis across Ato target enhancers. Motif over-representation analysis using the Clover algorithm finds E-boxes (red) and Su(H) motifs (blue) as highly over-represented (*p*<0.001) across the Ato target enhancers. The ey motif (green) is not statistically over-represented but was found by independent ey target discovery with *cis*TargetX (the Eyeless site predictions are generated by Cluster-Buster).

**Figure 7:**
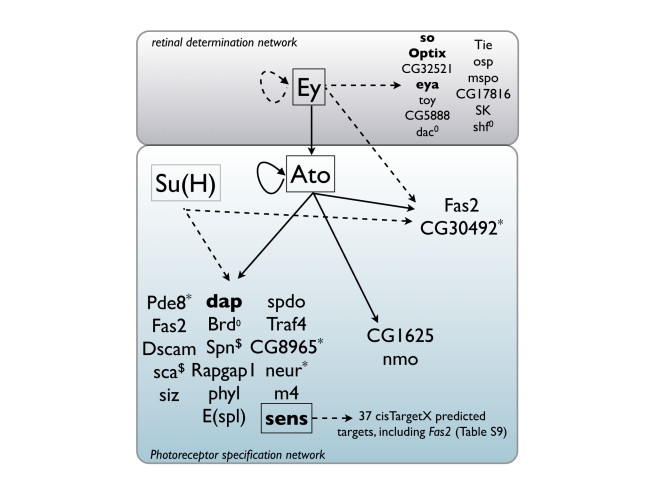
Target gene predictions for Atonal and associated retinal TFs. Predicted target genes for Ey and Su(H) and validated target genes for Ato, showing two coregulated targets of Ato and Ey (Fas2 and CG30492) and extensive coregulation between Ato and Su(H). Full arrows represent validated target genes, dashed arrows represent predicted target genes by *cis*TargetX. Genes in bold face are previously known target genes. ^0^, previously known target genes that are not detected in this study; ^*^, Su(H)-predicted target genes by *cis*TargetX that are also Ato targets yet without predicted binding sites in the Ato target enhancer; ^$^, genes not predicted as Su(H) by *cis*TargetX yet the Ato target enhancers contain predicted Su(H) binding sites. The full GRN can be found in Figure S11 and Table S10.

The GRN underlying photoreceptor differentiation is expected to comprise many TFs. Using previously published microarray data comparing wild-type eye imaginal discs with wild-type wing imaginal discs [45], together with our Ato LOF microarray data, we find at least 94 TFs either enriched in the eye disc compared to the wing disc or significantly downregulated in *ato* ^−/−^ eye discs respectively. Determining the regulatory interactions between all these TFs and their target genes, as well as among the TFs themselves, will be a considerable undertaking. To achieve this, either ChIP-grade antibodies are required for all these TFs together with ChIP procedures optimized for small sample sizes (e.g., only few thousands cells). Alternatively, once high-quality position weight matrices are available for these factors, for example thanks to protein-binding microarrays [46] or other approaches [47], we will be able to apply similar procedures as we applied for Ato above. Indeed, our validation experiments in Table 1 show that this may be feasible for other TFs. For example, to predict Ey targets we used publicly available microarray data obtained from wild-type and Ey-GOF imaginal discs. In a set of 189 upregulated genes after Ey overexpression (this was done in a normal and an *ato* ^−/−^ background to obtain Ato-independent Ey-downstream genes), *cis*TargetX identifies the Ey motif [45] as the best motif among the 1,981 tested motifs, with 14 predicted direct targets, including known or likely Ey targets like *so*, *Optix*, *eya*, *toy*, and *Tie* (Figure S10; Table 3). Two predicted Ey target genes, namely *Fas2* and *CG30492*, had also been identified above as Ato targets using independent (Ato GOF) data. Remarkably, the predicted genomic binding sites for Ey fall within the Ato target regions (Figure 6), implying potential combinatorial control of Ey and Ato on shared target CRMs. This result may explain why the mutation of Ato binding sites alone in the *Fas2* enhancer weakens but does not abolish its activity. We suggest that these factors cooperatively regulate a number of targets and therefore constitute a feed-forward regulatory loop with at least two shared target genes (Figure 7). Note that this combinatorial regulation could not be discovered by motif analysis on the validated Ato target enhancers (using Clover), nor by heterotypic *cis*TargetX analysis, because this code represents only a minority of the Ato targets discovered thus far, yet independent Ey target discovery identified the cooperativity simply by overlapping target sets.

As a final example of target discovery using TF perturbations, we performed an additional TF perturbation experiment followed by microarrays on three biological replicates for the Zinc-finger TF Senseless (eye-antennal imaginal discs from *atoGAL4 × UAS-Sens*). Among a set of 97 significantly (*p*<0.01) downregulated genes, *cis*TargetX identifies a Sens-related motif, namely a predicted motif using the Sens Zinc-fingers [48] as having a significantly (*z* = 2.73) enriched subset of 24 predicted targets among these 97 genes (Figure S10; Table S10), including a shared target with Ato, namely *Fas2*. Interestingly, *cis*TargetX also identifies Sens-related motifs in a set of upregulated genes (*p*<0.05 and at least 2-fold upregulation), namely the RCWSWGATTTR consensus and the GFI PWM from TRANSFAC (M00250). These analyses confirm that TF perturbations allow identifying subsets of direct target genes of the perturbed TF.

Although gene expression analyses, unlike ChIP for example, after TF perturbation are feasible for any TF, performing such experiments for the purpose of mapping an entire network would still represent an extensive effort. We therefore investigated whether direct target genes can be predicted from microarray data obtained under wild-type conditions. Ostrin et al. [45] determined gene expression profiles in wild-type eye imaginal discs and in wing imaginal discs, as controls for their Ey-overexpression studies. We used these control hybridizations to identify a set of 211 genes enriched in the eye disc (>1.5-fold) and used it as input for *cis*TargetX. Significant motifs found in this set include motifs of TFs with known eye functions, such as Su(H) (best motif, *z* = 3.20), Stat92E (*z = *2.85), Atonal (*z = *2.74), and glass (*z = *2.02) (Table 3). The Atonal predicted targets from this set overlap with the Ato GOF targets identified above (e.g., *neur*, *m4*, *CG8965*, *Traf1*, *Pde8*) but also include new predictions that are likely true targets based on their established role or expression pattern, such as *argos*. The Su(H) motif found in this set was also identified as an important motif in the set of Ato-upregulated genes. Several of the predicted Su(H) targets (see Table S10) are known or likely true Su(H) targets, such as *E(spl)*, *m4*, *HLHmgamma*, *phyl*, and *neur*. We moreover find a large overlap between predicted Su(H) targets and validated Ato targets (Figure 7), and for the majority of the shared targets, although not all, the predicted target region coincides with the Ato target region. This finding corroborates previous findings of cooperative regulation by Su(H) and a proneural factor [36],[37]. Additionally, a motif discovery analysis among the validated Ato target regions using Clover [14] identifies the Su(H) motif as significantly over-represented (*p*<0.001) (Figure 6; Table S11). Nevertheless, some predicted shared Su(H)-Ato target genes have no Su(H) binding sites within the Ato target regions (e.g., *Pde8*, *neur*, *CG30492*, and *CG8965*), and could be coregulated through different enhancers.

This experiment, using coexpressed gene sets from wild-type tissues, illustrates how a set of coexpressed genes can be dissected into target genes of different TFs that operate in the same network neighborhood. This finding may be important because similar approaches can be applied in evolutionary studies using organisms for which transgenesis, and hence TF perturbation, is not feasible.

Finally, using the significant target gene predictions for Ey, Atonal, Su(H), Sens, and Glass, and adding previously published regulatory interactions, we derive a putative GRN underlying retinal differentiation, containing 250 predicted regulatory interactions between 177 genes (Figures 7 and S11; Table S10). This predicted network highlights extensive combinatorial regulation downstream of Ey, and suggests that signal transduction molecules may be key targets of the transcriptional program of retinal differentiation as they are highly over-represented in the network (GO:0007165; *p* = 10^−10^ for all 177 genes of the network).

## Discussion

### A High Confidence Approach to Regulatory Network Prediction

In this study we apply an integrated genetics and computational pipeline to identify functional target genes and target enhancers of TFs in the GRN underlying sensory organ development in *Drosophila*. Identifying target genes for any TF through genome scanning remains a significant challenge because any given consensus sequence has 10^3^–10^6^ instances throughout the genome [49]. For example, there are more than 600,000 matches to the canonical E-box motif CANNTG in the genome and ∼10,000 to ∼200,000 single matches to the more specific Ato motif (Table S5), depending on the similarity threshold employed [50]. To solve this problem we developed a method called *cis*TargetX to predict motif clusters across the entire genomes of 12 *Drosophila* species and determine significant associations between motifs and subsets of coexpressed genes. Validation of *cis*TargetX on publicly available gene sets identifies the correct motif and targets for nearly all tested TFs, demonstrating the general utility of approach. We therefore developed a *cis*TargetX Web tool available freely at http://med.kuleuven.be/cme-mg/lng/cisTargetX.

*cis*TargetX is conceptually similar to the PhylCRM/Lever and ModuleMiner methods for vertebrate genomes [18],[20] and allows determining whether a set of candidate genes, for example a mixture of direct and indirect target genes, is enriched for direct targets of a certain TF or combination of TFs. Compared to other motif discovery methods, such as Clover, PASTAA, PSCAN, and oPOSSUM, *cis*TargetX integrates motif clustering, cross-species comparisons, and whole-genome backgrounds in the discovery process. Additionally, and unlike the vertebrate methods mentioned above, *cis*TargetX focuses on homotypic *CRM*s and therefore allows separating the motif scoring (performed offline) from the gene set enrichment analysis (performed online), yielding a computationally efficient method that can be used as an online Web application. A second difference from PhylCRM/Lever is that once a predicted motif is selected, *cis*TargetX determines the optimal subset of direct TF targets from the input set.

*cis*TargetX was applied to Ato downstream genes identifying novel E-box motifs together with a significant enrichment of predicted direct targets. Although both GOF and LOF analysis yielded significant enrichment of E-boxes in misregulated genes, the significance was higher in the GOF analysis. This higher significance is likely because GOF of Ato results largely in the ectopic gain of one particular cell type, namely the R8 photoreceptor precursor, while the LOF condition results in the loss of all cell types and hence the downregulation of a larger set of genes across numerous cell types.

In the third step we tested several predicted Ato target enhancers in vivo. This procedure identified 20 bona fide Atonal target enhancers out of 39 tested predictions, of which 17 are novel. This relatively high success rate almost certainly represents the lower limit of the true enhancer discovery rate because of false negative experimental results such as cases where the isolated enhancer is insufficient or requires its endogenous proximal promoter. Generally, demonstration of in vivo binding of the TF to a target enhancer that has been shown to be functional would be ideal. However, this is often not feasible, either due to lack of reagents or due to spatially and temporally sparse expression patterns of the TF in question. Our data suggest that *cis*TargetX is a cheap, simple, fast, and high-confidence approach for CRM discovery for any TF.

Finally, it is important to note that 11 of the 20 Ato target genes are known to act in sensory organ development or function, indicating that our approach identifies biologically relevant target genes and that the other nine genes are also players in this process.

### Proneural Target Genes and Evolutionary Implications

A significant portion of the Ato target genes encodes signaling molecules regulating most of the known key developmental pathways such as Notch, EGFR, Wnt, and JNK. Ato activates targets that modulate signaling pathways; thus far no evidence exists that Ato (or, to our knowledge, any other proneural TF) directly activates terminal differentiation genes. Even for molecules like Fas2, long thought to exclusively mediate adhesion during synaptic targeting, recent evidence reveals a role in regulating the precision of EGFR signaling during early photoreceptor specification [51]. While we cannot exclude that we have missed such target genes in this analysis because no approach can be certain of identifying all possible target genes, it is highly unlikely that a specific set of molecular functions would be selected against in an expression analysis approach. We therefore favor the idea that the terminal differentiation genes are activated by other TFs, or by the TFs downstream of the Ato-regulated signaling pathways. It is noteworthy that the pathways regulated by Ato target genes, as well as many of the target genes themselves or their mammalian homologues, such as *sens*, *dap*, *Traf4*, and *Mmp2* are implicated in cancer. We suggest that Ato's functions in cancer [23],[52],[53] is implemented via the regulation of some or all of the targets identified herein.

A remarkable finding is that none of the Ato target enhancers is active in a single sensory organ. Instead, Ato activates a unique combination of targets in each sensory organs it specifies. What kind of target genes can, in a combinatorial fashion, lead to differential morphological and functional development? On the basis of the analysis of the diversity of the beak sizes of Darwin's finches, it has been speculated that evolutionary changes in enhancers of signaling molecules have switch-like effects on a developmental GRN [44]. Our data suggest that variation of the proneural target set driven by changes in the *cis*-regulatory sequences of target genes shapes a unique regulatory state defined by a particular combination of signaling molecules. Interestingly, the Ato response elements within the regulatory sequences of target genes are evolutionarily conserved and their absence appears to alter the expression of these sequences. This observation leads us to hypothesize that a largely common genetic program induces different sensory organs, and that developmental and evolutionary variation of these organs occurs via subtle variations in the *cis*-regulatory sequences of signaling regulators. We propose that similar principles underlie diversification of most, if not all, developmental programs.

### Implications for GRN Mapping

The encouraging results for Atonal lead to the prediction of a large set of target genes for multiple TFs involved in retinal differentiation and they furthermore show that expression studies combined with computational predictions are a powerful tool of regulatory network discovery. The identification of Glass and Su(H) targets from wild-type eye versus wing comparisons of gene expression shows that genetic perturbations of TFs are not a prerequisite to find enriched direct targets in a set of candidate genes, at least for tissue specific TFs. Therefore, from wild-type comparative gene expression experiments meaningful results can be obtained.

The *cis*TargetX analyses in this study compare the enrichment of predicted targets for single motifs (i.e., homotypic enhancer models) within sets of coexpressed genes. The most important advantage of homotypic clusters is that no a priori knowledge of cooperative factors is needed. An additional advantage is that theoretically the predictions can be more specific than “free” heterotypic clusters in which binding sites for any combination of TFs is allowed (the “OR” rule), and more sensitive than the “constrained” class of heterotypic clusters in which all input TFs are required to have binding sites (the “AND” rule). Tests with heterotypic enhancer models, consisting of motif combinations, generally showed lower enrichment than homotypic models (unpublished data), corroborating previous findings [54]. Genes that are activated in the same temporal and spatial patterns do not necessarily share the same *cis*-regulatory code, and the performance of genome-wide predictions may not necessarily benefit from heterotypic enhancer models, mainly because of sensitivity problems, at least in approaches similar to *cis*TargetX that are based on enrichment of direct targets in a candidate gene set. In other words, if many different combinatorial codes exist, then the presence of cofactor sites in only a few enhancers does not yield statistical over-representation and hence does not emerge from the noise. Moreover, coregulation might also occur through different enhancers of the same target genes and we observe many potential examples of this by predicting targets for multiple TFs independently. The important point is that whether coregulation occurs through shared or distinct enhancers, homotypic cluster predictions using *cis*TargetX, followed by comparisons of the targets between the TFs can discover these relationships.

The putative early retinal differentiation network reconstructed from *cis*TargetX predictions shows waves of combinatorial regulation orchestrating spatial and temporal gene expression accuracy. We find two feed-forward loops, namely Ey-Ato and Ato-Sens. These features are similar to the reconstructed regulatory networks underlying early embryonic processes [5],[55]. This finding indicates that exploiting motif predictions in conjunction with expression perturbations allows discovering similar regulatory networks as with ChIP-chip or ChIP-Seq approaches, where more material (e.g., large embryo collections) and specific reagents (e.g., high-quality antibodies) are required. Finally, these predictions represent a useful resource for future experiments aimed at dissecting the mechanistic basis of sensory specification.

## Materials and Methods

### Fly Husbandry

Fly strains used were ato-GAL4 (NP6558), GAL4/7, UAS-ato, UAS-sens (a gift from H. Bellen), UAS-scute (a gift from J. Modolell), dpp-GAL4, yw, M(eGFP.vas-int.Dm) ZH-2A; M(RFP.attP')ZH-22A (a gift from K. Basler); yw, M(eGFP.vas-int.Dm) ZH-2A; y+ attP' VK37, and VK16 (a gift from H. Bellen and K. Venken), CantonS, and yw. All flies were raised at 18°C on standard fly food and vials were transferred to 28°C for 24 h before dissections of imaginal discs.

### Immunohistochemistry

Imaginal discs of wandering third instar larva were dissected and processed as described [56]. Antibodies used were anti-ato antibody (gift from A. Jarman and P. zur Lage), anti-GFP (Invitrogen), and anti-Sens (gift from H. Bellen).

### Imaginal Disc Dissections, RNA Extraction, and qRT-PCR

Dissection of eye-antennal discs was done in RNA Later (Ambion) and RNA extraction with mini RNA isolation kit (ZymoResearch). For relative quantitation of positive control genes (*ato*, *sens*, *sca*, *dap*), we used the comparative ddCt method (SDS User bulletin 2; Applied Biosystems) with the qPCR Mastermix Plus for SYBR Green I (Eurogentec) on the ABI PRISM 7000 instrument. Total RNA was converted to cDNA using QuantiTect Reverse Transcription (Qiagen). Primers were designed with PrimerExpress software (Applied Biosystems) and are available on request. As housekeeping genes we used *rpl32*, *rps13*, and *gapdh*. After an initial denaturation step for 10 min at 95°C, thermal cycling conditions were 15 s at 95°C and 1 min at 60°C for 40 cycles. Eight control samples were extracted, namely two biological repeats for four lines (cantonS wild type, UAS-ato, ato-Gal4, Gal4/7). For Ato GOF, three biological repeats were extracted for atoGal4 × UASato and three for Gal4/7 × UASato (thus six Ato GOF samples in total). For Ato LOF, +;ato^1^/hshid stock was heat-shocked on three consecutive days starting at first instar stage, and three independent repeats were extracted. For *sens* GOF, three atoGal4 × UASsens samples were extracted.

### High-Throughput Examination of Gene Expression

#### Labeling, hybridization, scanning

RNA concentration and purity were determined spectrophotometrically using the Nanodrop ND-1000 (Nanodrop Technologies) and RNA integrity was assessed using a Bioanalyser 2100 (Agilent). Per sample, an amount of 2 µg of total RNA spiked with four bacterial RNA transcripts (Affymetrix) was converted and amplified to double-stranded cDNA in a 1-cycle cDNA reverse transcription reaction. Subsequently, the sample was converted to antisense cRNA and labeled with biotin through an in vitro transcription reaction according to the manufacturers protocol (Affymetrix). All amplification and labeling reactions were performed on a Biomek 3000 ArrayPlex Workstation (Beckman Coulter). A mixture of purified and fragmented biotinylated cRNA and added hybridization controls (Affymetrix) was hybridized on Affymetrix Drosophila 2.0 arrays followed by staining and washing in the GeneChip fluidics station 450 (Affymetrix) according to the manufacturer's procedures. To assess the raw probe signal intensities, chips were scanned using the GeneChip scanner 3000 (Affymetrix).

#### Data analysis

Data analysis was performed with BioConductor in R [57]. Normalization was done with RMA, gcRMA, and MAS5.0. Selection of differentially expressed genes was done with Linear Models for Microarray Data (LIMMA) [58], using Benjamini-Hochberg correction for multiple testing [59]. The set of 204 upregulated genes after Ato GOF are obtained by joining six sets of upregulated genes, obtained by using different preprocessing procedures (RMA, gcRMA, MAS5.0) and filters (RMA AND FDR <0.01; RMA AND FDR <0.05 AND >1.5-fold; GCRMA AND FDR <0.05; MAS5.0 AND FDR <0.05; MAS5.0 AND at least one sample >2-fold AND *t*-test *p*<0.05 AND top100; RMA AND only Ato-GAL4,UAS-Ato AND FDR <0.05). The downregulated gene set after Ato LOF contains 315 genes significantly downregulated in Ato LOF eye-antennal imaginal discs, obtained by [gcRMA AND FDR <0.05 AND >3-fold down].

#### *cis*TargetX

*cis*TargetX consists of two steps (Figure 1). In the first step, the *cis*Target method is used to rank all genes in the genome for their likelihood of being a target gene of a certain input motif, through a combination of motif clustering and comparative genomics [28]. In the second step, the genomic ranks of a set of coexpressed genes are plotted in a cumulative recovery curve, as applied before on similar or related problems [18]–[20],[28],[29],[54]. To determine statistical significance of the recovery curve and to determine the optimal cutoff, the AUC is compared to the distribution of areas under 1,980 control curves obtained by ranking all genes for a large collection of control motifs (Table S1). *cis*TargetX is illustrated for a positive control TF (Text S1), is validated for several other TFs (Table 1), and is available at http://med.kuleuven.be/cme-mg/lng/cisTargetX.

### Motif Over-Representation Analysis and Enhancer Visualization

Motif prediction in sets of related enhancers, such as the 21 Ato target enhancers in Figure 6, are performed with Clover [14], using all 5-kb upstream and intronic sequences as background sequences and using 10,000 randomizations (−*r* 10,000). Clover output is transformed to GFF format using a perl script. Visualization of enhancers and predicted binding sites is done in TOUCAN [60]. All Clover motifs are shown with motif score greater than 6 (default Clover parameter). The Ato binding-site predictions that were mutated (Figure 3B) are those given by Cluster-Buster with the Ato-PWM, with motif score greater than 6 (default Cluster-Buster parameter).

### Data Availability

Microarray data are available from the Gene Expression Omnibus as Series GSE16713 (http://www.ncbi.nlm.nih.gov/geo/query/acc.cgi?acc=GSE16713). Positive and negative enhancer data will available from the REDfly (http://redfly.ccr.buffalo.edu/) [61] and ORegAnno (http://www.oreganno.org) [62] databases of regulatory annotation.

## Supporting Information

Figure S1 ***cis*** **TargetX homotypic versus heterotypic example.** Recovery curves for a set of 80 genes expressed downstream of Dorsal, using the Dorsal motif alone (Jaspar PWM MA0022) as homotypic model, or using the Dorsal motif together with the twist motif (PWM from FlyReg [63]). For “MA0022 AND twi,” the Cluster-Buster predictions are filtered retaining only CRM predictions with matches to both PWMs. For “MA0022 OR twi”, the Cluster-Buster predictions are not filtered, hence retaining CRMs with matches to MA0022, twi, or both.
(3.92 MB TIF)

Figure S2 **qRT-PCR in eye-antennal imaginal discs after Ato overexpression.** Ato overexpression causes upregulation of *ato*, *sens*, *dap*, and *sca*, validating the ectopic overexpression of Atonal, the dissection of eye-antennal imaginal discs, and the RNA extraction.
(1.73 MB TIF)

Figure S3 **Creation of a new enhancer-reporter vector.** (A) The “pH-attB-Dest” was created by inserting an attB attachment site—for phiC31 integration-mediated transgenesis—and a Gateway cassette into the pHStinger [64] vector. AttB is phiC31 attachment site; I is gypsy insulator; hsp70 is the proximal promoter of *Hsp70*. (B) The novel vector was tested using two known target enhancers of *ato*. Left: The eye enhancer of *dacapo* (*dap-HB* [30]). Right: The auto-regulatory chordotonal enhancer of *ato* [42]. Both enhancers show the correct expression pattern, namely the posterior part of the eye disc for *dap-HB* and the femoral chordotonal organ progenitors for the *ato* enhancer.
(1.62 MB TIF)

Figure S4 **Enhancer-GFP Ato target enhancer activity in eye-antennal imaginal discs.** Enhancer activity in the eye-antennal imaginal disc shown by immunohistochemistry against GFP, Ato, and Sens. Green, GFP; red, Ato antibody; blue, Sens antibody.
(5.13 MB TIF)

Figure S5 **Fragment size controls.** (A) Comparison of the tested fragment sizes between positive and negative Ato target enhancers, showing no significant difference between the groups (*p* = 0.15). (B) Comparison of a 2-kb (SBg) fragment (left) and a 5.6-kb XBg fragment (right), containing the ato autoregulatory enhancer with reporter expression in the chordotonal organ precursors (white arrow), showing that longer fragments generate ectopic expression rather than fewer expression, arguing against the possible lack of repressor elements when testing relatively short fragments.
(2.83 MB TIF)

Figure S6 **Ectopic enhancer-GFP.** Green, GFP; red, Ato antibody; blue, Sens antibody. Enhancer-reporter activated ectopically by Ato in the wing imaginal disc along the antero-posterior boundary using *dppGAL4,UAS-Ato*. Enhancers of *Dscam*, *Fas2*, *Pde8*, *CG30492*, *sca*, *Spn*, *nmo*, *Traf1*, *spdo*, *siz*, *neur*, *m4*, *E(spl)*, *sens*, *dap*, and *ato* (not shown) can be ectopically activated.
(10.89 MB TIF)

Figure S7 **Validation of Fas2_E1E2mut enhancer.** (A) The wild-type Fas2 enhancer can be activated ectopically by Ato using *dpp-GAL4*, *UAS-Ato* (left), while the mutated Fas2 enhancer cannot (right). (B) qRT-PCR for reporter-GFP mRNA. The difference in GFP mRNA levels is shown between wild-type Fas2 enhancer and the mutated Fas2 enhancer.
(2.25 MB TIF)

Figure S8 **Enhancer-reporters in wild-type wing imaginal discs.** Activity of the identified Ato target enhancers revealed by a GFP reporter assay. All but CG1625 and sens show expression in the chordotonal organ. Green, GFP; red, Ato; blue, Sens.
(2.46 MB TIF)

Figure S9 **Enhancer-reporters in wild-type leg imaginal discs.** Activity of the 15 newly identified Ato target enhancers revealed by a GFP reporter assay. All but CG1625 show expression in the femoral chordotonal organ. Green, GFP; red, Ato; blue, Sens.
(10.29 MB TIF)

Figure S10 ***cis*** **TargetX results.** (A) GOF-set and RACASCTGY. GOF-set contains 204 genes significantly upregulated in Ato GOF eye-antennal discs. ROC is plotted from RACASCTGY-based genomic rankings. (B) LOF-set and RACASCTGY. LOF-set contains for 315 genes significantly downregulated (>3-fold; FDR <0.05) from Ato LOF microarray data in the eye-antennal imaginal discs. (C) Genes upregulated by eyeless [45] and the ey PWM [45]. (D) Genes upregulated by Ato and a Su(H) PWM from TRANSFAC (M00234). (E) Genes significantly upregulated by senseless and senseless consensus motif [65]. (F) Genes downregulated by senseless and senseless PWM predicted from the C2H2 zinc finger protein structure [48].
(2.41 MB TIF)

Figure S11 **Predicted GRN underlying early retinal differentiation.** Lines (edges) are drawn from several TFs to their predicted target genes. An edge between a TF and a target indicates that (1) the target is significantly misregulated when the TF is perturbed genetically; and (2) that motif predictions using a PWM for the TF have led to significantly high ranking of the target, compared to other genes in the genome, and compared to PWMs of other TFs.
(1.87 MB TIF)

Table S1 **PWM libraries.** (A) The libraries of PWMs that have been used for the Atonal target gene predictions using *cis*TargetX. (B) Several more recent libraries are available through the online application and can be used for analysis, such as those based on protein binding microarrays.
(0.07 MB PDF)

Table S2 **cisTargetX results for a set of 80 genes expressed downstream of dorsal (dl).** The best motifs are all variations of the dorsal (NFkB) motif.
(1.24 MB PDF)

Table S3 **Upregulated genes in Ato GOF.** SetA consists of 204 genes that are significantly upregulated in six Ato GOF samples versus eight control samples.
(0.09 MB PDF)

Table S4 ***cis*** **TargetX results for 204 Ato-upregulated genes.** The best motif out of 1,981 tested motifs is RACASCTGY.
(2.57 MB PDF)

Table S5 **Count matrix representing the Atonal binding site.** We constructed a “phyloPWM” [28],[45] for Atonal in TOUCAN [60] using known Ato [42], Brd [31], TakR86C [66], Math1 [67], and ATH5 binding sites [68], including aligned and conserved binding sites from other species, obtained from UCSC Genome Browser alignments.
(0.05 MB PDF)

Table S6 **Direct Ato target gene predictions.** Subset of Ato-misregulated genes (GOF and LOF) obtained from various *cis*TargetX analyses (see text for details).
(0.07 MB PDF)

Table S7 ***cis*** **TargetX results for 315 Ato-downregulated genes in ato^−/−^ eye-antennal discs.** The best motifs are Su(H) motifs; E-box motifs are also significantly over-represented, such as RACASCTGY.
(2.07 MB PDF)

Table S8 **Primers for candidate Ato target enhancers.** Positive Ato target enhancers are shaded in green. The boundaries of a CRM are determined automatically by Cluster-Buster, and the flanking sequence of the CRM was determined manually using the multiz alignments across 12 *Drosophila* species in the UCSC Genome Browser. Primers were designed in regions with low conservation, to amplify an enclosing genomic region with overall high sequence conservation, including the predicted CRM. For sens, E(spl), m4, and siz published reporter lines were used [32],[36],[37].
(0.07 MB PDF)

Table S9 **Genomic location and size of candidate Atonal target enhancers. **Positive Ato target enhancers are shaded in green.
(0.07 MB PDF)

Table S10 **Predicted** ***cis*** **-regulatory interactions in the transcriptional network underlying early retinal differentiation.** Interactions in italics are drawn from the literature, while all other predictions result from *cis*TargetX analyses described in this study. This list of interactions is used directly as input for network mapping in the BioTapestry software (see Figure S11).
(0.12 MB PDF)

Table S11 **Over-represented motifs among 21 Ato target enhancers.** The enhancer set comprises the 17 novel Ato targets from the Ato GOF and LOF analysis plus the rediscovered sens, ato, and dap enhancers, plus the previously known Brd enhancer. Over-represented motifs were determined by Clover [14]. The background sequence used for Clover, to select random sequence sets from, was the set of all 5-kb upstream and intronic regions. The number of randomizations was set to 10,000. The PWM collection used for Clover is the same as the basic collection of 1,981 PWMs used by the *cis*TargetX analyses, from Table S1A.
(0.09 MB PDF)

Text S1 **Overview of** ***cis*** **TargetX using dorsal (dl) as an example.**
(0.21 MB PDF)

Text S2 **Comparison of** ***cis*** **TargetX with other methods.**
(0.33 MB PDF)

## Abbreviations

AUC: area under the curve
ChIP: chromatin immunoprecipitation
ChIP-chip: hybridization on a chip
CRM: *cis*-regulatory module
GFP: green fluorescent protein
GOF: gain of function
GRN: gene regulatory network
LOF: loss of function
PWM: position weight matrix
qRT-PCR: quantitative reverse transcription PCR
SOP: sensory organ precursor
TF: transcription factor
