# Supplementary figures and images for: Robust Target Gene Discovery through Transcriptome Perturbations and Genome-Wide Enhancer Predictions in Drosophila Uncovers a Regulatory Basis for Sensory Specification
Source: PLoS Biol. 2010 Jul 27;8(7):e1000435. doi: 10.1371/journal.pbio.1000435 (PMC2910651; doi:10.1371/journal.pbio.1000435)

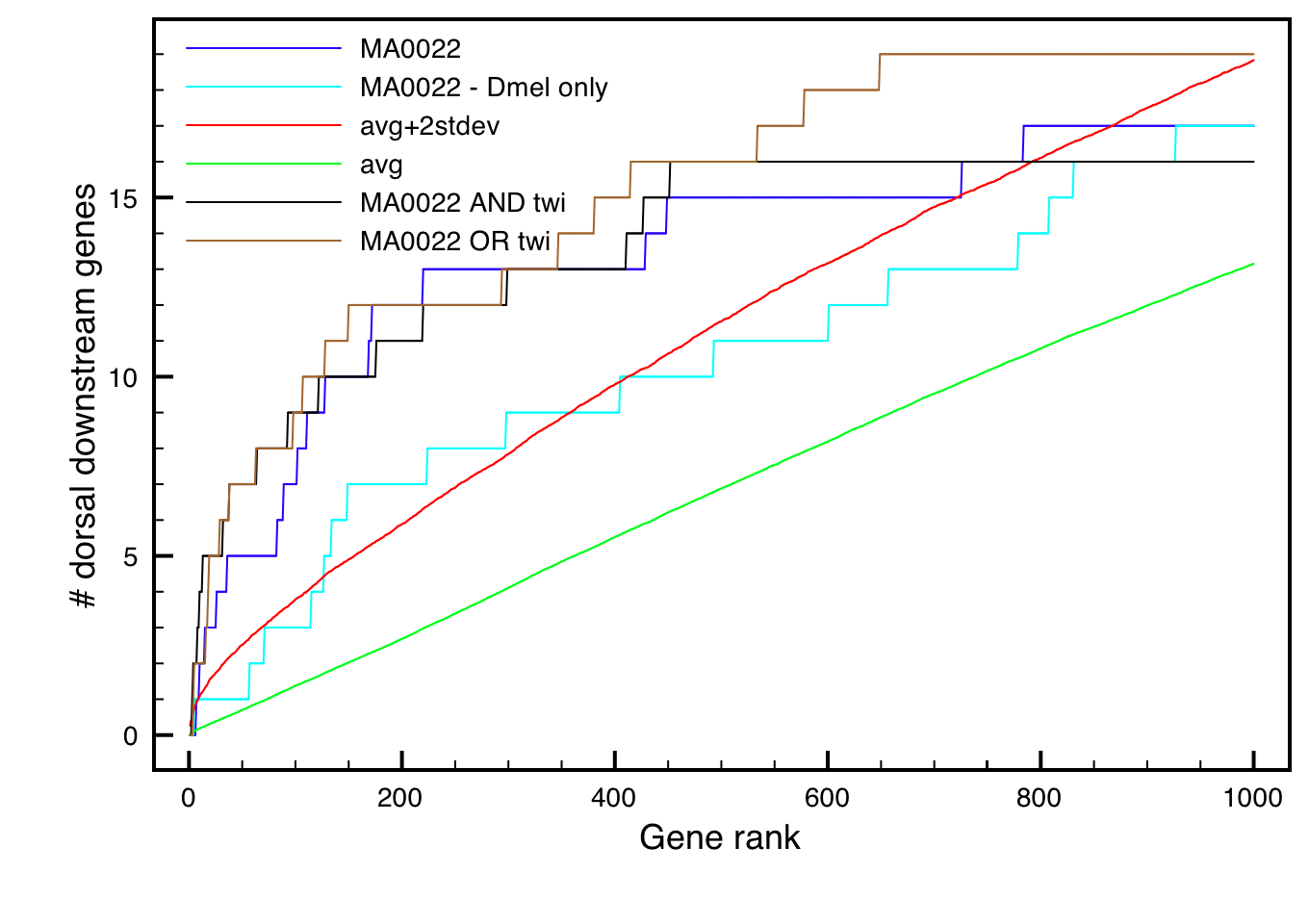

Supplement: Figure S1 — cis TargetX homotypic versus heterotypic example. Recovery curves for a set of 80 genes expressed downstream of Dorsal, using the Dorsal motif alone (Jaspar PWM MA0022) as homotypic model, or using the Dorsal motif together with the twist motif (PWM from FlyReg [63]). For “MA0022 AND twi,” the Cluster-Buster predictions are filtered retaining only CRM predictions with matches to both PWMs. For “MA0022 OR twi”, the Cluster-Buster predictions are not filtered, hence retaining CRMs with matches to MA0022, twi, or both. (3.92 MB TIF) [file pbio.1000435.s001.tif]

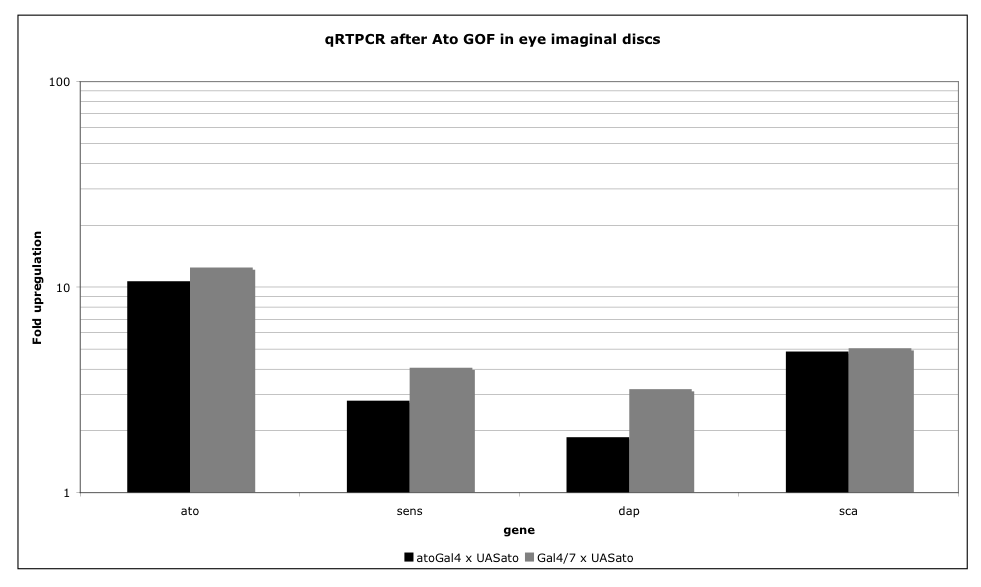

Supplement: Figure S2 — qRT-PCR in eye-antennal imaginal discs after Ato overexpression. Ato overexpression causes upregulation of ato, sens, dap, and sca, validating the ectopic overexpression of Atonal, the dissection of eye-antennal imaginal discs, and the RNA extraction. (1.73 MB TIF) [file pbio.1000435.s002.tif]

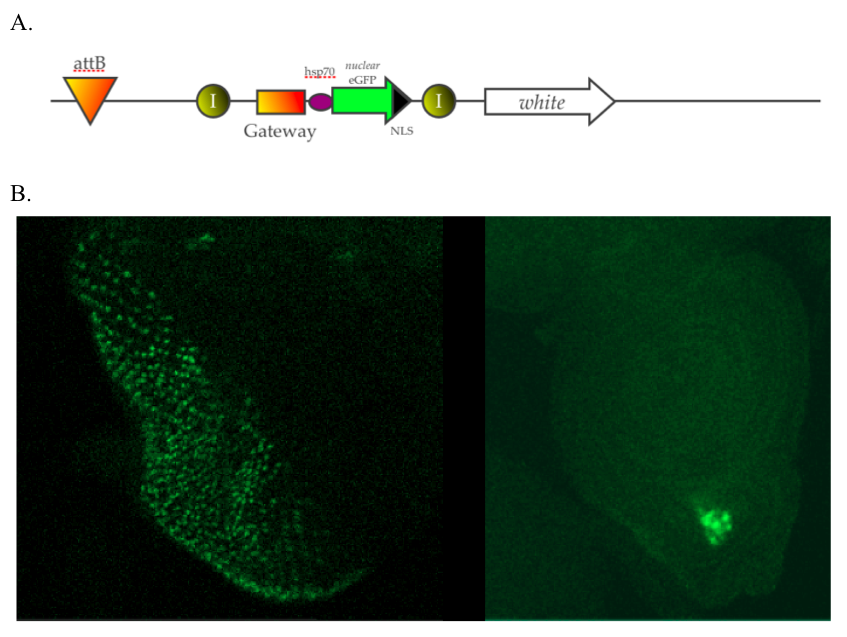

Supplement: Figure S3 — Creation of a new enhancer-reporter vector. (A) The “pH-attB-Dest” was created by inserting an attB attachment site—for phiC31 integration-mediated transgenesis—and a Gateway cassette into the pHStinger [64] vector. AttB is phiC31 attachment site; I is gypsy insulator; hsp70 is the proximal promoter of Hsp70. (B) The novel vector was tested using two known target enhancers of ato. Left: The eye enhancer of dacapo (dap-HB [30]). Right: The auto-regulatory chordotonal enhancer of ato [42]. Both enhancers show the correct expression pattern, namely the posterior part of the eye disc for dap-HB and the femoral chordotonal organ progenitors for the ato enhancer. (1.62 MB TIF) [file pbio.1000435.s003.tif]

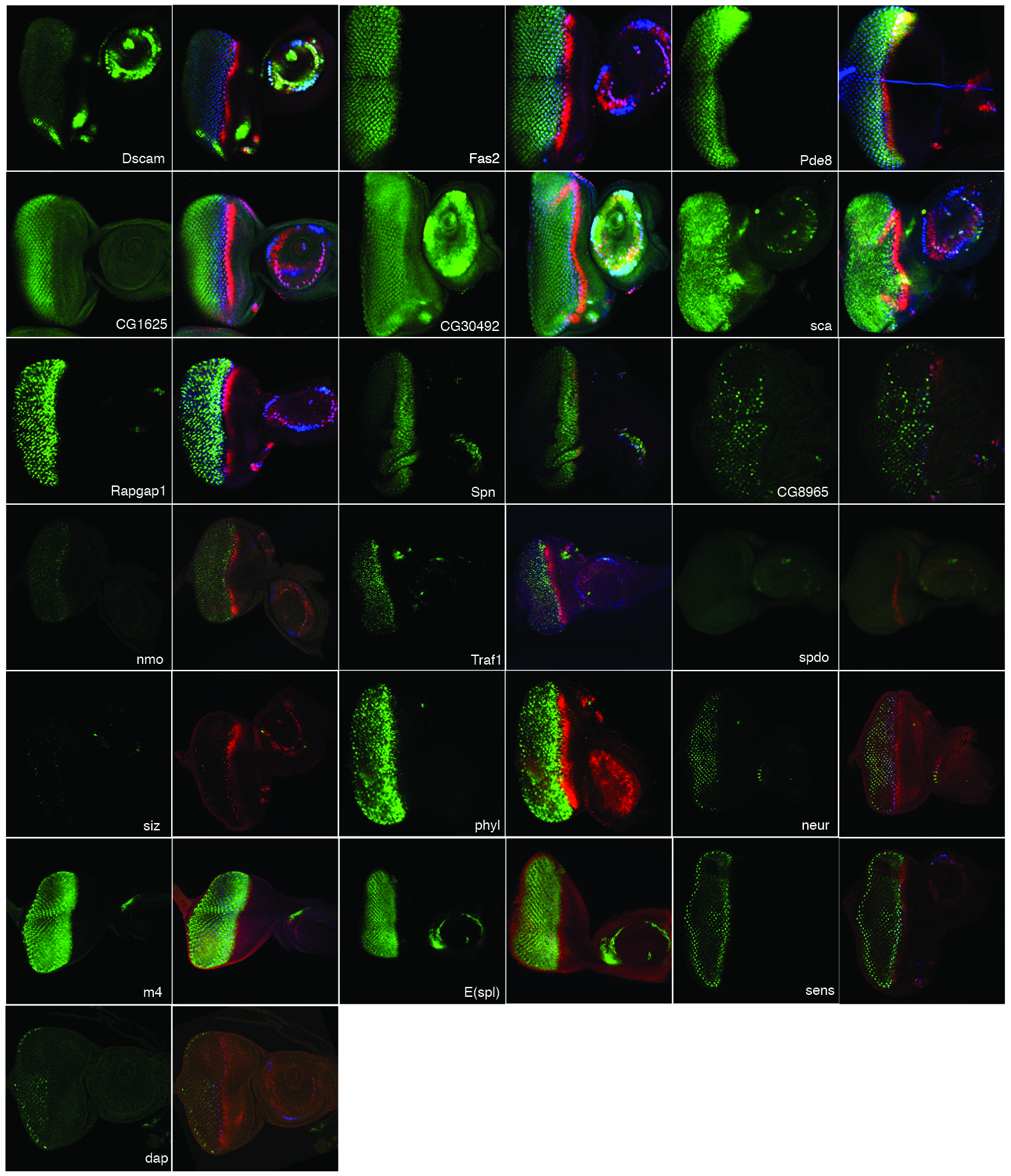

Supplement: Figure S4 — Enhancer-GFP Ato target enhancer activity in eye-antennal imaginal discs. Enhancer activity in the eye-antennal imaginal disc shown by immunohistochemistry against GFP, Ato, and Sens. Green, GFP; red, Ato antibody; blue, Sens antibody. (5.13 MB TIF) [file pbio.1000435.s004.tif]

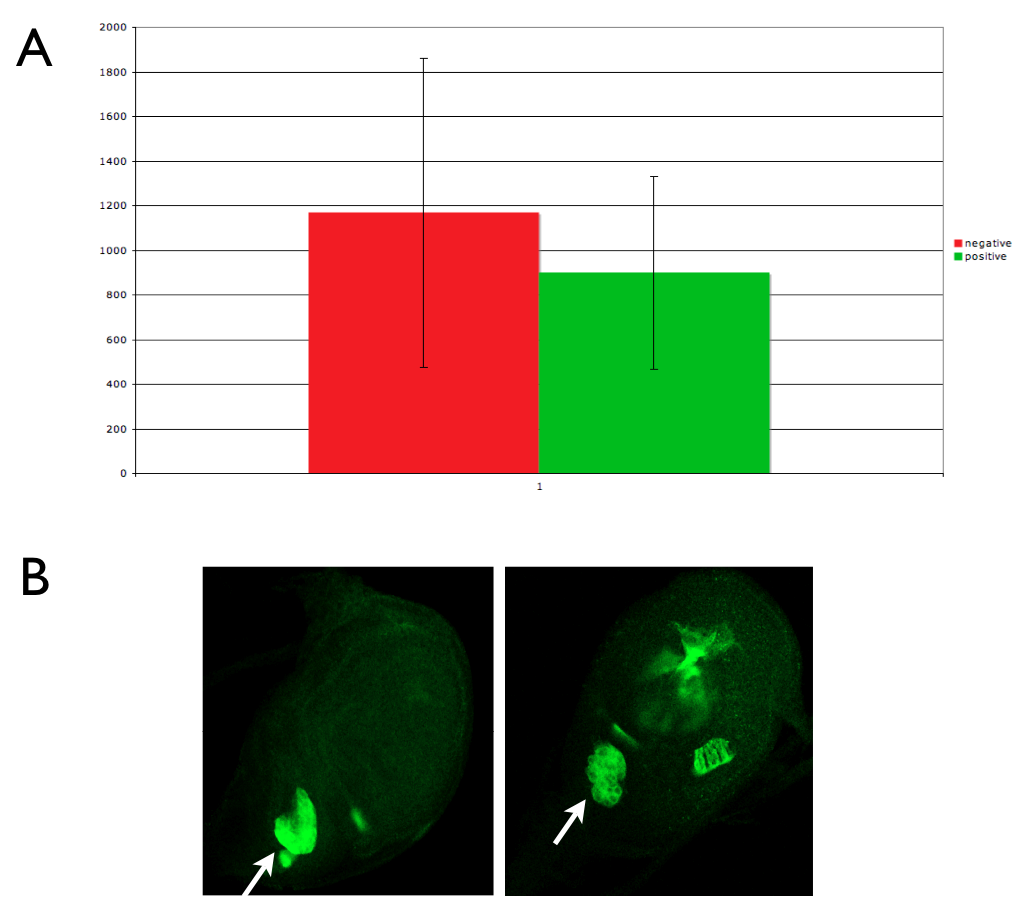

Supplement: Figure S5 — Fragment size controls. (A) Comparison of the tested fragment sizes between positive and negative Ato target enhancers, showing no significant difference between the groups (p = 0.15). (B) Comparison of a 2-kb (SBg) fragment (left) and a 5.6-kb XBg fragment (right), containing the ato autoregulatory enhancer with reporter expression in the chordotonal organ precursors (white arrow), showing that longer fragments generate ectopic expression rather than fewer expression, arguing against the possible lack of repressor elements when testing relatively short fragments. (2.83 MB TIF) [file pbio.1000435.s005.tif]

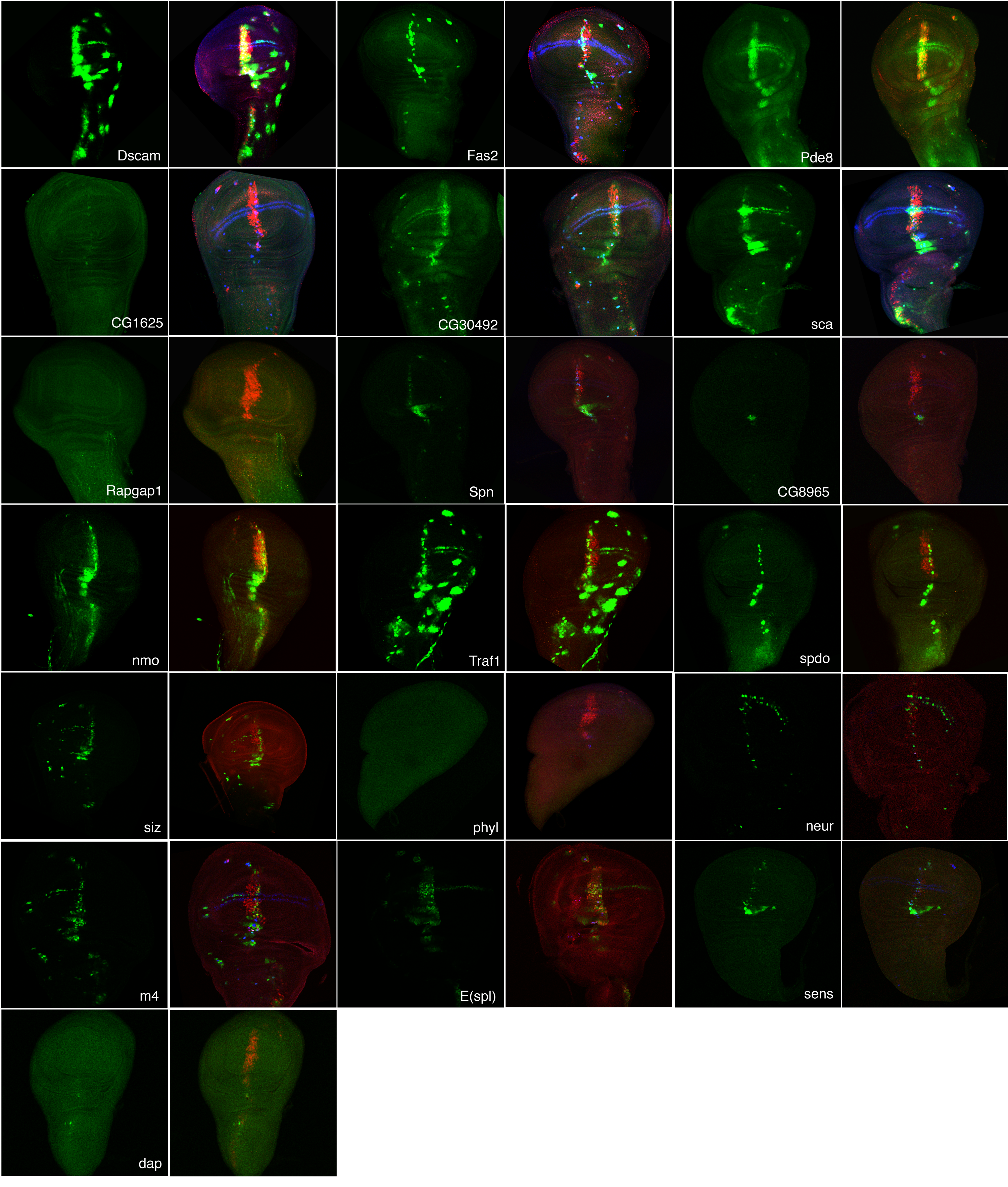

Supplement: Figure S6 — Ectopic enhancer-GFP. Green, GFP; red, Ato antibody; blue, Sens antibody. Enhancer-reporter activated ectopically by Ato in the wing imaginal disc along the antero-posterior boundary using dppGAL4,UAS-Ato. Enhancers of Dscam, Fas2, Pde8, CG30492, sca, Spn, nmo, Traf1, spdo, siz, neur, m4, E(spl), sens, dap, and ato (not shown) can be ectopically activated. (10.89 MB TIF) [file pbio.1000435.s006.tif]

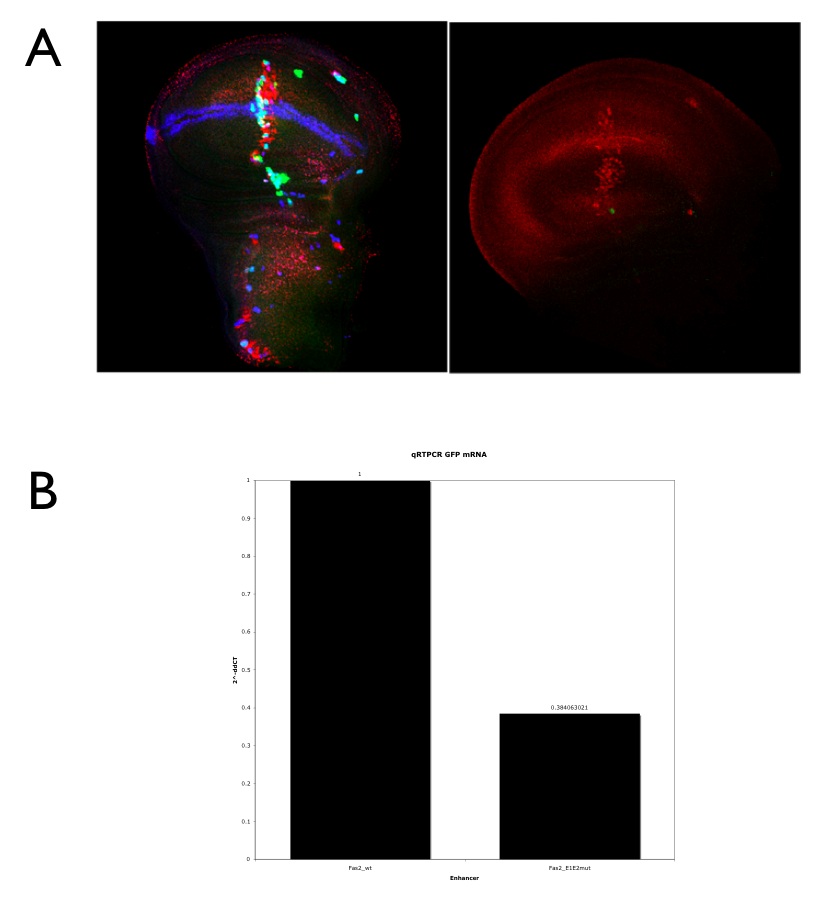

Supplement: Figure S7 — Validation of Fas2_E1E2mut enhancer. (A) The wild-type Fas2 enhancer can be activated ectopically by Ato using dpp-GAL4, UAS-Ato (left), while the mutated Fas2 enhancer cannot (right). (B) qRT-PCR for reporter-GFP mRNA. The difference in GFP mRNA levels is shown between wild-type Fas2 enhancer and the mutated Fas2 enhancer. (2.25 MB TIF) [file pbio.1000435.s007.tif]

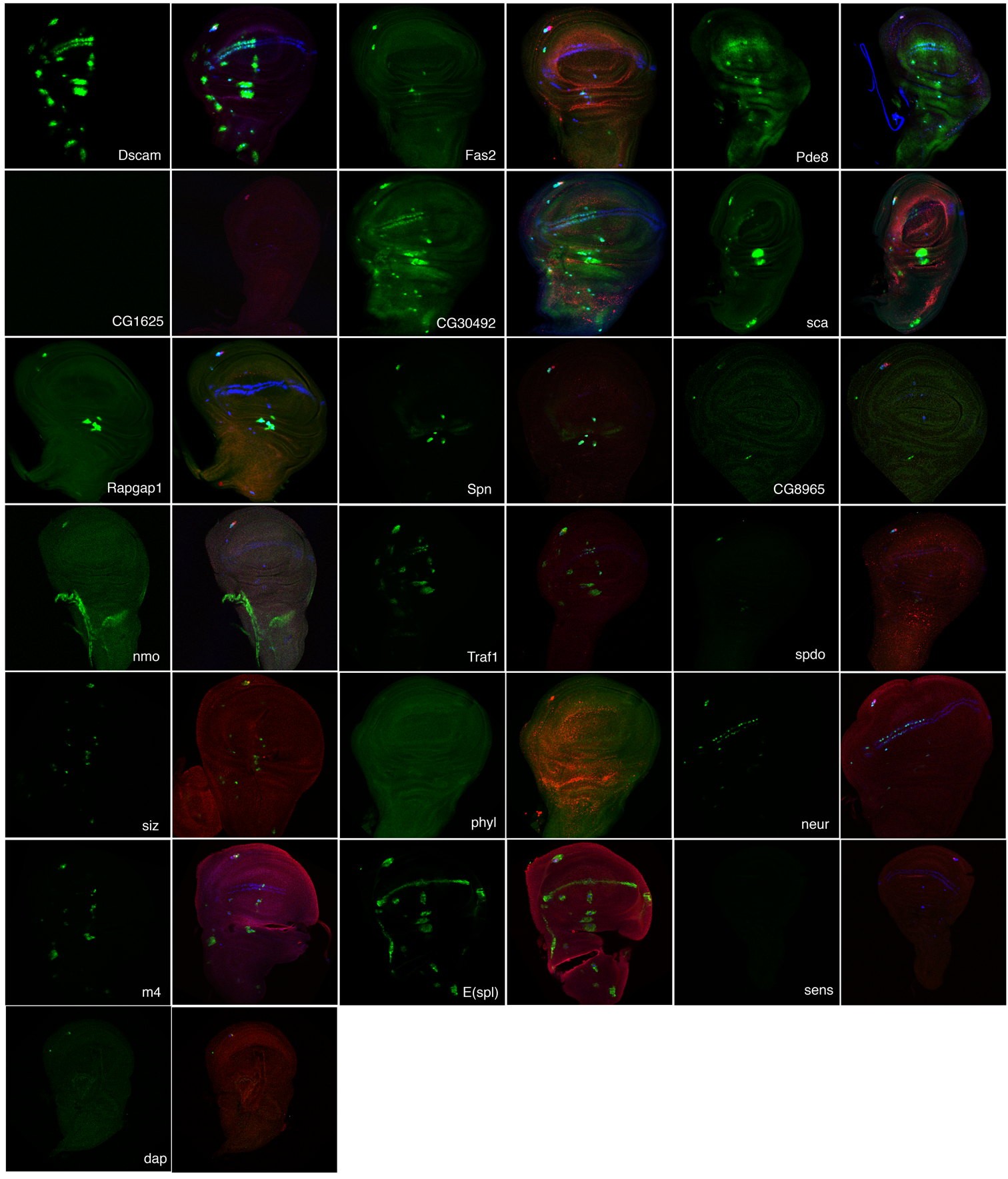

Supplement: Figure S8 — Enhancer-reporters in wild-type wing imaginal discs. Activity of the identified Ato target enhancers revealed by a GFP reporter assay. All but CG1625 and sens show expression in the chordotonal organ. Green, GFP; red, Ato; blue, Sens. (2.46 MB TIF) [file pbio.1000435.s008.tif]

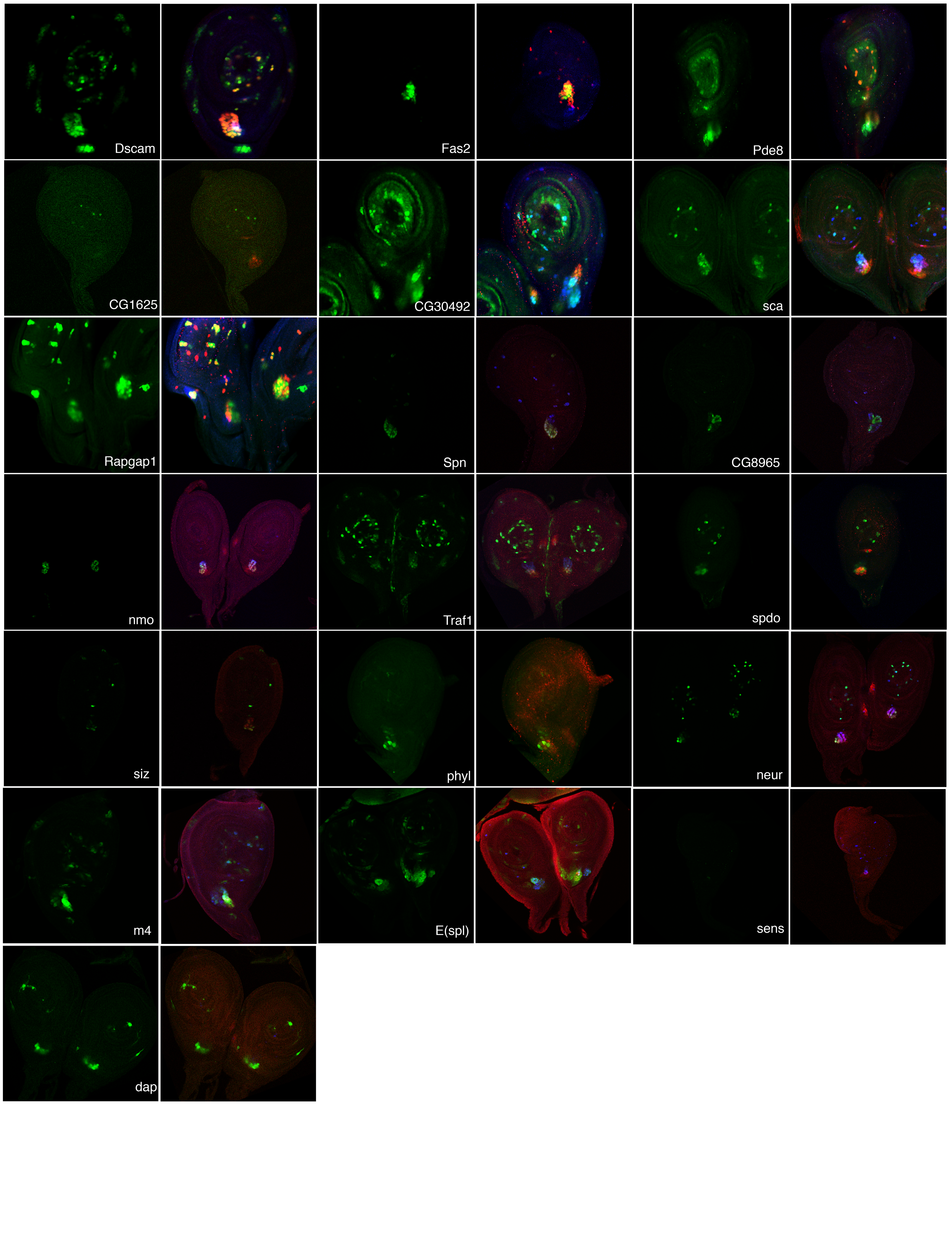

Supplement: Figure S9 — Enhancer-reporters in wild-type leg imaginal discs. Activity of the 15 newly identified Ato target enhancers revealed by a GFP reporter assay. All but CG1625 show expression in the femoral chordotonal organ. Green, GFP; red, Ato; blue, Sens. (10.29 MB TIF) [file pbio.1000435.s009.tif]

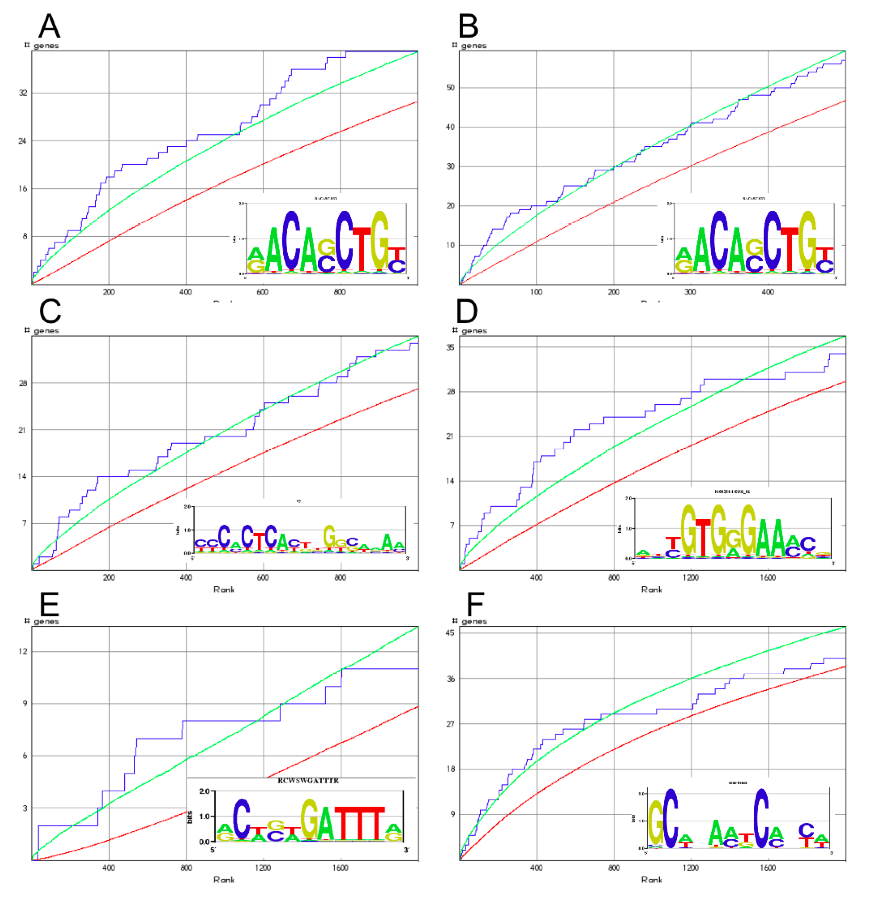

Supplement: Figure S10 — cis TargetX results. (A) GOF-set and RACASCTGY. GOF-set contains 204 genes significantly upregulated in Ato GOF eye-antennal discs. ROC is plotted from RACASCTGY-based genomic rankings. (B) LOF-set and RACASCTGY. LOF-set contains for 315 genes significantly downregulated (>3-fold; FDR <0.05) from Ato LOF microarray data in the eye-antennal imaginal discs. (C) Genes upregulated by eyeless [45] and the ey PWM [45]. (D) Genes upregulated by Ato and a Su(H) PWM from TRANSFAC (M00234). (E) Genes significantly upregulated by senseless and senseless consensus motif [65]. (F) Genes downregulated by senseless and senseless PWM predicted from the C2H2 zinc finger protein structure [48]. (2.41 MB TIF) [file pbio.1000435.s010.tif]

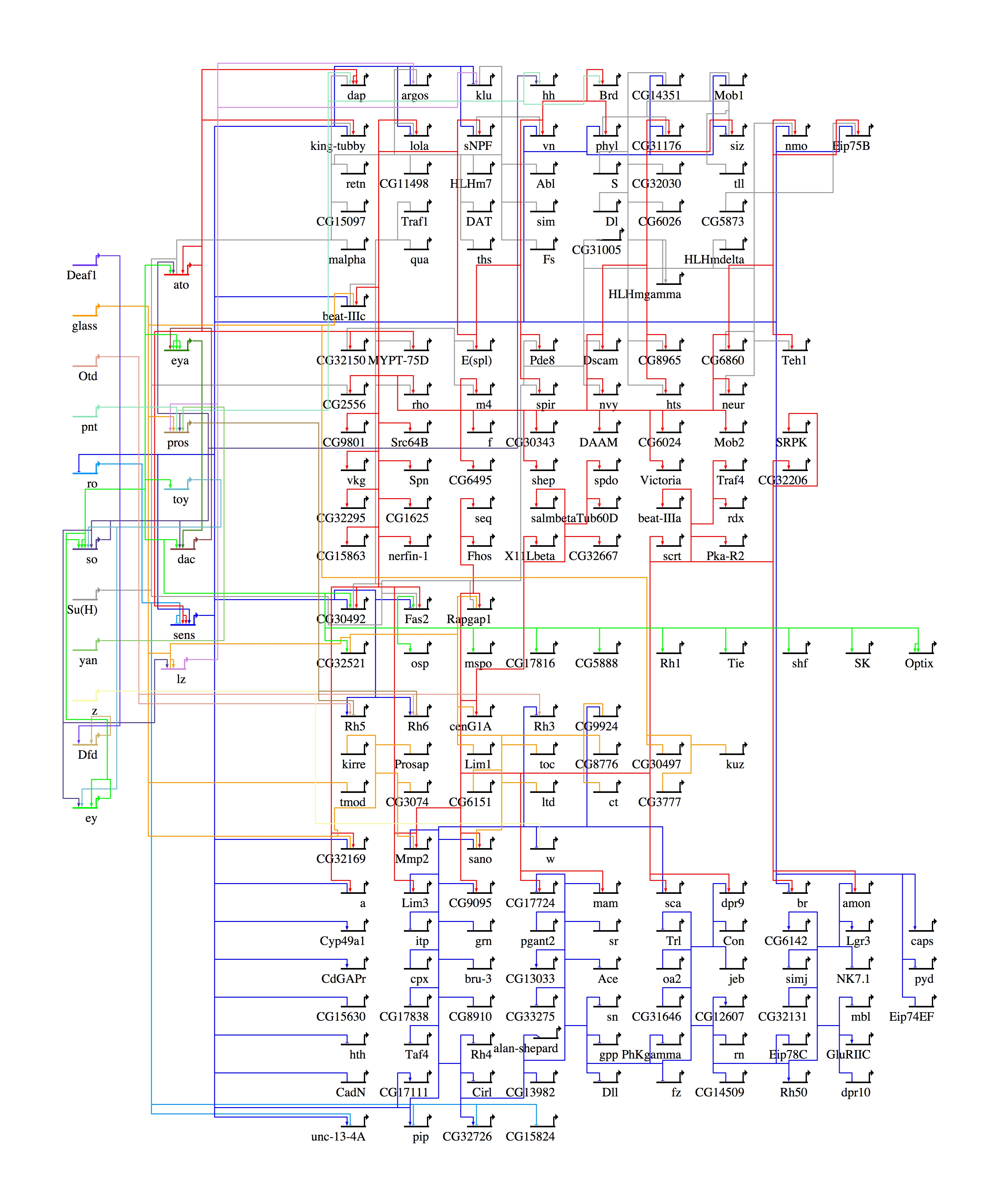

Supplement: Figure S11 — Predicted GRN underlying early retinal differentiation. Lines (edges) are drawn from several TFs to their predicted target genes. An edge between a TF and a target indicates that (1) the target is significantly misregulated when the TF is perturbed genetically; and (2) that motif predictions using a PWM for the TF have led to significantly high ranking of the target, compared to other genes in the genome, and compared to PWMs of other TFs. (1.87 MB TIF) [file pbio.1000435.s011.tif]
