# Supplementary material for: Robust Target Gene Discovery through Transcriptome Perturbations and Genome-Wide Enhancer Predictions in Drosophila Uncovers a Regulatory Basis for Sensory Specification
Source: PLoS Biol. 2010 Jul 27;8(7):e1000435. doi: 10.1371/journal.pbio.1000435 (PMC2910651; doi:10.1371/journal.pbio.1000435)
Supplement: Table S1 — PWM libraries. (A) The libraries of PWMs that have been used for the Atonal target gene predictions using cisTargetX. (B) Several more recent libraries are available through the online application and can be used for analysis, such as those based on protein binding microarrays. (0.07 MB PDF) [file pbio.1000435.s012.pdf]

Supplementary Table S1

PWM libraries. The libraries of PWMs that have been used for the Atonal target gene predictions using CISTARGETX are listed in Table S1A. Table S1B lists several more recent libraries that are available through the online application and can be used for analysis, such as those based on Protein Binding Microarrays. [a] PWMs made from FlyReg sites by Dan Pollard.

**Library/source**	**Description**	**Number of PWMs**
(A)		
FlyReg [1] [a]	Known TFs, DNase I footprints	72
SelexConsensus [2]	Known TFs, SELEX or known binding sites	59
Elemento word library [3]	Whole-genome motif discovery by network-level conservation Dmel-Dpse	371
Stark word library [4]	Whole-genome motif discovery by alignment conservation	230 (232 minus 2 that overlap w/ Elemento)
Tiffin DB [2]	Whole-genome motif discovery by over-representation	120
TRANSFAC pro 10.4 [5]		815
JASPAR core [6]	The original JASPAR database with curated PWMs, mostly based on SELEX.	123
JASPAR Phylofacts [7,8]	Whole-genome motif discovery by conservation in vertebrates	174
JASPAR Fam [9]	One PWM for each TF family	11
Other PWMs [10,11]	4 versions of atoPWM (3,5,7,atolegsite), pros, ey	6
SubTotal		1981
(B) The following PWM libraries are also included in the web application but have not been used in the analysis of proneural targets and the retinal differentiation network.		
Noyes Homeodomain [12]		84
Berger Homeodomain [13]	PWMs from Protein Binding Microarrays: Human + Mouse homeodomain factors	178
Badis mouse [14]	PBM	104
Total		2347
