# Supplementary material for: Robust Target Gene Discovery through Transcriptome Perturbations and Genome-Wide Enhancer Predictions in Drosophila Uncovers a Regulatory Basis for Sensory Specification
Source: PLoS Biol. 2010 Jul 27;8(7):e1000435. doi: 10.1371/journal.pbio.1000435 (PMC2910651; doi:10.1371/journal.pbio.1000435)
Supplement: Table S2 — cisTargetX results for a set of 80 genes expressed downstream of dorsal (dl). The best motifs are all variations of the dorsal (NFkB) motif. (1.24 MB PDF) [file pbio.1000435.s013.pdf]

cisTargetX AUC results

Your list contained 80 genes
You can select one or more motifs and proceed to Cluster-Buster enhancer predictions across 12 species

Motif	Z-score	Logo	ROC	Candidate targets	All genes in top 1000	Select
M00043-I-DL_01	6.04150318054204	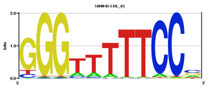	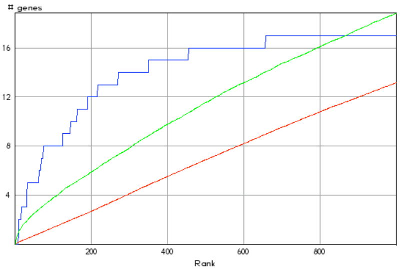	link	link	M00043-I-DL_01
MA0101	5.84418808016455	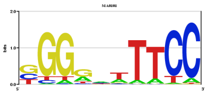	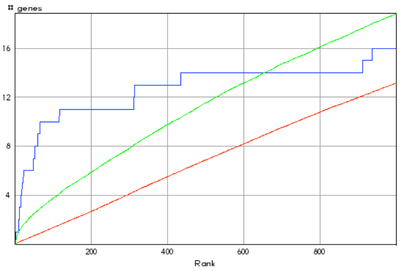	link	link	MA0101
MA0022	5.60968796087042	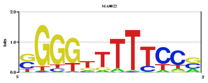	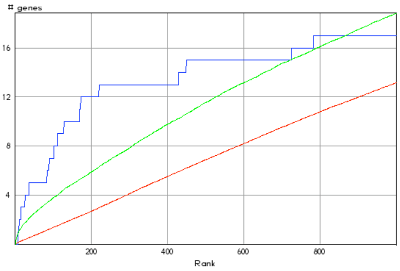	link	link	MA0022
M00053-V-CREL_01	5.27654890250819	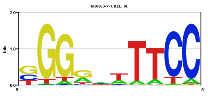	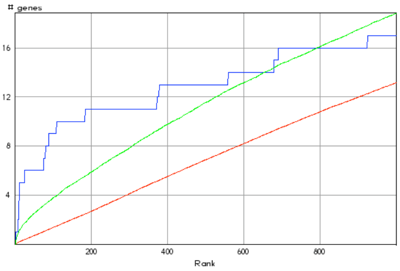	link	link	M00053-V-CREL_01
GGGGAMWWCCM-schnurri	5.10908603953927	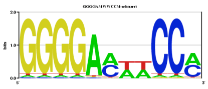	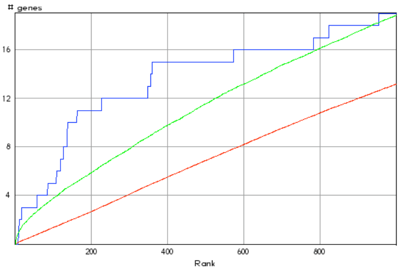	link	link	GGGGAMWWCCM-schnurri

SelexConsensus_shn-ZFP2	4.9620209647248	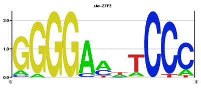	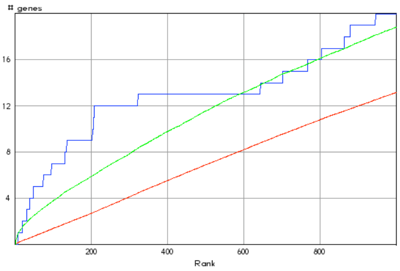	link	link	SelexConsensus_shn-ZFP2
SelexConsensus_dl-B	4.70334499979872	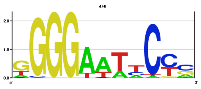	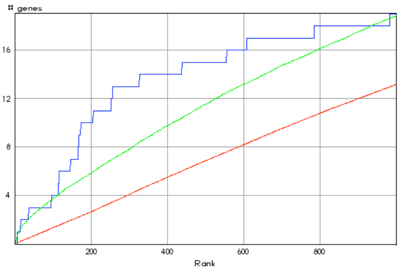	link	link	SelexConsensus_dl-B
MA0107	4.55441881292637	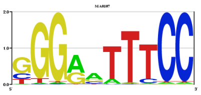	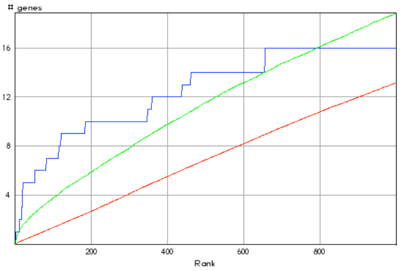	link	link	MA0107
M00052-V-NFKAPPAB65_01	4.49114100295808	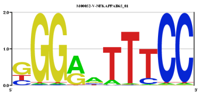	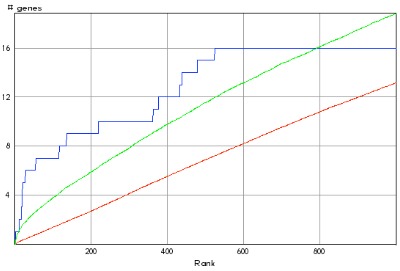	link	link	M00052-V-NFKAPPAB65_01
MA0061	4.45576126273762	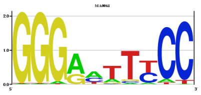	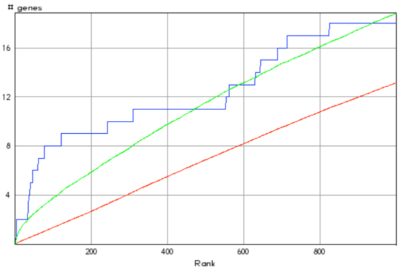	link	link	MA0061
M00194-V-NFKB_Q6	4.39808540006361	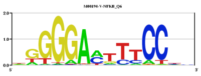	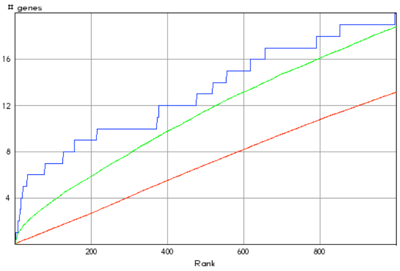	link	link	M00194-V-NFKB_Q6

PF0040	4.377650389668	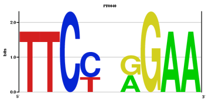	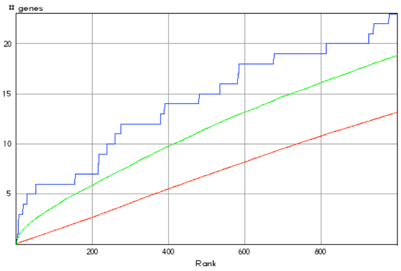	link	link	PF0040
M00054-V-NFKAPPAB_01	4.34409453926425	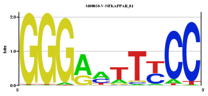	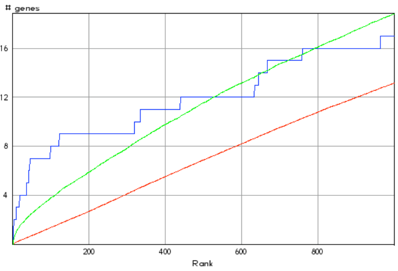	link	link	M00054-V-NFKAPPAB_01
M00774-V-NFKB_Q6_01	4.26408533189558	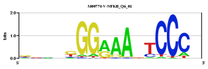	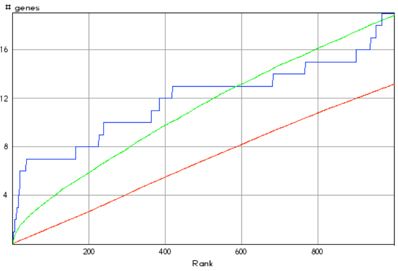	link	link	M00774-V-NFKB_Q6_01
CAGGTAG	4.23806698532627	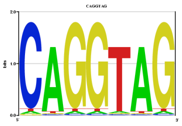	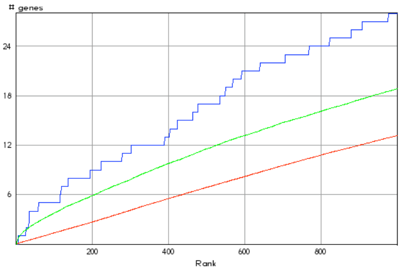	link	link	CAGGTAG
MA0023	3.95512211916527	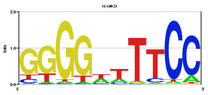	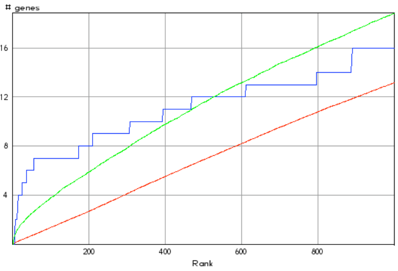	link	link	MA0023
M00208-V-NFKB_C	3.8806590257291	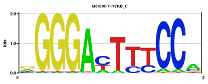	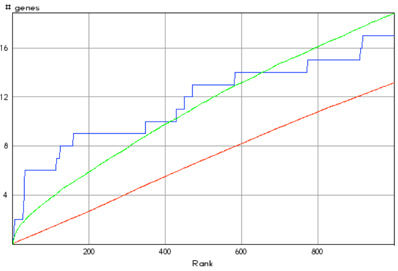	link	link	M00208-V-NFKB_C

dl	3.33163096865161	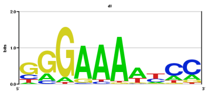	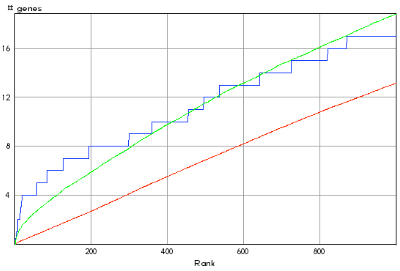	link	link	dl
M00457-V-STAT5A_01	3.32236263060332	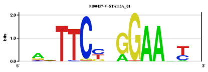	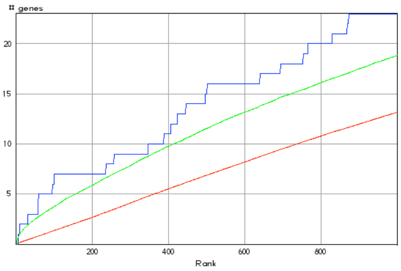	link	link	M00457-V-STAT5A_01
TIFDMEM0000055	3.17903839102523	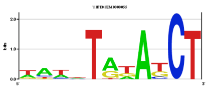	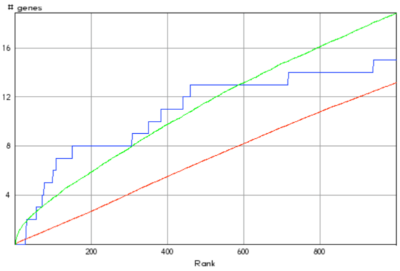	link	link	TIFDMEM0000055
PF0076	3.1436958730459	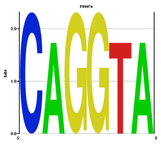	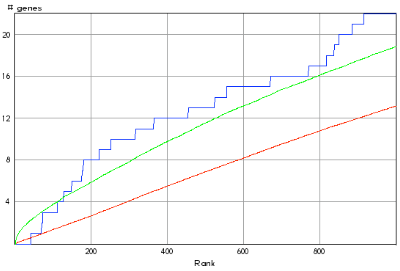	link	link	PF0076
MA0109	3.11203835694119	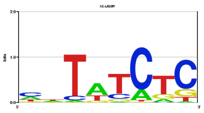	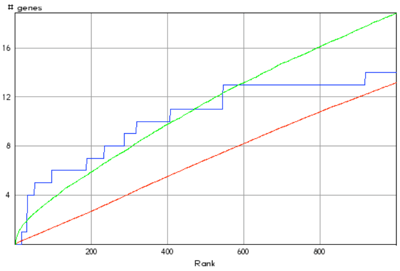	link	link	MA0109
tin	3.09344584748288	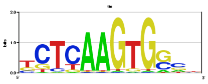	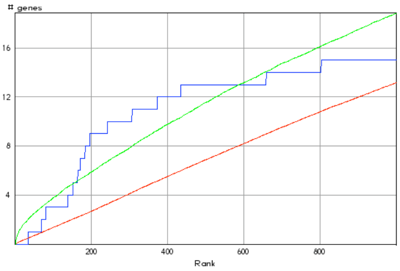	link	link	tin

M00223-V-STAT_01	2.94453827173111	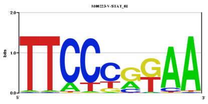	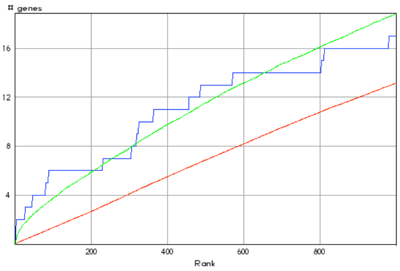	link	link	M00223-V-STAT_01
M00432-V-TITF1_Q3	2.91846409180008	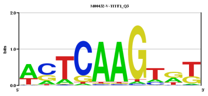	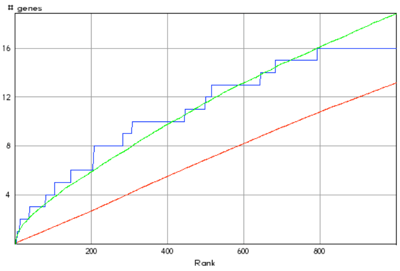	link	link	M00432-V-TITF1_Q3
MA0001	2.90359380645754	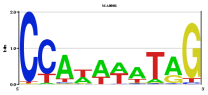	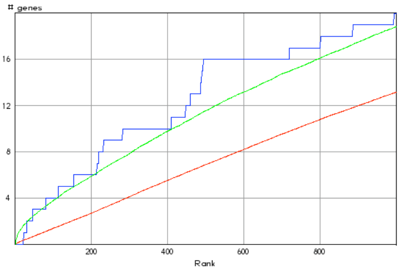	link	link	MA0001
GGGGAWTCCCY-disordered	2.88868629887385	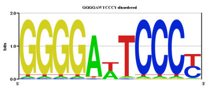	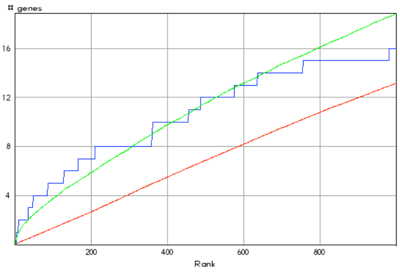	link	link	GGGGAWTCCCY-disordered
CGTGNGAA	2.86263073006338	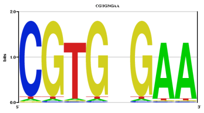	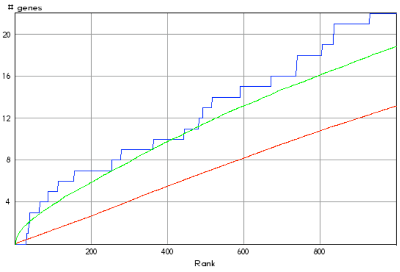	link	link	CGTGNGAA
M00087-V-IK2_01	2.83108488068215	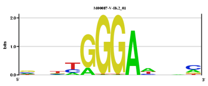	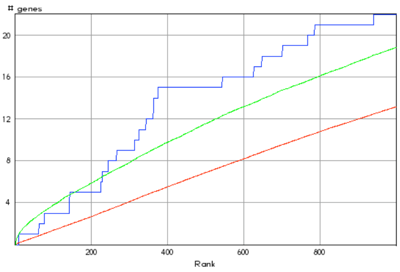	link	link	M00087-V-IK2_01

SelexConsensus_Rel	2.7863251356899	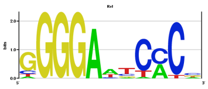	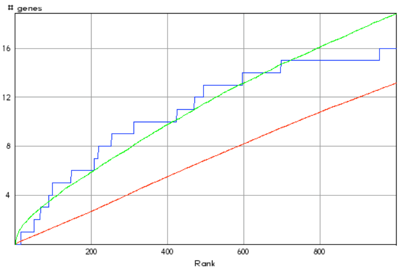	link	link	SelexConsensus_Rel
GGGGAWYCMC-Relish	2.76399179099523	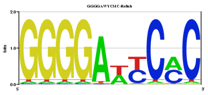	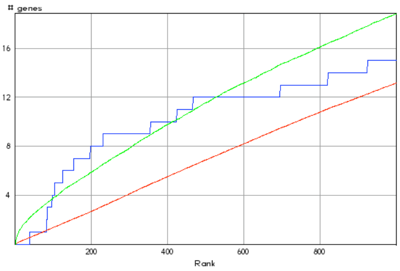	link	link	GGGGAWYCMC-Relish
M00276-F-MAT1MC_02	2.68960314204137	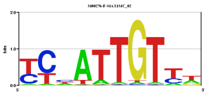	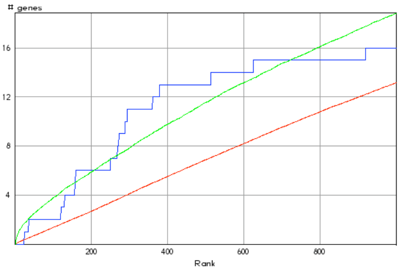	link	link	M00276-F-MAT1MC_02
AACATGTG	2.66345451762802	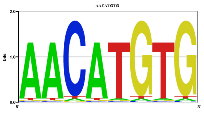	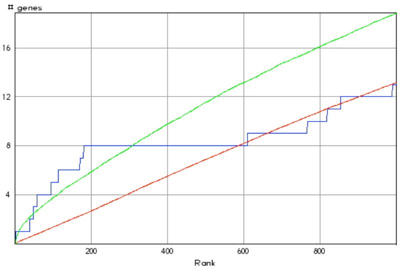	link	link	AACATGTG
