# Supplementary material for: Robust Target Gene Discovery through Transcriptome Perturbations and Genome-Wide Enhancer Predictions in Drosophila Uncovers a Regulatory Basis for Sensory Specification
Source: PLoS Biol. 2010 Jul 27;8(7):e1000435. doi: 10.1371/journal.pbio.1000435 (PMC2910651; doi:10.1371/journal.pbio.1000435)
Supplement: Table S3 — Upregulated genes in Ato GOF. SetA consists of 204 genes that are significantly upregulated in six Ato GOF samples versus eight control samples. (0.09 MB PDF) [file pbio.1000435.s014.pdf]

Supplementary Table S3

Up-regulated genes in Ato GOF. SetA consists of 204 genes that are significantly upregulated in six Ato GOF samples versus eight control samples.

CG32006	FBgn0052006	CG32006
ato	FBgn0010433	CG7508
sNPF	FBgn0032840	CG13968
sens	FBgn0002573	CG32120
CG6044	FBgn0034725	CG6044
ventrally-expressed-protein-D	FBgn0053200	CG33200
boss	FBgn0000206	CG8285
CG15589	FBgn0037409	CG15589
CG9335	FBgn0032895	CG9335
inaC	FBgn0004784	CG6518
Nplp1	FBgn0035092	CG3441
Lim3	FBgn0002023	CG10699
Fsh	FBgn0016650	CG7665
CG14321	FBgn0038540	CG14321
CG1625	FBgn0033447	CG1625
sca	FBgn0003326	CG17579
CG32150	FBgn0052150	CG32150
CG33458	FBgn0053458	CG33458
CG30492	FBgn0050492	CG30492
Rph	FBgn0030230	CG11556
nvy	FBgn0005636	CG3385
phyl	FBgn0013725	CG10108
Actbeta	FBgn0024913	CG11062
dpr9	FBgn0038282	CG33485
CG6026	FBgn0038676	CG6026
king-tubby	FBgn0015721	CG9398
Mob2	FBgn0259481	CG11711
CG11637	FBgn0036822	CG11637
Cad88C	FBgn0038247	CG3389
CG15097	FBgn0034396	CG15097
beat-IIIc	FBgn0032629	CG15138
CG14042	FBgn0260451	CG14042
SP555	FBgn0260470	CG14041
grim	FBgn0015946	CG4345
CG6520	FBgn0034224	CG6520
Ank2	FBgn0085445	CG34416
CG13532	FBgn0034788	CG13532
SP2353	FBgn0034070	CG8403
CG3703	FBgn0040348	CG3703
scrt	FBgn0004880	CG1130
beat-IIIa	FBgn0032627	CG12621
CG2556	FBgn0030396	CG2556
CG14234	FBgn0031065	CG14234
CG14186	FBgn0036935	CG14186
comm2	FBgn0041160	CG7554
CG34007	FBgn0054007	CG34007
Pde8	FBgn0034886	CG5411

peb	FBgn0003053	CG12212
comm	FBgn0010105	CG17943
CG9363	FBgn0037697	CG9363
Fhos	FBgn0052030	CG32030
mira	FBgn0021776	CG12249
CG6114	FBgn0036544	CG6114
Pka-R2	FBgn0022382	CG15862
CG31871	FBgn0051871	CG31871
Aplip1	FBgn0040281	CG1200
CG3603	FBgn0029648	CG3603
CG17959	FBgn0029647	CG17959
unc	FBgn0003950	CG1501
CG8401	FBgn0034069	CG8401
CG5375	FBgn0032221	CG5375
neur	FBgn0002932	CG11988
CG32667	FBgn0052667	CG32667
X11Lbeta	FBgn0052677	CG32677
amon	FBgn0023179	CG6438
CG4927	FBgn0034139	CG4927
E(spl)	FBgn0000591	CG8365
HLHmgamma	FBgn0002735	CG8333
HLHmdelta	FBgn0002734	CG8328
CG6495	FBgn0027550	CG6495
unc-104	FBgn0034155	CG8566
Ank2	FBgn0085445	CG34416
Fas2	FBgn0000635	CG3665
f	FBgn0000630	CG5424
spdo	FBgn0260440	CG31020
CG3556	FBgn0029708	CG3556
CG42327	FBgn0259227	CG42327
rho	FBgn0004635	CG1004
osm-6	FBgn0031829	CG9595
CG12972	FBgn0037076	CG12972
CG6024	FBgn0036202	CG6024
CG4950	FBgn0036587	CG4950
qua	FBgn0003187	CG6433
CG42327	FBgn0259227	CG42327
SytIV	FBgn0028400	CG10047
CG32039	FBgn0052039	CG32039
spir	FBgn0003475	CG10076
CG1309	FBgn0035519	CG1309
CG1268	FBgn0035521	CG1268
CG34451	FBgn0085480	CG34451
CG34452	FBgn0085481	CG34452
Victoria	FBgn0053117	CG33117
Cdk5alpha	FBgn0027491	CG5387
RhoGAP100F	FBgn0039883	CG1976
CG11638	FBgn0040351	CG11638
Dscam	FBgn0033159	CG17800
PIP5K59B	FBgn0034789	CG3682
CG13409	FBgn0038926	CG13409
CG17264	FBgn0031490	CG17264
CG5337	FBgn0032249	CG5337

Ac13E	FBgn0022710	CG9210
a	FBgn0000008	CG6741
m4	FBgn0002629	CG6099
CG14864	FBgn0038311	CG14864
Rapgap1	FBgn0085403	CG34374
CG6329	FBgn0033872	CG6329
betaTub60D	FBgn0003888	CG3401
Ror	FBgn0010407	CG4926
Nrt	FBgn0004108	CG9704
CG10098	FBgn0037472	CG10098
CG31755	FBgn0051755	CG31755
lola	FBgn0005630	CG12052
CG9279	FBgn0036882	CG9279
dap	FBgn0010316	CG1772
CG7646	FBgn0036926	CG7646
orb	FBgn0004882	CG10868
Teh1	FBgn0037766	CG12806
CG15863	FBgn0033467	CG15863
Spase22-23	FBgn0039172	CG5677
CG9801	FBgn0037623	CG9801
CG31176	FBgn0051176	CG31176
CG30456	FBgn0050456	CG30456
MYPT-75D	FBgn0036801	CG6896
CG31875	FBgn0051875	CG31875
CG31030	FBgn0051030	CG31030
CG31028	FBgn0051028	CG31028
Spn	FBgn0010905	CG16757
CG32295	FBgn0260480	CG32295
CG42366	FBgn0259712	CG42366
CG42367	FBgn0259713	CG42367
CG42366	FBgn0259712	CG42366
CG42367	FBgn0259713	CG42367
CG9646	FBgn0034184	CG9646
seq	FBgn0028991	CG32904
CG30343	FBgn0050343	CG30343
hts	FBgn0004873	CG9325
CG17724	FBgn0033802	CG17724
CG10566	FBgn0037050	CG10566
Gbeta5	FBgn0030011	CG10763
RpS19b	FBgn0039129	CG5338
CG8965	FBgn0031745	CG8965
vkg	FBgn0016075	CG16858
CG6860	FBgn0032633	CG6860
Src64B	FBgn0003501	CG7524
PpD3	FBgn0005777	CG8402
OstStt3	FBgn0011336	CG7748
veli	FBgn0039269	CG7662
gl	FBgn0004618	CG7672
CG10721	FBgn0032846	CG10721
CG31714	FBgn0032180	CG31714
CG11577	FBgn0036847	CG11577
CG10424	FBgn0036848	CG10424
san	FBgn0024188	CG12352

CG31917	FBgn0031668	CG31917
CG8892	FBgn0031664	CG8892
CG34015	FBgn0054015	CG34015
CG8228	FBgn0037711	CG8228
SRPK	FBgn0026370	CG8174
CG13025	FBgn0036660	CG13025
ari-2	FBgn0025186	CG5709
loqs	FBgn0032515	CG6866
mRpL52	FBgn0033208	CG1577
U2A	FBgn0033210	CG1406
CG1407	FBgn0033474	CG1407
CG41128	FBgn0069923	CG41128
DAAM	FBgn0025641	CG14622
CG12848	FBgn0040666	CG12848
CG5902	FBgn0039136	CG5902
siz	FBgn0026179	CG32434
CG10338	FBgn0032700	CG10338
CG17257	FBgn0031495	CG17257
CG17219	FBgn0031494	CG17219
CG4042	FBgn0037018	CG4042
cup	FBgn0000392	CG11181
CG10859	FBgn0032520	CG10859
levy	FBgn0034877	CG17280
CG11103	FBgn0030522	CG11103
CG5934	FBgn0039505	CG5934
CG7770	FBgn0036918	CG7770
Cbp20	FBgn0022943	CG12357
Rab21	FBgn0039966	CG17515
CG2218	FBgn0039767	CG2218
CG15536	FBgn0039766	CG15536
Ubi-p63E	FBgn0003943	CG11624
CG31005	FBgn0051005	CG31005
CG16957	FBgn0032519	CG16957
hkl	FBgn0086441	CG10473
CG10470	FBgn0032746	CG10470
CG9257	FBgn0032916	CG9257
ric8a	FBgn0028292	CG15797
CG5447	FBgn0039427	CG5447
Hex-t2	FBgn0042710	CG32849
CG3825	FBgn0034948	CG3825
CG4169	FBgn0250814	CG4169
Rpn12	FBgn0028693	CG4157
Rho1	FBgn0014020	CG8416
Klc	FBgn0010235	CG5433
CG13775	FBgn0031874	CG13775
Arf102F	FBgn0013749	CG11027
CG4074	FBgn0037017	CG4074
CG7394	FBgn0036173	CG7394
cni	FBgn0000339	CG5855
O-fut2	FBgn0027791	CG14789
CG8315	FBgn0034058	CG8315
CG9883	FBgn0031435	CG9883
p16-ARC	FBgn0031437	CG9881

Mo25	FBgn0017572	CG4083
CG12162	FBgn0037329	CG12162
CG11388	FBgn0034959	CG11388
ATPCL	FBgn0020236	CG8322
