# Supplementary material for: Robust Target Gene Discovery through Transcriptome Perturbations and Genome-Wide Enhancer Predictions in Drosophila Uncovers a Regulatory Basis for Sensory Specification
Source: PLoS Biol. 2010 Jul 27;8(7):e1000435. doi: 10.1371/journal.pbio.1000435 (PMC2910651; doi:10.1371/journal.pbio.1000435)
Supplement: Table S4 — cis TargetX results for 204 Ato-upregulated genes. The best motif out of 1,981 tested motifs is RACASCTGY. (2.57 MB PDF) [file pbio.1000435.s015.pdf]

cisTargetX AUC results

You can select one or more motifs and [proceed] to Cluster-Buster enhancer predictions across 12 species
Download target gene lists for all significant motifs

Motif	Z-score	Logo	ROC	Candidate targets	All genes in top 1000	Select
RACASCTGY	3.86190407121412	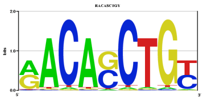	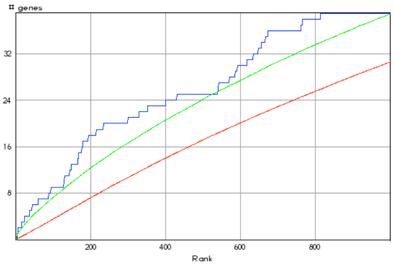	link	link	RACASCTGY
M00184-V-MYOD_Q6	3.74919781523385	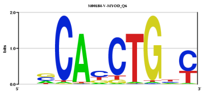	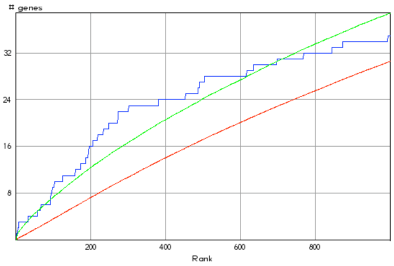	link	link	M00184-V-MYOD_Q6
M00693-V-E12_Q6	3.6364675791991	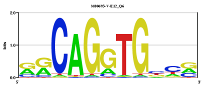	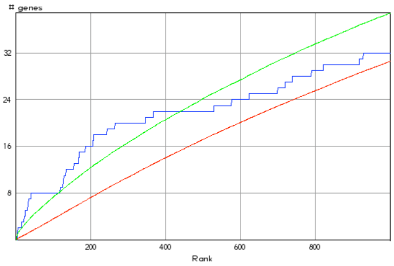	link	link	M00693-V-E12_Q6
M00001-V-MYOD_01	3.37870597376676	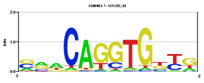	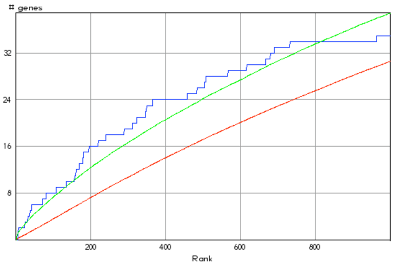	link	link	M00001-V-MYOD_01
M00973-V-E2A_Q6	3.27801372507289	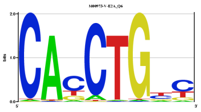	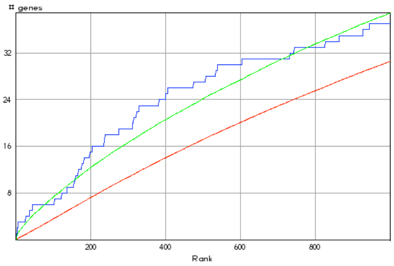	link	link	M00973-V-E2A_Q6

RRCAGGTGB-escargot	3.24439368871453	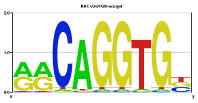	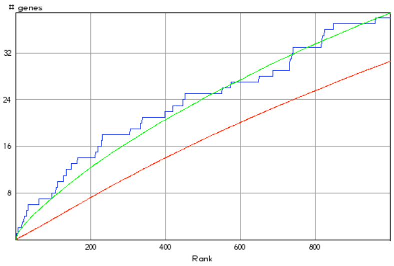	link	link	RRCAGGTGB-escargot
M00234-I-SUH_01	3.1832805199133	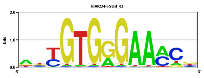	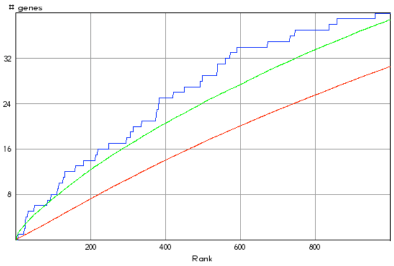	link	link	M00234-I-SUH_01
CGTGNGAA	3.06333228748493	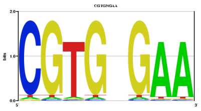	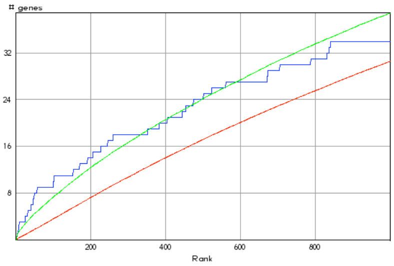	link	link	CGTGNGAA
M00712-V-MYOGENIN_Q6	2.99980912321009	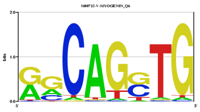	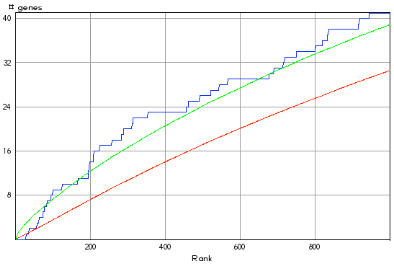	link	link	M00712-V-MYOGENIN_Q6
MA0091	2.99383808964858	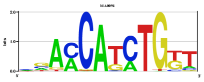	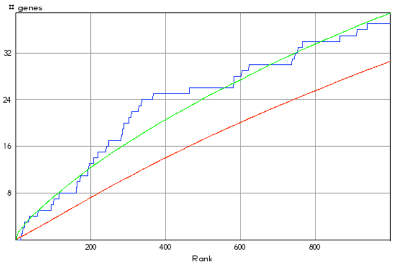	link	link	MA0091
PF0074	2.91226993438966	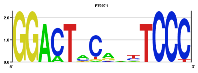	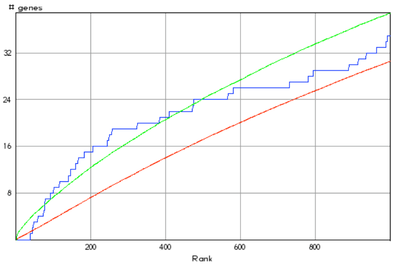	link	link	PF0074
M00002-V-E47_01	2.88828987992577	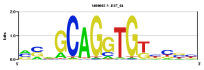	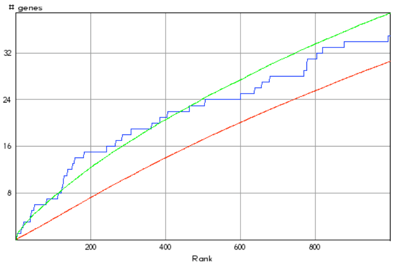	link	link	M00002-V-E47_01

M00804-V-E2A_Q2	2.76601558221441	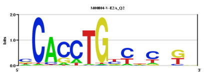	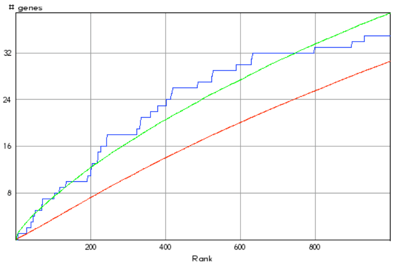	link	link	M00804-V-E2A_Q2
CAGCTGC	2.70965046419704	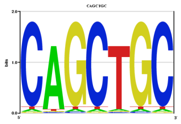	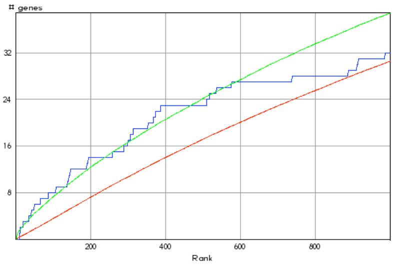	link	link	CAGCTGC
sna	2.6820494215091	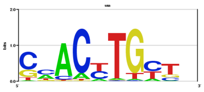	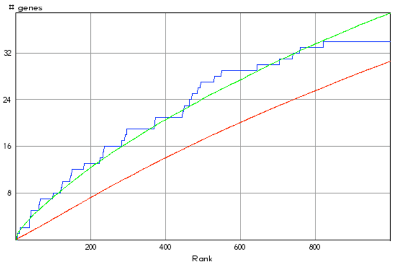	link	link	sna
atopwm3	2.67363242239228	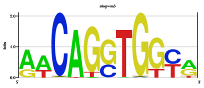	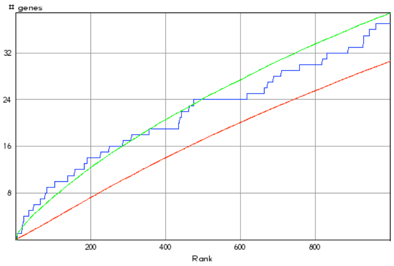	link	link	atopwm3
CATGGAGG	2.61251925359106	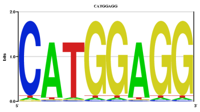	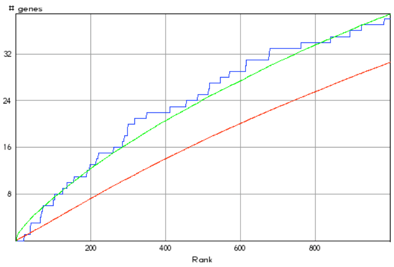	link	link	CATGGAGG
GCAGSTGK-scute	2.6029272318055	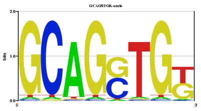	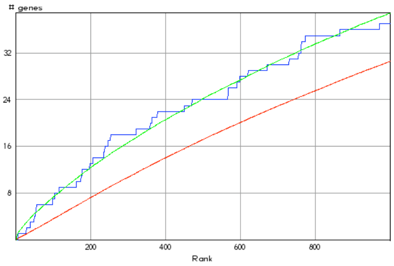	link	link	GCAGSTGK-scute

M00239-V-T3R_01	2.54056710017216	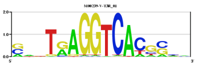	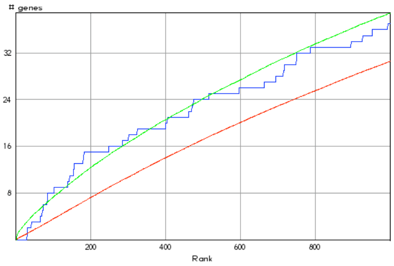	link	link	M00239-V-T3R_01
RBYGTGRGAAMCB-Suppressor	2.53215010105534	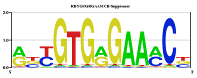	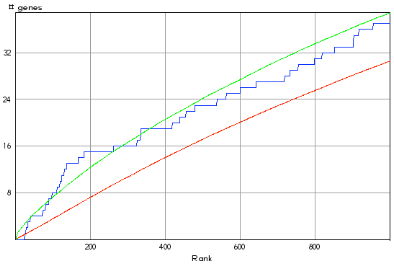	link	link	RBYGTGRGAAMCB-Suppressor
CAGSTG-asense	2.49263097129885	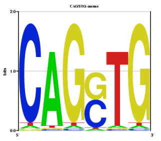	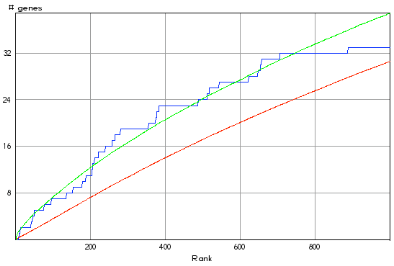	link	link	CAGSTG-asense
MA0085	2.44585787506704	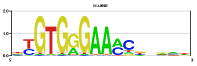	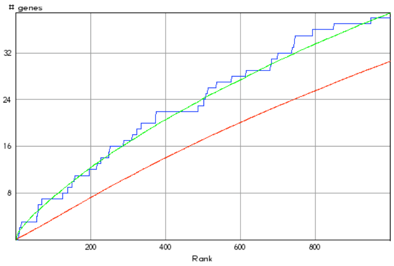	link	link	MA0085
AACAGCTG	2.42190180065761	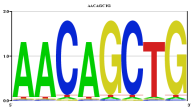	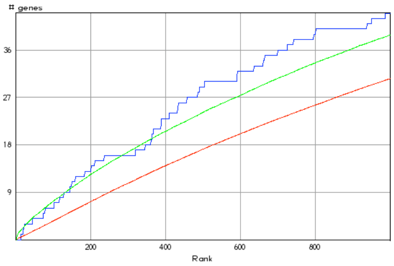	link	link	AACAGCTG
M00261-V-OLF1_01	2.37150771620174	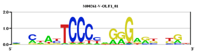	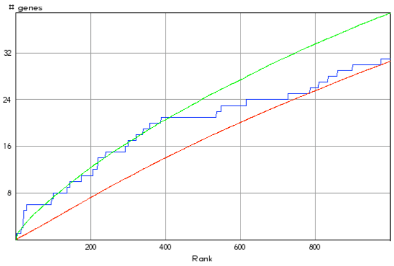	link	link	M00261-V-OLF1_01
PF0035	2.36666374520005	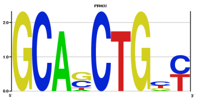	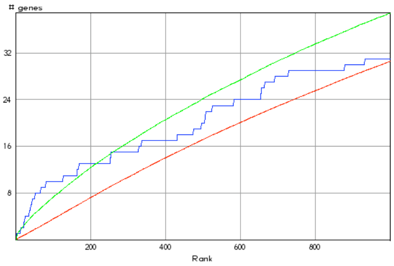	link	link	PF0035

M00086-V-IK1_01	2.36551270258577	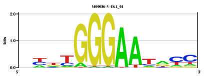	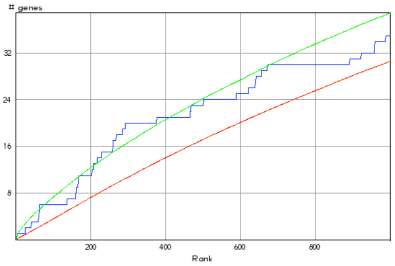	link	link	M00086-V-IK1_01
atopwm5	2.35469769802256	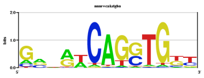	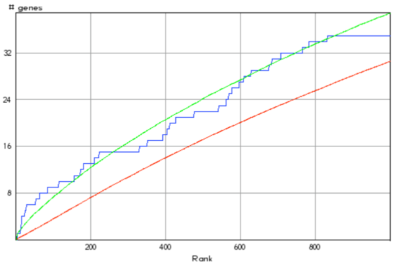	link	link	atopwm5
CAGNNGCA	2.34871467443382	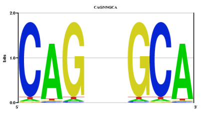	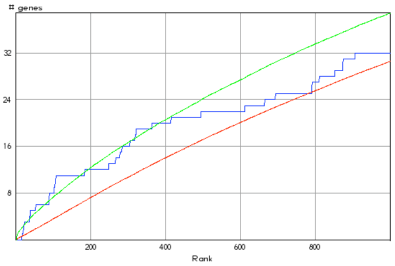	link	link	CAGNNGCA
M00929-V-MYOD_Q6_01	2.337971610034	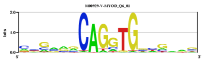	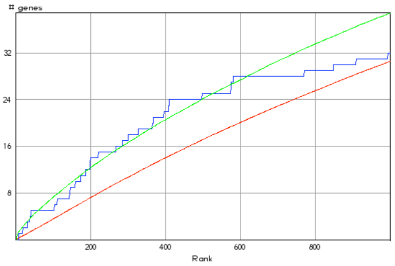	link	link	M00929-V-MYOD_Q6_01
M00277-V-LMO2COM_01	2.30674957912202	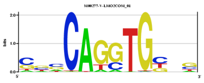	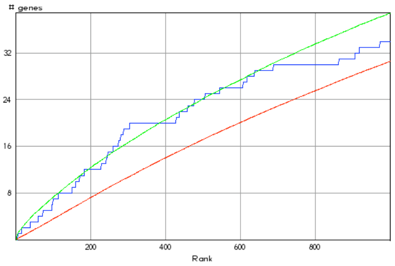	link	link	M00277-V-LMO2COM_01
M00066-V-TAL1ALPHAE47_01	2.3031765510069	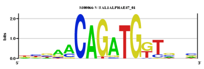	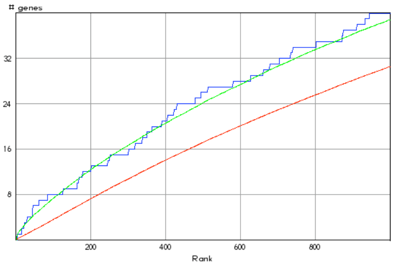	link	link	M00066-V-TAL1ALPHAE47_01

M00499-V-STAT5A_04	2.29954357275561	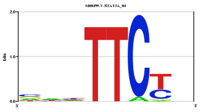	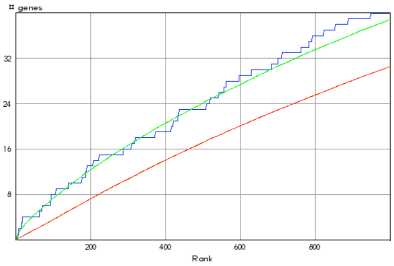	link	link	M00499-V-STAT5A_04
M00125-F-MCM1_01	2.28996354099729	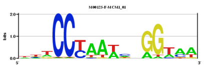	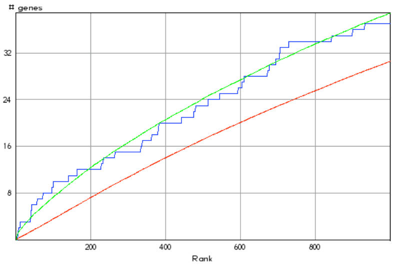	link	link	M00125-F-MCM1_01
atopwm7	2.28755354552367	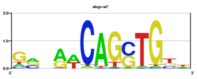	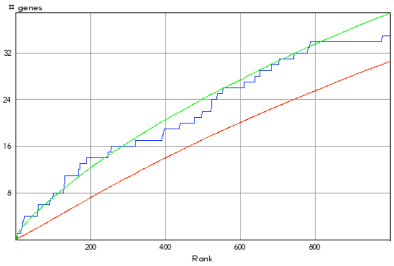	link	link	atopwm7
CTGCAGC	2.28394454732685	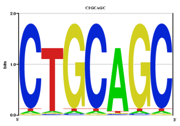	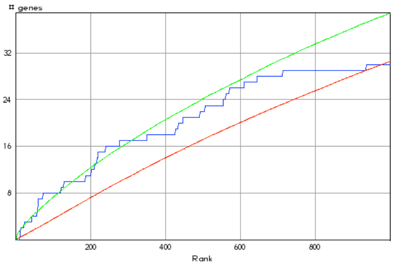	link	link	CTGCAGC
M00927-V-AP4_Q6_01	2.28279350471259	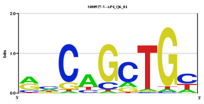	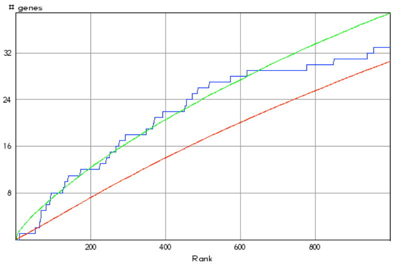	link	link	M00927-V-AP4_Q6_01
M00116-V-CEBPA_01	2.2755874983462	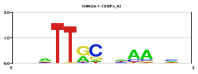	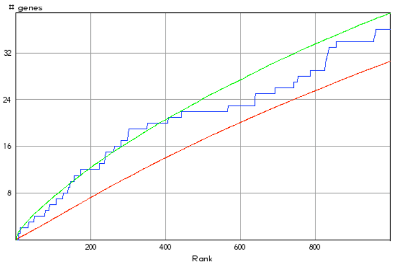	link	link	M00116-V-CEBPA_01
M00664-F-STE12_Q4	2.27321347295426	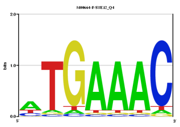	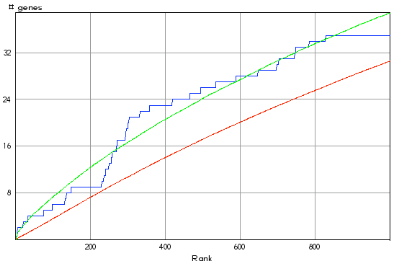	link	link	M00664-F-STE12_Q4

M00964-V-PXR_Q2	2.24080442934632	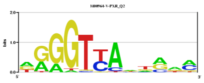	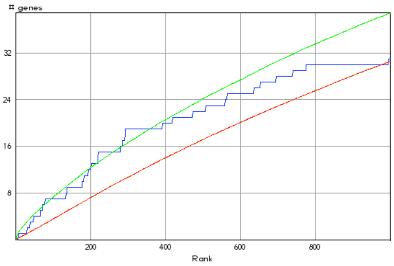	[link]	[link]	M00964-V-PXR_Q2
M00494-V-STAT6_01	2.22880241208714	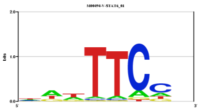	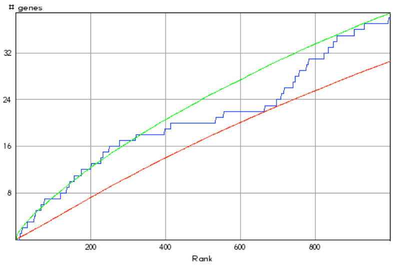	[link]	[link]	M00494-V-STAT6_01
ACACCTG	2.20489429778665	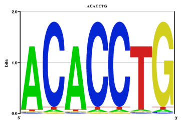	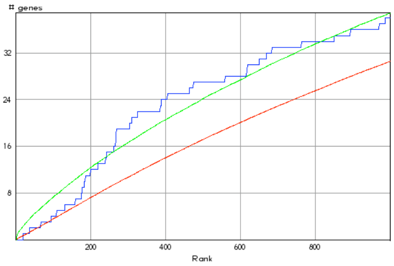	[link]	[link]	ACACCTG
M00993-V-TAL1_Q6	2.19764033131132	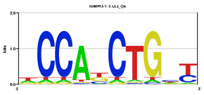	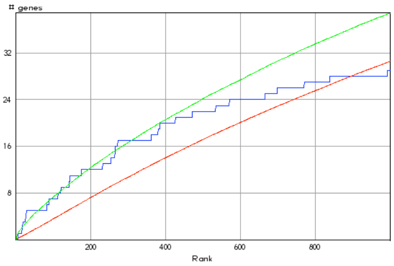	[link]	[link]	M00993-V-TAL1_Q6
PF0092	2.1748472895434	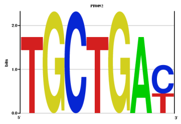	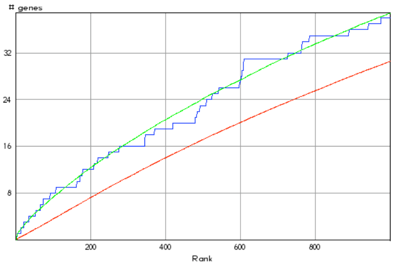	[link]	[link]	PF0092
M00641-V-HSF_Q6	2.17363629679297	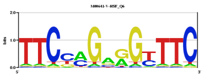	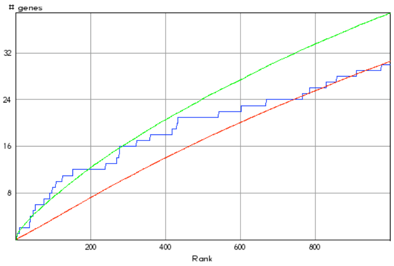	[link]	[link]	M00641-V-HSF_Q6

Espl	2.17363629679297	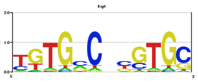	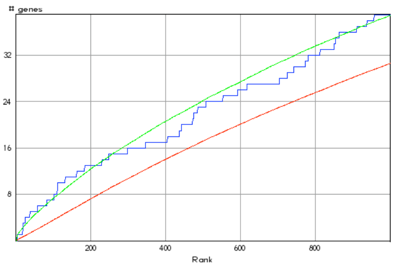	link	link	Espl
GGGGAMWWCCM-schnurri	2.1652792478123	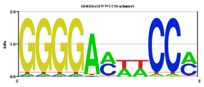	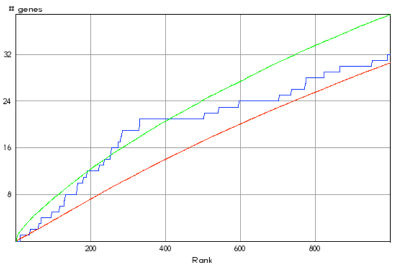	link	link	GGGGAMWWCCM-schnurri
M01034-V-EBOX_Q6_01	2.16526725778507	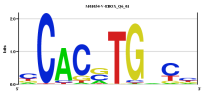	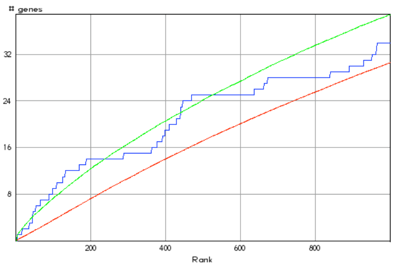	link	link	M01034-V-EBOX_Q6_01
PF0155	2.14851718974205	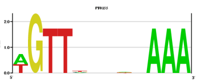	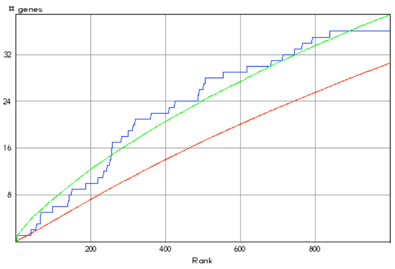	link	link	PF0155
M00726-V-USF2_Q6	2.13530417973244	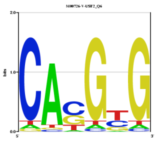	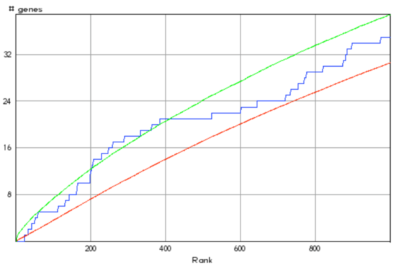	link	link	M00726-V-USF2_Q6
CAGCTGG	2.13528019967798	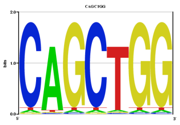	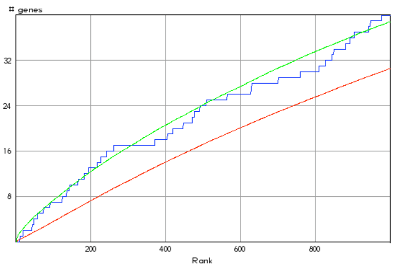	link	link	CAGCTGG
M00796-V-USF_Q6_01	2.129285186062	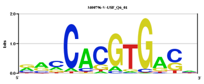	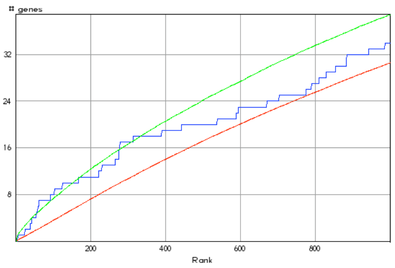	link	link	M00796-V-USF_Q6_01

M00122-V-USF_02	2.11849416155326	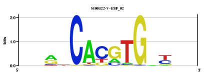 .	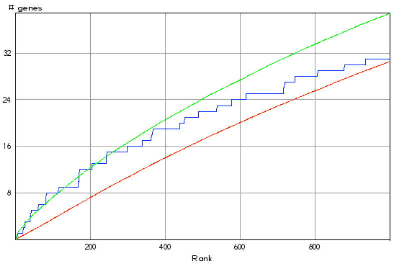	link	link	M00122-V-USF_02
M00146-V-HSF1_01	2.11249914793729	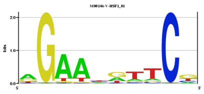 .	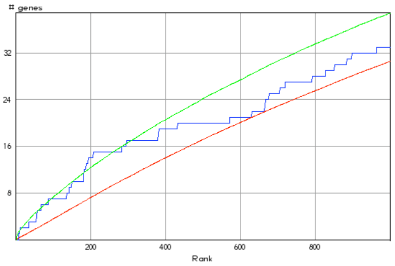	link	link	M00146-V-HSF1_01
MA0054	2.08494606535828	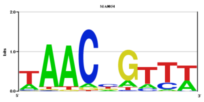 .	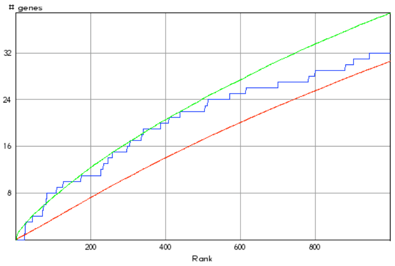	link	link	MA0054
M00461-I-OVO_01	2.06817201726079	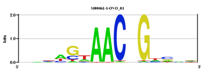 .	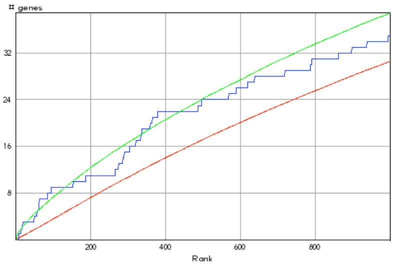	link	link	M00461-I-OVO_01
MA0027	2.06095402086716	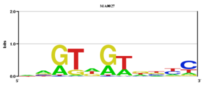 .	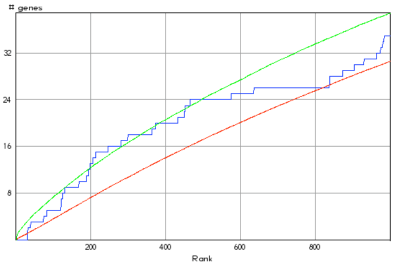	link	link	MA0027
ATGTGGA	2.04296898001924	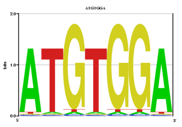 .	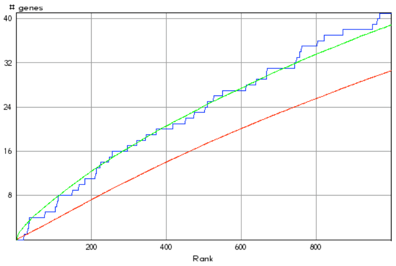	link	link	ATGTGGA

AGCAGCT	2.03095497273283	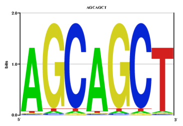	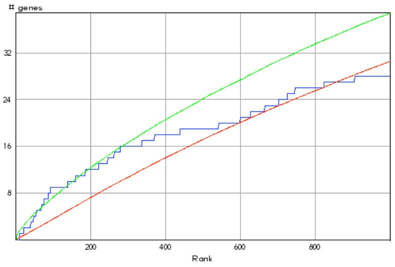	link	link	AGCAGCT
M00486-V-PAX2_02	2.02619493192175	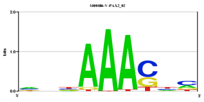	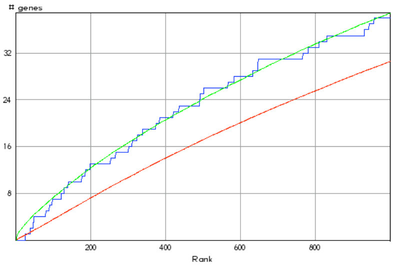	link	link	M00486-V-PAX2_02
M00498-V-STAT4_01	2.02256195367047	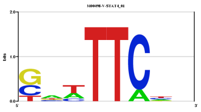	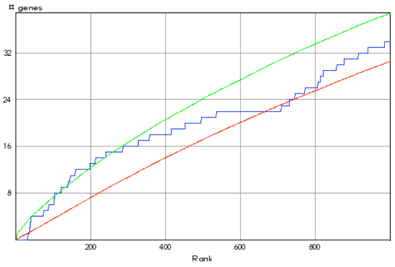	link	link	M00498-V-STAT4_01
AAGCTGG	2.01302988202108	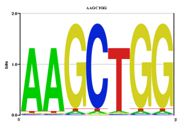	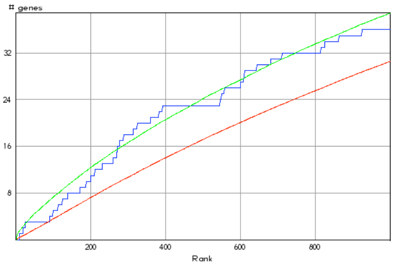	link	link	AAGCTGG
M00997-V-DEC_Q1	2.01296993188492	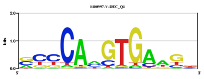	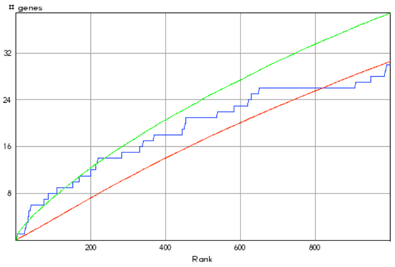	link	link	M00997-V-DEC_Q1
M01112-V-RBPJK_01	2.00341388018105	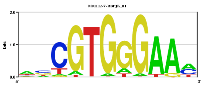	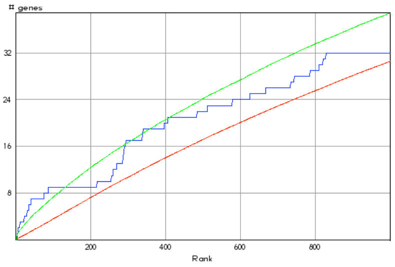	link	link	M01112-V-RBPJK_01
M00690-V-AP3_Q6	2.00221487745786	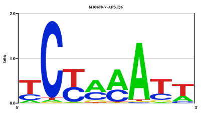	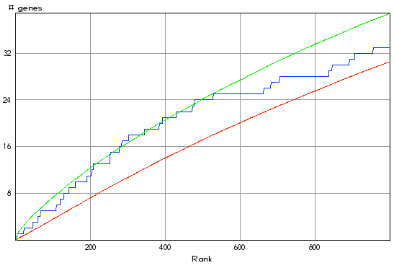	link	link	M00690-V-AP3_Q6

M00747-V-IRF1_Q6	1.99502086111869	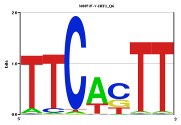	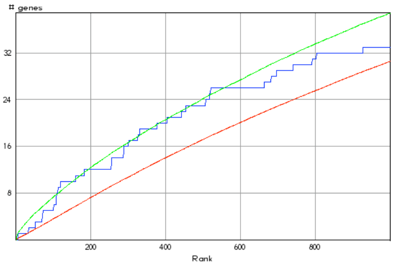	link	link	M00747-V-IRF1_Q6
M00322-V-MYCMAX_B	1.99374991823211	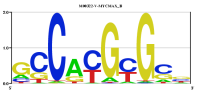	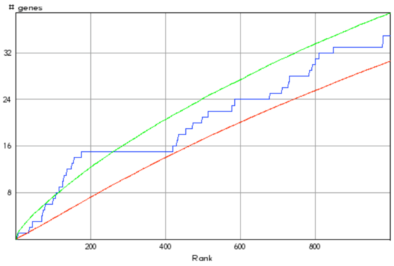	link	link	M00322-V-MYCMAX_B
PF0045	1.96984180393162	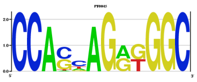	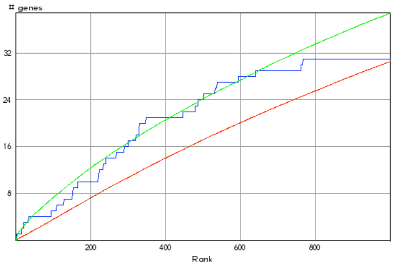	link	link	PF0045
CAGCTGCA	1.96864280120842	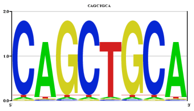	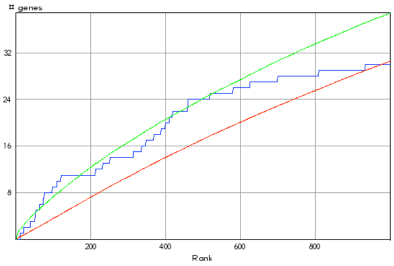	link	link	CAGCTGCA
M00175-V-AP4_Q5	1.96383480028841	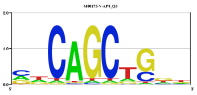	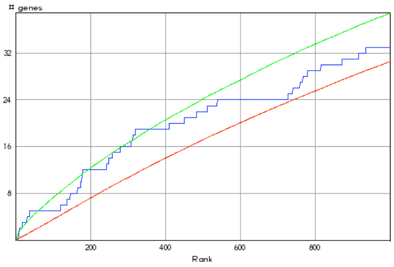	link	link	M00175-V-AP4_Q5
