# Supplementary material for: Robust Target Gene Discovery through Transcriptome Perturbations and Genome-Wide Enhancer Predictions in Drosophila Uncovers a Regulatory Basis for Sensory Specification
Source: PLoS Biol. 2010 Jul 27;8(7):e1000435. doi: 10.1371/journal.pbio.1000435 (PMC2910651; doi:10.1371/journal.pbio.1000435)
Supplement: Table S5 — Count matrix representing the Atonal binding site. We constructed a “phyloPWM” [28],[45] for Atonal in TOUCAN [60] using known Ato [42], Brd [31], TakR86C [66], Math1 [67], and ATH5 binding sites [68], including aligned and conserved binding sites from other species, obtained from UCSC Genome Browser alignments. (0.05 MB PDF) [file pbio.1000435.s016.pdf]

# Supplementary Table S5

| A | C | G | T |
|---|---|---|---|
| 23 | 0 | 10 | 0 |
| 22 | 0 | 0 | 11 |
| 0 | 33 | 0 | 0 |
| 33 | 0 | 0 | 0 |
| 0 | 0 | 30 | 3 |
| 0 | 11 | 22 | 0 |
| 0 | 0 | 0 | 33 |
| 0 | 0 | 33 | 0 |
| 0 | 0 | 26 | 7 |
| 0 | 17 | 0 | 16 |
| 17 | 0 | 13 | 3 |
