# Supplementary material for: Robust Target Gene Discovery through Transcriptome Perturbations and Genome-Wide Enhancer Predictions in Drosophila Uncovers a Regulatory Basis for Sensory Specification
Source: PLoS Biol. 2010 Jul 27;8(7):e1000435. doi: 10.1371/journal.pbio.1000435 (PMC2910651; doi:10.1371/journal.pbio.1000435)
Supplement: Table S6 — Direct Ato target gene predictions. Subset of Ato-misregulated genes (GOF and LOF) obtained from various cisTargetX analyses (see text for details). (0.07 MB PDF) [file pbio.1000435.s017.pdf]

# Supplementary Table S6

| | | |
|---|---|---|
| a | FBgn0000008 | CG6741 |
| amon | FBgn0023179 | CG6438 |
| ato | FBgn0010433 | CG7508 |
| beat-IIIa | FBgn0032627 | CG12621 |
| beat-IIIc | FBgn0032629 | CG15138 |
| betaTub60D | FBgn0003888 | CG3401 |
| br | FBgn0000210 | CG11491 |
| cenG1A | FBgn0028509 | CG31811 |
| CG15863 | FBgn0033467 | CG15863 |
| CG1625 | FBgn0033447 | CG1625 |
| CG17724 | FBgn0033802 | CG17724 |
| CG2556 | FBgn0030396 | CG2556 |
| CG30343 | FBgn0050343 | CG30343 |
| CG30492 | FBgn0050492 | CG30492 |
| CG31176 | FBgn0051176 | CG31176 |
| CG32150 | FBgn0052150 | CG32150 |
| CG32169 | FBgn0052169 | CG32169 |
| CG32206 | FBgn0052206 | CG32206 |
| CG32295 | FBgn0260480 | CG32295 |
| CG32667 | FBgn0052667 | CG32667 |
| CG6024 | FBgn0036202 | CG6024 |
| CG6495 | FBgn0027550 | CG6495 |
| CG6860 | FBgn0032633 | CG6860 |
| CG8965 | FBgn0031745 | CG8965 |
| CG9095 | FBgn0030617 | CG9095 |
| CG9801 | FBgn0037623 | CG9801 |
| DAAM | FBgn0025641 | CG14622 |
| dap | FBgn0010316 | CG1772 |
| dpr9 | FBgn0038282 | CG33485 |
| Dscam | FBgn0033159 | CG17800 |
| E(spl) | FBgn0000591 | CG8365 |
| Eip75B | FBgn0000568 | CG8127 |
| f | FBgn0000630 | CG5424 |
| Fas2 | FBgn0000635 | CG3665 |
| Fhos | FBgn0052030 | CG32030 |
| hts | FBgn0004873 | CG9325 |
| king-tubby | FBgn0015721 | CG9398 |
| Lim3 | FBgn0002023 | CG10699 |
| lola | FBgn0005630 | CG12052 |
| m4 | FBgn0002629 | CG6099 |
| mam | FBgn0002643 | CG8118 |
| Mmp2 | FBgn0033438 | CG1794 |
| Mob2 | FBgn0259481 | CG11711 |
| MYPT-75D | FBgn0036801 | CG6896 |
| nerfin-1 | FBgn0028999 | CG13906 |
| neur | FBgn0002932 | CG11988 |
| nmo | FBgn0011817 | CG7892 |
| nvy | FBgn0005636 | CG3385 |

| | | |
|---|---|---|
| Pde8 | FBgn0034886 | CG5411 |
| phyl | FBgn0013725 | CG10108 |
| Pka-R2 | FBgn0022382 | CG15862 |
| Rapgap1 | FBgn0085403 | CG34374 |
| rdx | FBgn0086364 | CG12537 |
| rho | FBgn0004635 | CG1004 |
| salm | FBgn0004579 | CG6464 |
| sano | FBgn0034408 | CG12758 |
| sca | FBgn0003326 | CG17579 |
| sca | FBgn0003326 | CG17579 |
| scrt | FBgn0004880 | CG1130 |
| sens | FBgn0002573 | CG32120 |
| seq | FBgn0028991 | CG32904 |
| shep | FBgn0052423 | CG32423 |
| siz | FBgn0026179 | CG32434 |
| sNPF | FBgn0032840 | CG13968 |
| spdo | FBgn0260440 | CG31020 |
| spir | FBgn0003475 | CG10076 |
| Spn | FBgn0010905 | CG16757 |
| Src64B | FBgn0003501 | CG7524 |
| SRPK | FBgn0026370 | CG8174 |
| Teh1 | FBgn0037766 | CG12806 |
| Traf4 | FBgn0026319 | CG3048 |
| Traf4 | FBgn0026319 | CG3048 |
| Victoria | FBgn0053117 | CG33117 |
| vkg | FBgn0016075 | CG16858 |
| vn | FBgn0003984 | CG10491 |
| X11Lbeta | FBgn0052677 | CG32677 |
