# Supplementary material for: Robust Target Gene Discovery through Transcriptome Perturbations and Genome-Wide Enhancer Predictions in Drosophila Uncovers a Regulatory Basis for Sensory Specification
Source: PLoS Biol. 2010 Jul 27;8(7):e1000435. doi: 10.1371/journal.pbio.1000435 (PMC2910651; doi:10.1371/journal.pbio.1000435)
Supplement: Table S7 — cis TargetX results for 315 Ato-downregulated genes in ato−/− eye-antennal discs. The best motifs are Su(H) motifs; E-box motifs are also significantly over-represented, such as RACASCTGY. (2.07 MB PDF) [file pbio.1000435.s018.pdf]

cisTargetX AUC results

Your list contained 315 genes
You can select one or more motifs and [proceed] to Cluster-Buster enhancer predictions across 12 species

Motif	Z-score	Logo	ROC	Candidate targets	All genes in top 500	Select
M00234-I-SUH_01	3.5506804320053	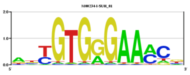	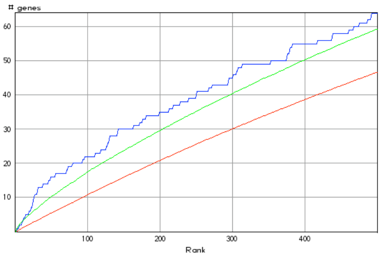	link	link	☐ M00234-I-SUH_01
M01112-V-RBPJK_01	3.28320178453461	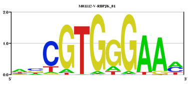	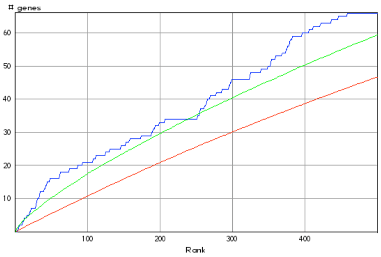	link	link	☐ M01112-V-RBPJK_01
MA0085	3.19442984662578	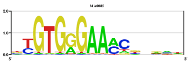	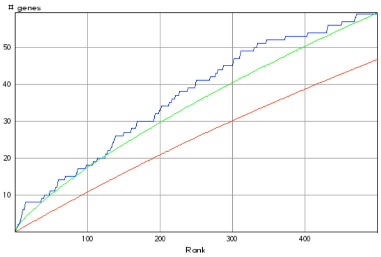	link	link	☐ MA0085
MAAMNNCAA	3.0478926301231	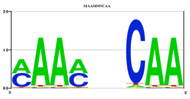	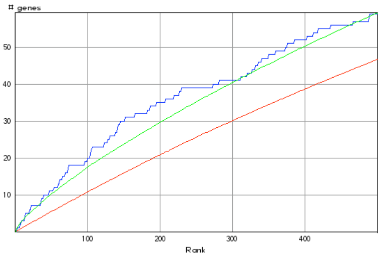	link	link	☐ MAAMNNCAA
M01111-V-RBPJK_Q4	3.03242626916847	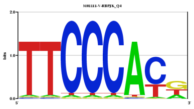	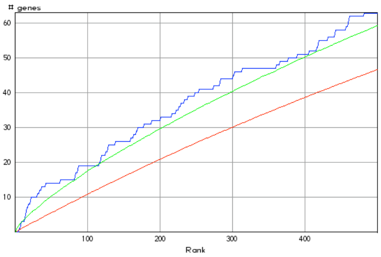	link	link	☐ M01111-V-RBPJK_Q4
M00086-V-IK1_01	2.97395522442935	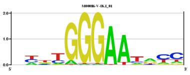	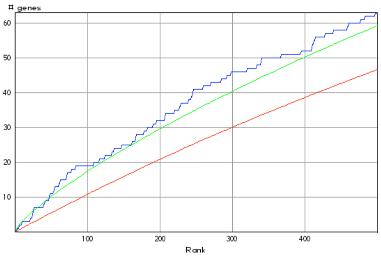	link	link	☐ M00086-V-IK1_01

M00028-I-HSF_01	2.92826190771064	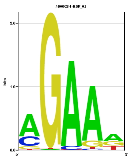	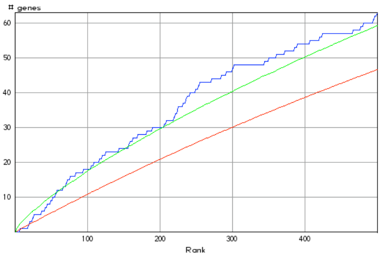	link	link	M00028-I-HSF_01
MA0045	2.77769846338883	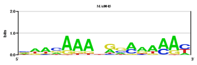	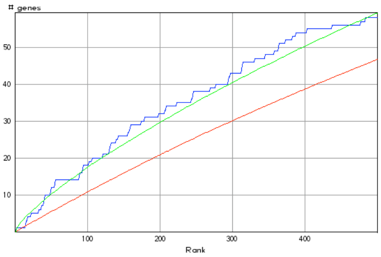	link	link	MA0045
AAANNNNNNNNNNAAT	2.74407710854518	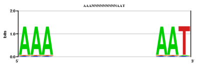	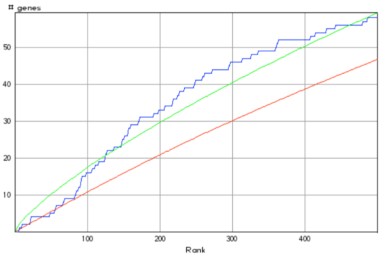	link	link	AAANNNNNNNNNNAAT
MA0120	2.73802096286062	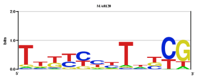	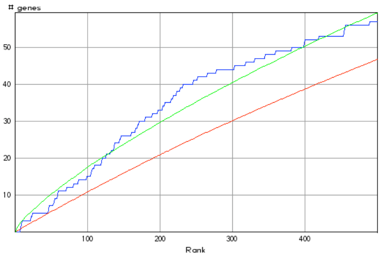	link	link	MA0120
M00624-V-DBP_Q6	2.72994834247533	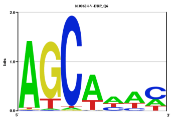	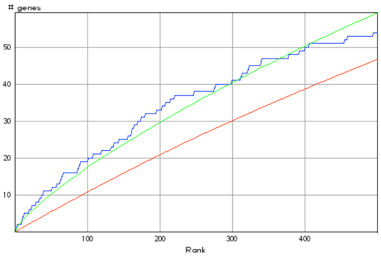	link	link	M00624-V-DBP_Q6
M01126-P-BPC1_Q2	2.67222339337552	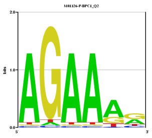	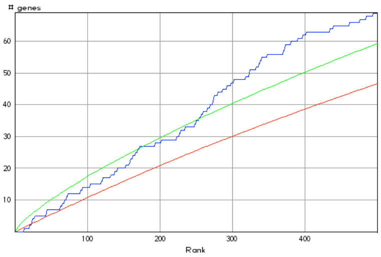	link	link	M01126-P-BPC1_Q2
HSWAACHGH-ovo	2.66474899381811	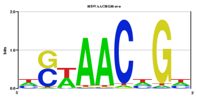	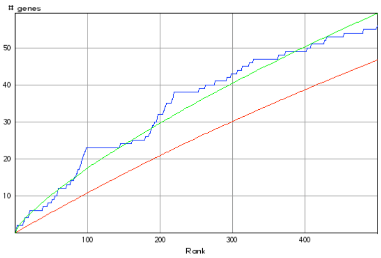	link	link	HSWAACHGH-ovo

sens	2.6432264871789	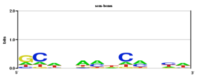	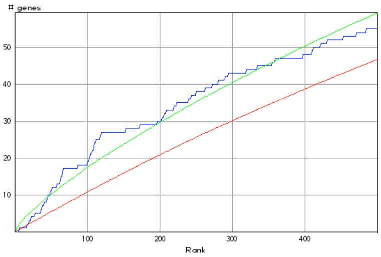	link	link	sens
Eip74EF	2.61434384788199	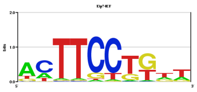	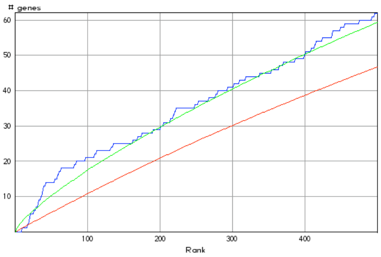	link	link	Eip74EF
M00712-V-MYOGENIN_Q6	2.55047537252726	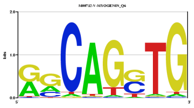	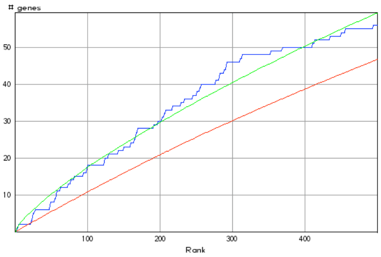	link	link	M00712-V-MYOGENIN_Q6
MAACAA	2.53971411920765	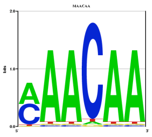	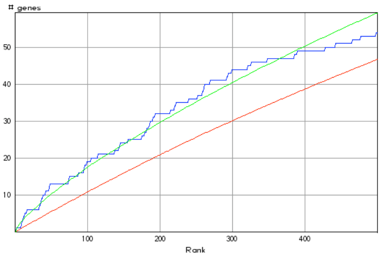	link	link	MAACAA
M01082-V-BRCA_01	2.52023497359851	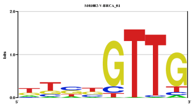	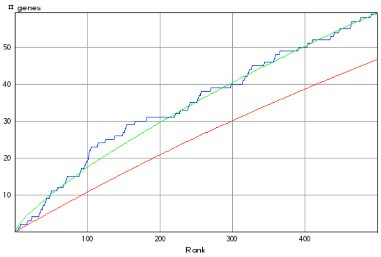	link	link	M01082-V-BRCA_01
RBYGTGRGAAMCB-Suppressor	2.5040830112456	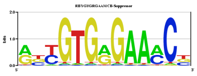	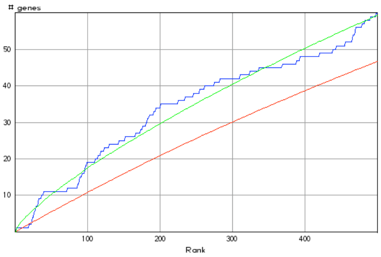	link	link	RBYGTGRGAAMCB-Suppressor
MF0009	2.46107160587885	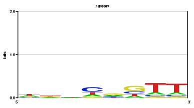	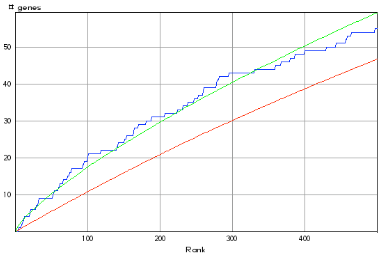	link	link	MF0009

M00456-V-FAC1_01	2.43286784639785	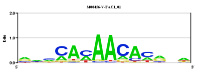	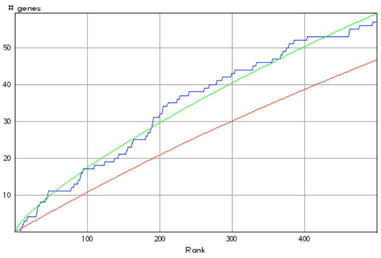	link	link	M00456-V-FAC1_01
AGCAACAA	2.4200901183775	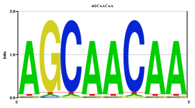	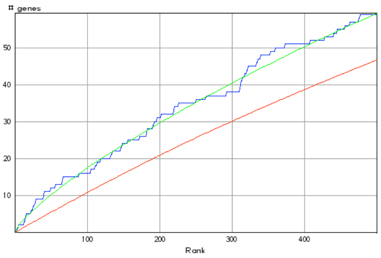	link	link	AGCAACAA
M01092-I-TCF_Q6	2.41268293464345	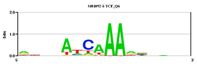	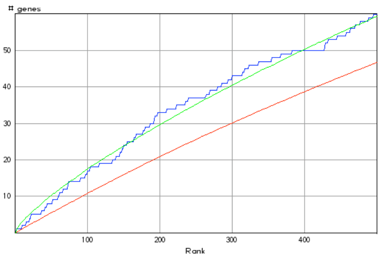	link	link	M01092-I-TCF_Q6
M00499-V-STAT5A_04	2.40733255510416	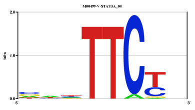	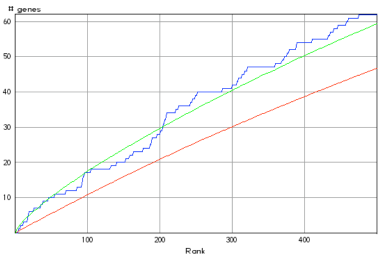	link	link	M00499-V-STAT5A_04
RACASCTGY	2.40596135230766	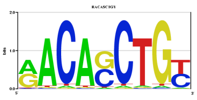	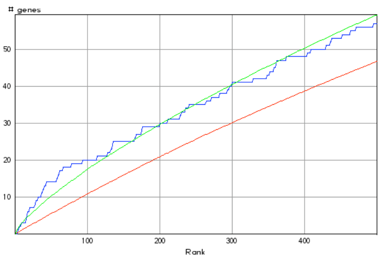	link	link	RACASCTGY
SelexConsensus_pan	2.37035713067495	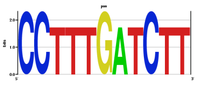	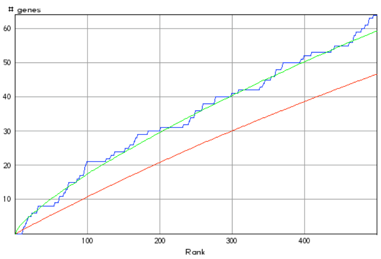	link	link	SelexConsensus_pan
CAGCTGG	2.36295666852324	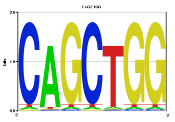	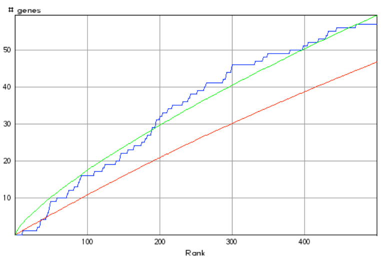	link	link	CAGCTGG

M00747-V-IRF1_Q6	2.35353973167079	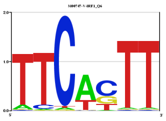	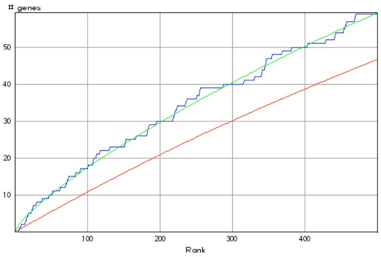	link	link	M00747-V-IRF1_Q6
CGTGNGAA	2.35216180729195	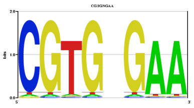	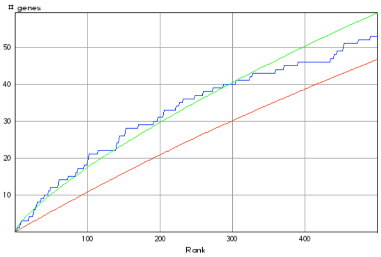	link	link	CGTGNGAA
CAGCTGC	2.35154342171706	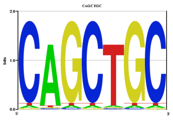	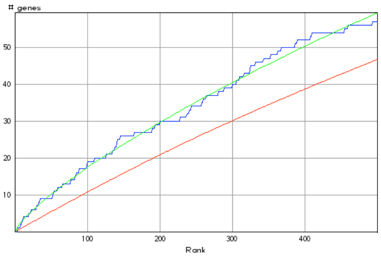	link	link	CAGCTGC
M01028-V-NRSF_Q4	2.30379330080357		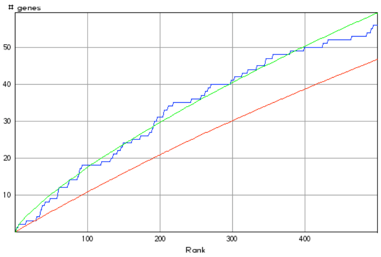	link	link	M01028-V-NRSF_Q4
GCAGSTGK-scute	2.29843619968194	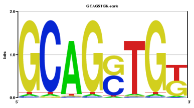	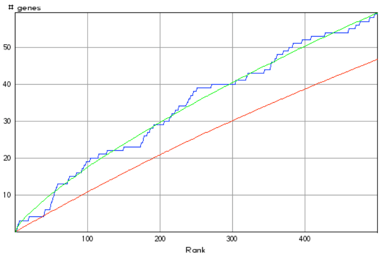	link	link	GCAGSTGK-scute
AACAGCTG	2.28769511110933	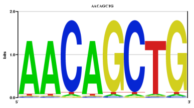	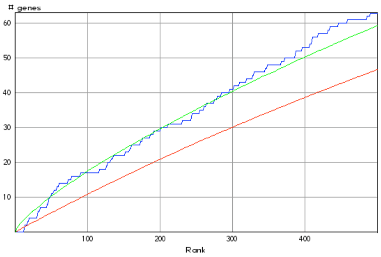	link	link	AACAGCTG
M00798-P-MYBAS1_01	2.28498631342801	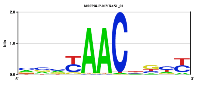	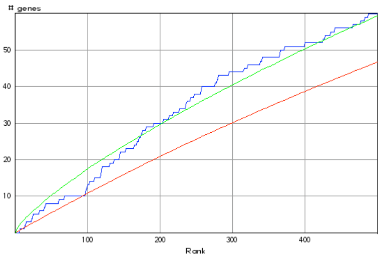	link	link	M00798-P-MYBAS1_01

M00401-P-ABF1_03	2.27624153480915	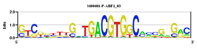	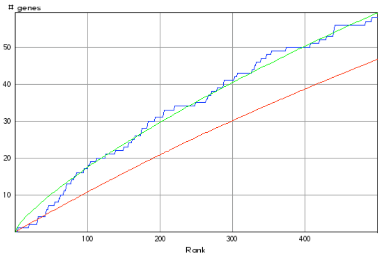	link	link	M00401-P-ABF1_03
M00281-V-RFX1_02	2.26345708520646	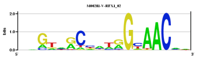	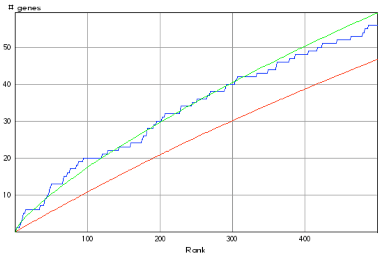	link	link	M00281-V-RFX1_02
M00353-P-DOF2_01	2.24602802220974	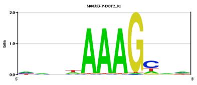	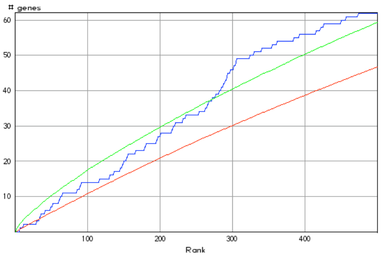	link	link	M00353-P-DOF2_01
M00927-V-AP4_Q6_01	2.22582966729068	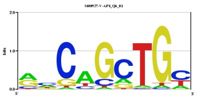	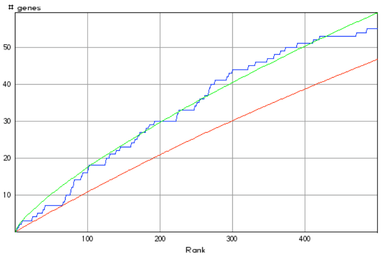	link	link	M00927-V-AP4_Q6_01
RKAAASA-broad	2.22381991417227	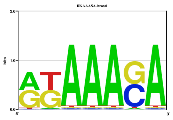	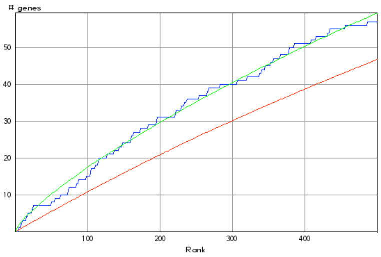	link	link	RKAAASA-broad
MA0100	2.21305866085266	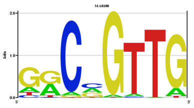	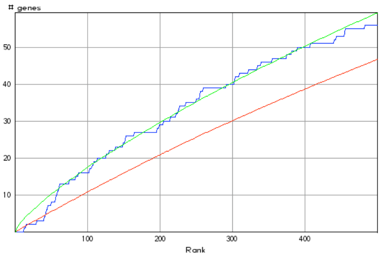	link	link	MA0100
TIFDMEM0000117	2.15725608430089	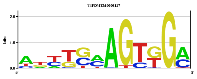	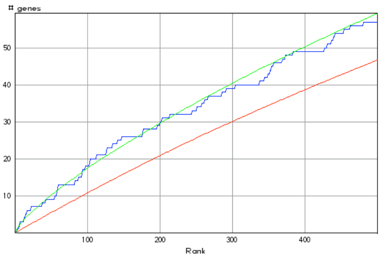	link	link	TIFDMEM0000117

M00658-V-PU1_Q6	2.14650827414595	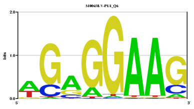 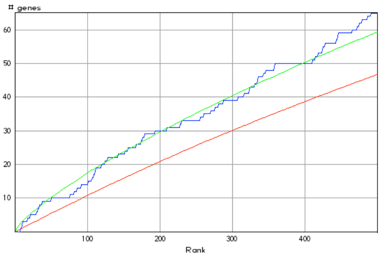	link	link	M00658-V-PU1_Q6
M00941-V-MEF2_Q6_01	2.13239967282312	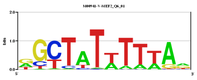 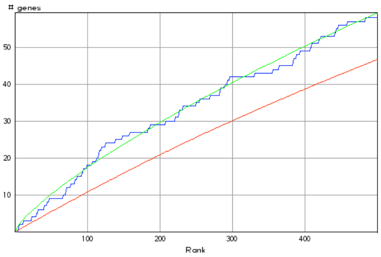	link	link	M00941-V-MEF2_Q6_01
M00253-V-CAP_01	2.12632336239156	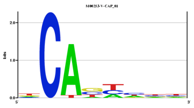 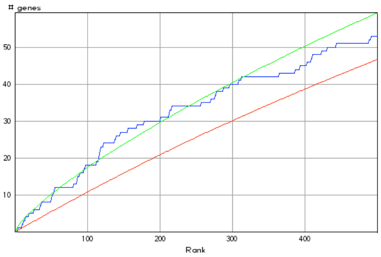	link	link	M00253-V-CAP_01
M00256-V-NRSF_01	2.11093766042492	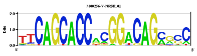 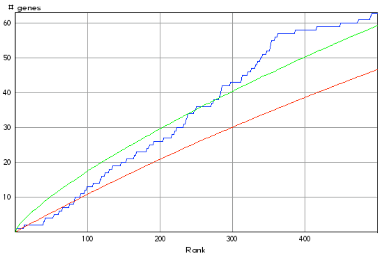	link	link	M00256-V-NRSF_01
MF0005	2.1088674130655	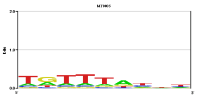 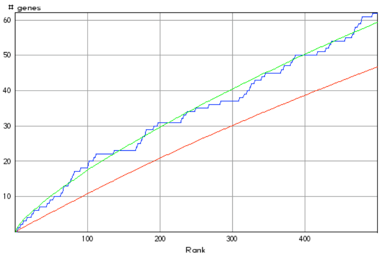	link	link	MF0005
sna	2.09340777369317	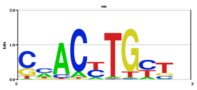 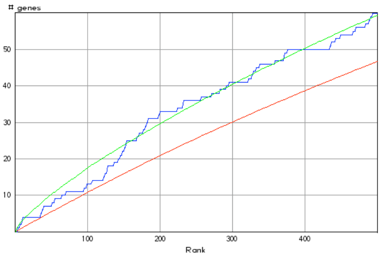	link	link	sna
CAACAACA	2.05977969726719	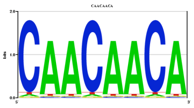 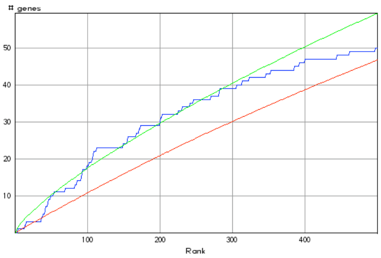	link	link	CAACAACA

exd	2.04971076692816	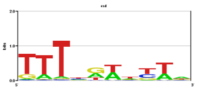	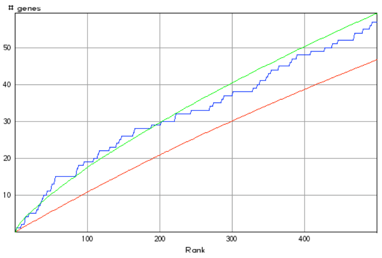	link	link	exd
M00644-V-LBP1_Q6	2.04030727324039	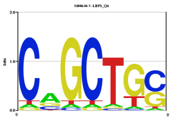	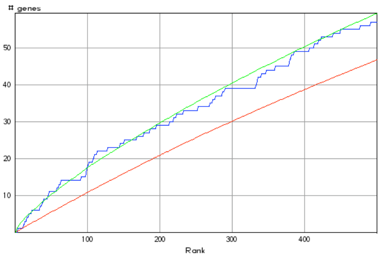	link	link	M00644-V-LBP1_Q6
M00461-I-OVO_01	2.02417547563448	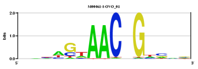	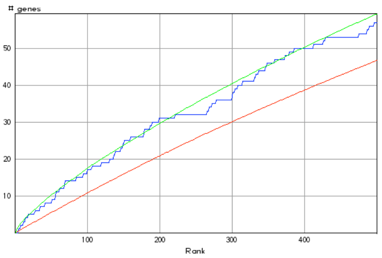	link	link	M00461-I-OVO_01
M00029-F-HSF_01	2.00469633002535	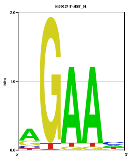	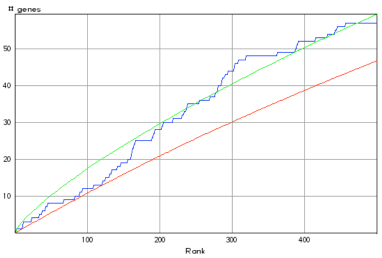	link	link	M00029-F-HSF_01
MA0027	1.9838459816197	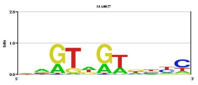	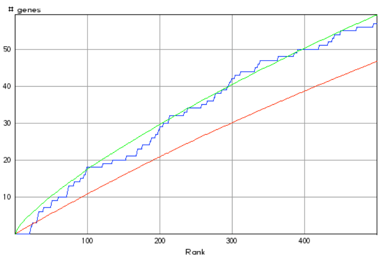	link	link	MA0027
M00345-P-GAMYB_01	1.98319398813313	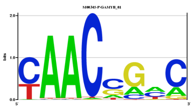	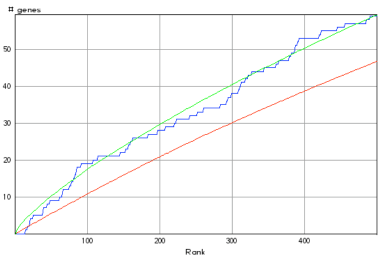	link	link	M00345-P-GAMYB_01
M00184-V-MYOD_Q6	1.97709751295457	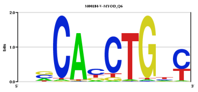	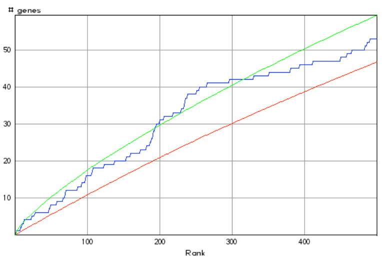	link	link	M00184-V-MYOD_Q6

M00913-V-MYB_Q5_01	1.97643207630332	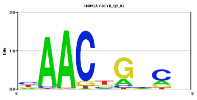	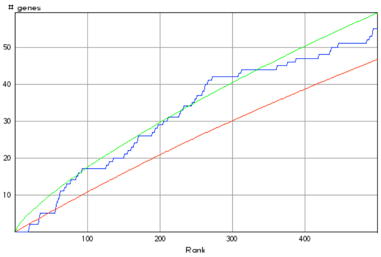	link	link	M00913-V-MYB_Q5_01
temp-ato_and_sens	1.96433994968123		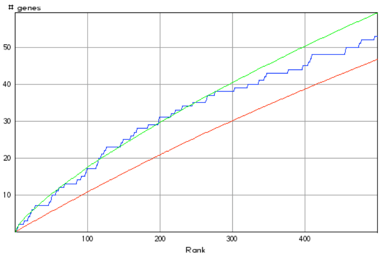	link	link	temp-ato_and_sens
