# Supplementary material for: Robust Target Gene Discovery through Transcriptome Perturbations and Genome-Wide Enhancer Predictions in Drosophila Uncovers a Regulatory Basis for Sensory Specification
Source: PLoS Biol. 2010 Jul 27;8(7):e1000435. doi: 10.1371/journal.pbio.1000435 (PMC2910651; doi:10.1371/journal.pbio.1000435)
Supplement: Table S8 — Primers for candidate Ato target enhancers. Positive Ato target enhancers are shaded in green. The boundaries of a CRM are determined automatically by Cluster-Buster, and the flanking sequence of the CRM was determined manually using the multiz alignments across 12 Drosophila species in the UCSC Genome Browser. Primers were designed in regions with low conservation, to amplify an enclosing genomic region with overall high sequence conservation, including the predicted CRM. For sens, E(spl), m4, and siz published reporter lines were used [32],[36],[37]. (0.07 MB PDF) [file pbio.1000435.s019.pdf]

Supplementary Table S8

Primers for candidate Ato target enhancers. Positive Ato target enhancers are shaded in green. The boundaries of a CRM are determined automatically by Cluster-Buster [22], and the flanking sequence of the CRM was determined manually using the multiz alignments across 12 *Drosophila* species in the UCSC Genome Browser. Primers were designed in regions with low conservation, to amplify an enclosing genomic region with overall high sequence conservation, including the predicted CRM. For *sens, E(spl), m4,* and *siz* published reporter lines were used [23,24,25].

gene	CG	forward primer	reverse primer
Mob2	CG11711	TCAGCTTGATAAAAATGATAAAGTGAT	TAAACGTTTTGCCACACAGC
sNPF	CG13968	AAACAAAAATACGAGCATGTGG	TATTCGGGATTTGGGTGTTG
ato	CG7508	CATCTCTCCTAGGCATCGC	GATCCTGGTCCCACGAAAC
nvy	CG3385	TCCTATTAGACGGCCCAGGT	TTGAAGTGCAATTCGATTACC
beat-IIIc	CG15138	TGCCCACAAAATACCAACCT	AATGGCTTCCTGCTCCTTCT
Fas2	CG3665	TGGCATCTGTTCTTTAATTTATTGTC	TTGACTCGATGGTTACCTATTTTT
CG30492	CG30492	AGGTACTAGGTTATGAGTGCTATTCCA	TGATTGTAGGTTCGAGGTTCG
CG1625	CG1625	GCGCATTGAGTTGCGTATC	TAGCTTAGAATTAAGCACTCATTTAGA
amon	CG6438	CGCAAGAACATCTCCAACAA	GAAAACGGAGTCCCAAATGA
spir	CG10076	GGTCTGTGATCGTCAATTTCTT	AGCTTCTGTGGAAACTGTGGA
Lim3	CG10699	CAAAGGGCTTGGTACTCGAC	ATGAAATGCCCAAATCCTTT
Dscam	CG17800	GTTGGTGTCTCTGCACTGGA	CAGCAAAAACCGAGGGTAAA
Pde8	CG5411	GGCCATTTGAAAATTGCTTG	CTGGCGGTCTCGTCTTTTT
CG2556	CG2556	AAAGACAGTCTATAAACAACGCATGT	AAAGAAGGAGTAAGCCAGCTTGT
sca	CG17579	GTAAAGCAGTCGTGCACTGG	GGGGAATCGGGTTTGAATTA
a	CG6741	TGCTTTGGTAGTTTGTAGTCTGC	TGATGAATGACTGATGACATTATTT
CG8965	CG8965	CGCTTTGGTTGTACCGAAAT	TTTAACGTTGGTAAAATAAACTTCTG
Src64B	CG7524	AACCAGTAGAACCAGTATGAGTATCAA	AAGGTGATTCGCCGTATTTG
Spn	CG16757	CCCGCTTCTCTTACTCATTCC	CATTTGGAGTAGCGGCATTT
Rapgap1	CG34374	TGCAAACTCGCTGTTTTTGA	GTGAGTCCTGCACGCATCC
phyl	CG10108	TTCCGAAAATATGTGGAAATACAA	GCAAAGAACTAAGGTCTCGGAAT
spdo	CG31020	GACCTCAAAAACCAGCTCCA	GTGCAGACCAAAAACCTTCC
neur	CG11988	GTTGCTATCGGGCTTATGGT	TTTCGTGTGCAAGTCATTGG
Traf4	CG3048	TGAGGAACTAGAGATTCATTTTATTCA	CCCATGTTCCCACATAAACA
nmo	CG7892	GCTGATGAAGCAGTGGACAA	CACCGAAAACAATGTCGAA
mam	CG8118	TGGGTCCCTGCTCTGAACTA	AACACATTGTGAATTGCAGGA
Mmp2	CG1794	GTGCCAATCTTTGCTCTTGC	GAGCAGAACAGCACAGGCTA
CG9801	CG9801	TTTCTGTGGATGCATTGAGG	TAAACAAAACGGCTGCTACG
SRPK	CG8174	GGGTTTTGCTTTGGATTTGA	CCTTTATTTCCGCCAGGTGT
salm	CG6464	AGAAAGCTGGGTGCAAAACACTCTTTCTA ACTGCAC	AAAAAGCAGGCTAAAATGAATTCTGTAC CGAAAAGT
DAAM	CG14622	AGCGAAAGAGTGGAAGAGCA	GATTCCCGATAACGGATTGA
Teh1	CG12806	TGGCCAATATCTGCCTACTTG	AAGACACAGGCATGTTTTAAGTT
CG31176	CG31176	TTCCAAAGCATCAAAATGGT	CCATTTTATGTGAGCCACTGA
CG6024	CG6024	TAGCCCTTTTGCCTTTGTGA	TGTCTGGTTGTACTCCACCAA
dap	CG1772	CCAATGCTTTGTGGGAGATT	AGACGTTTGCGCTCCTTCAT
sens	CG32120		
E(spl)	CG8365		
siz	CG32434		
m4	CG6099		
