# Supplementary material for: Robust Target Gene Discovery through Transcriptome Perturbations and Genome-Wide Enhancer Predictions in Drosophila Uncovers a Regulatory Basis for Sensory Specification
Source: PLoS Biol. 2010 Jul 27;8(7):e1000435. doi: 10.1371/journal.pbio.1000435 (PMC2910651; doi:10.1371/journal.pbio.1000435)
Supplement: Table S9 — Genomic location and size of candidate Atonal target enhancers. Positive Ato target enhancers are shaded in green. (0.07 MB PDF) [file pbio.1000435.s020.pdf]

Supplementary Table S9

Genomic location and size of candidate Atonal target enhancers. Positive Ato target enhancers are shaded in green.

gene	April 2004 location (dm2)	April 2006 location (dm3)	size
Mob2	chr3L:11530702-11531991	chr3L:11549855-11551144	1290
sNPF	chr2L:20026000-20027400	chr2L:20030071-20031471	1401
ato	chr3R:4099904-4100268	chr3R:4099904-4100268	365
nvy	chr2R:19787721-19788721	chr2R:20167644-20168644	1001
beat-IIIc	chr2L:17247550-17248500	chr2L:17251621-17252571	951
Fas2	chrX:4038337-4038876	chrX:4087133-4087672	540
CG30492	chr2R:3134600-3135200	chr2R:3510292-3510892	601
CG1625	chr2R:5338900-5339700	chr2R:5714592-5715392	801
amon	chr3R:22533529-22536836	chr3R:22533529-22536836	3308
spir	chr2L:20301835-20303494	chr2L:20314242-20315901	1660
Lim3	chr2L:19093033-19095443	chr2L:19097104-19099514	2411
Dscam	chr2R:2882600-2883700	chr2R:3258292-3259392	1101
Pde8	chr2R:19173800-19174900	chr2R:19553723-19554823	1101
CG2556	chrX:12332785-12333586	chrX:12392881-12393682	802
sca	chr2R:8300700-8302200	chr2R:8672298-8673798	1501
a	chr2R:17669503-17670148	chr2R:18049426-18050071	646
CG8965	chr2L:5881996-5882615	chr2L:5881996-5882615	620
Src64B	chr3L:4594004-4595499	chr3L:4613157-4614652	1496
Spn	chr3L:2536854-2537222	chr3L:2553322-2553690	369
Rapgap1	chr2L:7515915-7516716	chr2L:7515915-7516716	802
Phyl	chr2R:9945310-9946435	chr2R:10317952-10319077	1126
spdo	chr3R:26300453-26301460	chr3R:26300453-26301460	1008
neur	chr3R:4850273-4851081	chr3R:4850273-4851081	809
Traf4	chr2L:4374545-4376244	chr2L:4374545-4376244	1700
nmo	chr3L:8002747-8003500	chr3L:8021900-8022653	754
mam	chr2R:9520312-9520962	chr2R:9891910-9892560	651
Mmp2	chr2R:5143610-5144697	chr2R:5519302-5520389	1088
CG9801	chr3R:4566442-4566954	chr3R:4566442-4566954	513
SRPK	chr2R:10,896,089-10,896,737	chr2R:11268731-11269379	649
salm	chr2L:11,440,397-11,441,498	chr2L:11440397-11441498	1102
DAAM	chrX:1,161,797-1,162,714	chrX:1199009-1199926	918
Teh1	chr3R:5,688,430-5,689,248	chr3R:5688430-5689248	819
CG31176	chr3R:17,536,337-17,537,273	chr3R:17,536,337-17,537,273	937
CG6024	chr3L:11729931-11730489	chr3L:11749084-11749642	559
dap	chr2R:5,225,886-5,226,747	chr2R:5601578-5602439	862
sens	chr3L:13,372,404-13,373,050	chr3L:13391557-13392203	**647**
m4	chr3R:21,850,354-21,850,632	chr3R:21850354-21850632	279
E(spl)	chr3R:21,864,777-21,865,879	chr3R:21864777-21865879	1103
siz	chr3L:20,999,789-21,001,682	chr3L:21059048-21060941	1894
