# Supplementary material for: Robust Target Gene Discovery through Transcriptome Perturbations and Genome-Wide Enhancer Predictions in Drosophila Uncovers a Regulatory Basis for Sensory Specification
Source: PLoS Biol. 2010 Jul 27;8(7):e1000435. doi: 10.1371/journal.pbio.1000435 (PMC2910651; doi:10.1371/journal.pbio.1000435)
Supplement: Table S10 — Predicted cis -regulatory interactions in the transcriptional network underlying early retinal differentiation. Interactions in italics are drawn from the literature, while all other predictions result from cisTargetX analyses described in this study. This list of interactions is used directly as input for network mapping in the BioTapestry software (see Figure S11). (0.12 MB PDF) [file pbio.1000435.s021.pdf]

Supplementary Table S10

***Cis*-regulatory interactions in the transcriptional network underlying early retinal differentiation.** Interactions in *italics* are drawn from the literature.

TF	TG
sens	*Ro*
ro	*sens*
ato	*sens*
ey	*ato*
so	*ato*
ey	*Optix*
ey	*eya*
ey	*shf*
sens	*Rh5*
sens	*Rh6*
pros	*Rh5*
pros	*Rh6*
sens	*Rh3*
sens	*Rh4*
Otd	*Rh3*
Otd	*Rh5*
Otd	*Rh6*
ato	*Brd*
ato	*dap*
glass	*pros*
so	*pros*
ey	*so*
toy	*so*
lz	*argos*
lz	*klu*
eya	*dac*
so	*dac*
ey	*dac*
pnt	*Brd*
pnt	*dap*
Dfd	*Dfd*
toy	*ey*
ey	*eya*
dac	*eya*
pnt	*hh*
so	*so*
so	*ey*
so	*hh*
Su(H)	*HLHmgamma*
so	*lz*
glass	*lz*
pnt	*pros*
yan	*pros*
lz	*pros*
ey	*so*
z	*w*
sens	sn
sens	gpp
sens	vn
sens	sano
sens	CG14351
sens	CG15630
sens	Mmp2
sens	Eip78C
sens	jeb
sens	CG31176
sens	CG32169
sens	Eip74EF
sens	alan-shepard
sens	rn
sens	argos
sens	a
sens	CadN
sens	sr
sens	nmo
sens	mam
sens	Eip75B
sens	CG9095
sens	grn
sens	CG9924
ato	Abl
ato	CG13928
ato	CG15863
ato	CG1625
ato	CG17378
ato	CG17724
ato	CG2556
ato	CG30343
ato	CG30492
ato	CG31176
ato	CG31637
ato	CG31871
ato	CG32030
ato	CG32131
ato	CG32169
ato	CG32206
ato	CG32387
ato	CG32677
ato	CG33515
ato	CG6024
ato	CG6495
ato	CG6860
ato	siz
ato	spdo
ato	spir
ato	CG15097
ato	Ank2
ato	beat-IIIa
ato	HLHmdelta
ato	CG32150
ato	vn
ey	so
ey	Optix
ey	CG32521
ey	eya
ey	toy
ey	Fas2
ey	CG5888
ey	Tie
ey	osp
ey	CG30492
ey	mspo
ey	CG17816
ey	SK
ey	ey
ro	CG15824
ro	CG32726
ro	pip
ro	sens
ro	unc-13-4A
Su(H)	Abl
Su(H)	CG11498
Su(H)	CG14351
Su(H)	CG15097
Su(H)	CG2556
Su(H)	CG30492
Su(H)	CG31005
Su(H)	CG31176
Su(H)	CG32030
Su(H)	CG32150
Su(H)	CG5873
Su(H)	CG6026
Su(H)	CG6860
Su(H)	CG8965
Su(H)	DAT
Su(H)	Dl
Su(H)	Dscam
Su(H)	E(spl)

ey	*Rh1*	ato	CG8179	Su(H)	Eip75B
Deaf1	*Dfd*	ato	CG8965	Su(H)	Fas2
sens	caps	ato	CG9095	Su(H)	Fs
sens	sNPF	ato	CG9801	Su(H)	HLHm7
sens	bru-3	ato	CG9924	Su(H)	HLHmdelta
sens	beat-IIIc	ato	CadN	Su(H)	HLHmgamma
sens	Con	ato	DAAM	Su(H)	MYPT-75D
sens	cpx	ato	DmsR-1	Su(H)	Mob1
sens	unc-13-4A	ato	Dscam	Su(H)	Pde8
sens	dpr9	ato	E(spl)	Su(H)	Rapgap1
sens	phyl	ato	Eip75B	Su(H)	S
sens	CG13982	ato	Fas2	Su(H)	Teh1
sens	CG8910	ato	Lim3	Su(H)	Traf1
sens	CG14509	ato	MYPT-75D	Su(H)	argos
sens	CG31646	ato	Mmp2	Su(H)	ato
sens	sca	ato	Mob1	Su(H)	beat-IIIc
sens	Mob1	ato	Pde8	Su(H)	dap
sens	king-tubby	ato	Pka-R2	Su(H)	hts
sens	pgant2	ato	Rapgap1	Su(H)	king-tubby
sens	CG30492	ato	SRPK	Su(H)	klu
sens	oa2	ato	Spn	Su(H)	lola
sens	amon	ato	Src64B	Su(H)	m4
sens	Lim3	ato	Teh1	Su(H)	malpha
sens	dpr10	ato	Traf1	Su(H)	neur
sens	CG32131	ato	Victoria	Su(H)	nmo
sens	CG32726	ato	a	Su(H)	nvy
sens	CG17111	ato	alan-shepard	Su(H)	phyl
sens	Lgr3	ato	amon	Su(H)	qua
sens	Rh50	ato	beat-IIIc	Su(H)	retn
sens	sens	ato	betaTub60D	Su(H)	rho
sens	CG6142	ato	br	Su(H)	sNPF
sens	pip	ato	bru-3	Su(H)	sim
sens	CdGAPr	ato	cenG1A	Su(H)	siz
sens	CG12607	ato	cup	Su(H)	spir
sens	CG13033	ato	dap	Su(H)	ths
sens	GluRIIC	ato	dpr	Su(H)	tll
sens	Cyp49a1	ato	dpr10	Su(H)	vn
sens	lola	ato	dpr8	glass	lz
sens	itp	ato	dpr9	glass	kirre
sens	Trl	ato	f	glass	CG3777
sens	NK7.1	ato	hts	glass	CG6151
sens	Cirl	ato	king-tubby	glass	CG9924
sens	mbl	ato	kirre	glass	cenG1A
sens	klu	ato	lola	glass	ltd
sens	Dll	ato	mam	glass	beat-IIIc
sens	hth	ato	n-syb	glass	CG30497
sens	pyd	ato	nerfin-1	glass	Mmp2
sens	PhKgamma	ato	neur	glass	CG32169
sens	br	ato	nmo	glass	Lim1
sens	fz	ato	nvy	glass	CG3074
sens	CG17724	ato	phyl	glass	ct
sens	Taf4	ato	sNPF	glass	CG8776
sens	CG17838	ato	salm	glass	CG32521

sens	CG33275	ato	sano	glass	toc
sens	siz	ato	sca	glass	Prosap
sens	Fas2	ato	scrt	glass	Rapgap1
sens	W	ato	sens	glass	tmod
sens	simj	ato	seq	glass	sano
sens	Ace	ato	side	glass	kuz
