# Supplementary material for: Robust Target Gene Discovery through Transcriptome Perturbations and Genome-Wide Enhancer Predictions in Drosophila Uncovers a Regulatory Basis for Sensory Specification
Source: PLoS Biol. 2010 Jul 27;8(7):e1000435. doi: 10.1371/journal.pbio.1000435 (PMC2910651; doi:10.1371/journal.pbio.1000435)
Supplement: Table S11 — Over-represented motifs among 21 Ato target enhancers. The enhancer set comprises the 17 novel Ato targets from the Ato GOF and LOF analysis plus the rediscovered sens, ato, and dap enhancers, plus the previously known Brd enhancer. Over-represented motifs were determined by Clover [14]. The background sequence used for Clover, to select random sequence sets from, was the set of all 5-kb upstream and intronic regions. The number of randomizations was set to 10,000. The PWM collection used for Clover is the same as the basic collection of 1,981 PWMs used by the cisTargetX analyses, from Table S1A. (0.09 MB PDF) [file pbio.1000435.s022.pdf]

Supplementary Table S11

Over-represented motifs among 21 Ato target enhancers. The enhancer set comprises the 17 novel Ato targets from the Ato GOF and LOF analysis plus the re-discovered *sens*, *ato*, and *dap* enhancers, plus the previously known *Brd* enhancer. Over-represented motifs were determined by Clover [26]. The background sequence used for Clover, to select random sequence sets from, was the set of all 5kb upstream and intronic regions. The number of randomizations was set to 10000. The PWM collection used for Clover is the same as the basic collection of 1981 PWMs used by the cisTargetX analyses, from Table S1-A.

Motif	Clover Raw Score	P-value
AACAGCTG	23.9	0
RACASCTGY	21	0
atopwm3	20.8	0
atopwm7	19.7	0
MA0055	18.9	0
M00929-V-MYOD_Q6_01	18.2	0
nnnrwcakstgbn	16.4	0
AACAGGTGGCA	15.9	0
M00001-V-MYOD_01	15.4	0
M00804-V-E2A_Q2	15	0.001
M00712-V-MYOGENIN_Q6	14.6	0
M00693-V-E12_Q6	14.5	0
M00184-V-MYOD_Q6	14.3	0
CAGSTG-asense	13.9	0
PF0015	13.8	0
M00973-V-E2A_Q6	13.2	0
CAGCTGC	12.4	0.004
M00002-V-E47_01	12.3	0
AWCAGGTGK-atonal	12.3	0
RRCAGGTGB-escargot	11.4	0.001
M00927-V-AP4_Q6_01	11.2	0
SMATAAAAAA-hunchback	10.7	0.009
MA0050	10.6	0.007
MA0091	10.4	0
M00175-V-AP4_Q5	10.3	0.001
M00277-V-LMO2COM_01	9.82	0.003
PF0035	9.81	0.001
M00066-V-TAL1ALPHAE47_01	9.54	0
M00071-V-E47_02	9.48	0
ATCAGCTG	9.38	0
M00698-V-HEB_Q6	9.38	0.003
M00176-V-AP4_Q6	9.31	0.001
M00005-V-AP4_01	8.82	0.002
CAGGTGGC	8.24	0
ACAGGTG	7.43	0
M00993-V-TAL1_Q6	7.13	0.01
M00060-I-SN_01	7.04	0
M00065-V-TAL1BETAE47_01	6.09	0.001
CAGGTGG	5.75	0.002

M01034-V-EBOX_Q6_01	5.28	0.007
ACCTGTTG	5.12	0
PF0077	4.74	0.005
CAGGTG-snail	4.7	0.002
CCTGTTGC	4.56	0.009
PF0090	4.35	0.004
M00074-V-CETS1P54_02	4.27	0.002
MA0028	4.14	0.007
TIFDMEM0000079	4.01	0
Eip74EF	3.86	0.005
TGACCCA	3.68	0.005
M00414-V-AREB6_03	3.35	0.009
MA0026	3.34	0.008
M00983-V-MAF_Q6_01	3.13	0.009
ACACCTG	3.01	0.003
MA0069	2.78	0
M00097-V-PAX6_01	2.71	0.004
CAGGAGG	2.61	0.008
GATCCTC	2.46	0
MA0111	2.1	0.004
M00070-V-TAL1BETAITF2_01	1.96	0.006
GGGTCA-Hormone	1.92	0.005
MA0103	1.83	0.006
CAGGTAG	1.61	0.003
M00191-V-ER_Q6	1.17	0.01
CATGAAG	0.777	0.01
TIFDMEM0000089	0.0461	0.997
AGATCCT	-0.0543	0.007
p120	-0.937	0.995
CAATGCACTTCTGGGGCTTCCAC-glass	-1.4	0.008
sd	-1.48	0.999
M00515-V-PPARG_02	-2.03	0.007
MA0024	-2.05	0.992
M00289-V-HFH3_01	-2.5	0.995
M01030-F-RIM101_01	-2.52	0.999
M00454-V-MRF2_01	-2.98	0.999
ATGGCCG	-3.11	0.997
ACGNNAATTG	-3.3	0.995
TAATTGAC	-3.36	0.995
M00738-V-E2F4DP1_01	-3.74	0.993
GCCACGCCC	-3.79	0.995
M00810-V-SRF_Q4	-4.46	0.993
M00742-V-HFH4_01	-4.49	0.991
M00088-V-IK3_01	-4.61	0.992
M00463-V-POU3F2_01	-4.61	0.997
MA0052	-5.19	0.999
M00407-V-RSRFC4_Q2	-5.41	0.997
M00538-V-HTF_01	-5.58	1
M00026-V-RSRFC4_01	-6.16	0.999
M00231-V-MEF2_02	-6.16	1
PF0161	-6.88	0.992
M00232-V-MEF2_03	-7.39	1
PF0054	-7.39	0.996

PF0071	-8.04	0.999
PF0031	-9.15	0.993
