# Supplementary material for: Robust Target Gene Discovery through Transcriptome Perturbations and Genome-Wide Enhancer Predictions in Drosophila Uncovers a Regulatory Basis for Sensory Specification
Source: PLoS Biol. 2010 Jul 27;8(7):e1000435. doi: 10.1371/journal.pbio.1000435 (PMC2910651; doi:10.1371/journal.pbio.1000435)
Supplement: Text S1 — Overview of cis TargetX using dorsal (dl) as an example. (0.21 MB PDF) [file pbio.1000435.s023.pdf]

Text S1: Overview of cisTargetX using dorsal (dl) as an example

CISTARGETX consists of two steps, illustrated in the Figure below for a positive control TF, namely Dorsal, for which an informative position weight matrix (PWM) and many direct target genes are known [1,2,3]. In the first step (A-F), the Cluster-Buster [4] algorithm and a PWM is used to predict binding site clusters (BSC) in all 5kb upstream sequences and all introns for all *Dmel* genes, and in all the *net* alignments [5] of these regions to 10 other *Drosophila* genomes. The choice of 5kb upstream and introns as search space is based on a benchmark study we performed before [6]. The Cluster-Buster scores are converted to ranks in each species separately and then combined into a final ranking of all *Dmel* genes (F). We recently reported that this procedure to score multiple genomes is a powerful approach to use comparative genomics, without being dependent on strictly aligned binding sites [6]. By scoring each orthologous region separately, this method takes advantage of evolutionary conservation without being affected by alignment artifacts or binding site plasticity [7,8,9,10]. Note that for this example and for the applications in the retinal differentiation network we only perform homotypic scans (i.e,. one PWM is used as input of Cluster-Buster) because we explicitely search for target genes of a one particular TF. An extension of this framework to heterotypic scans is feasible [11].

In the second step of the CISTARGETX procedure (G-H), we plot the genomic ranks of a set of co-expressed genes in a cumulative recovery curve (H), as applied before on similar or related problems [6,11,12,13,14]. If the co-expressed gene set is significantly enriched for direct targets of the PWM, we expect the area under the recovery curve (blue curve in H) to be significantly larger than the area under the control curve. A theoretical control curve, for example calculated by the hypergeometric p-value at each rank cut-off, is not valid because longer loci have a higher likelihood of being selected purely by chance [14,15]. Therefore, we use an empirical distribution of control curves over nearly 2000 PWMs (Table S2). The areas under these curves, at x=410 (3% of the genome), are normally distributed. For 80 co-expressed genes downstream of dorsal (a mix of direct and indirect target genes) [3], the recovery curve for the dl PWM (MA0022) is indeed significantly better than the control curve (Z-score for AUC03 is 5.60), which means that this set is enriched for direct dl targets, compared to the rest of the genome *and* compared to the rest of the motif space or regulome. To determine the optimal cutoff we plot the average recovery curve and two standard deviations above this average, for the same set of input genes. At 3% of the genome, the numbers of recovered genes (y-axis) for all motifs, are also normally distributed. In our example, a cutoff at 220 genes yields a p-value of 1.97e-09 and predicts 13 direct target genes of dl, out of the 80 co-expressed input genes. Of these, 12 are true targets as reported by Zeitlinger et al [16], which yields a PPV of more than 90%, compared to a PPV less than 20% using motif predictions and comparative genomics alone.

Availability of cisTargetX

To make target gene prediction and regulatory network mapping possible for other biological processes, we have generated the genome-wide ranking for more than 2000 PWMs and created a web application that assesses whether an input gene set is statistically enriched for targets of any of these motifs. Because the scoring and ranking have been performed offline, users can upload their own candidate set and obtain the analysis results directly online, in about 15 minutes, without the need to install software or analyze genome sequences locally. Significantly enriched motifs can be selected, along with their predicted target genes, to obtain predicted enhancers and binding sites. The latter are visualized directly as custom track in the UCSC Genome Browser.

The application is available at http://med.kuleuven.be/cme-mg/lng/cisTargetX.

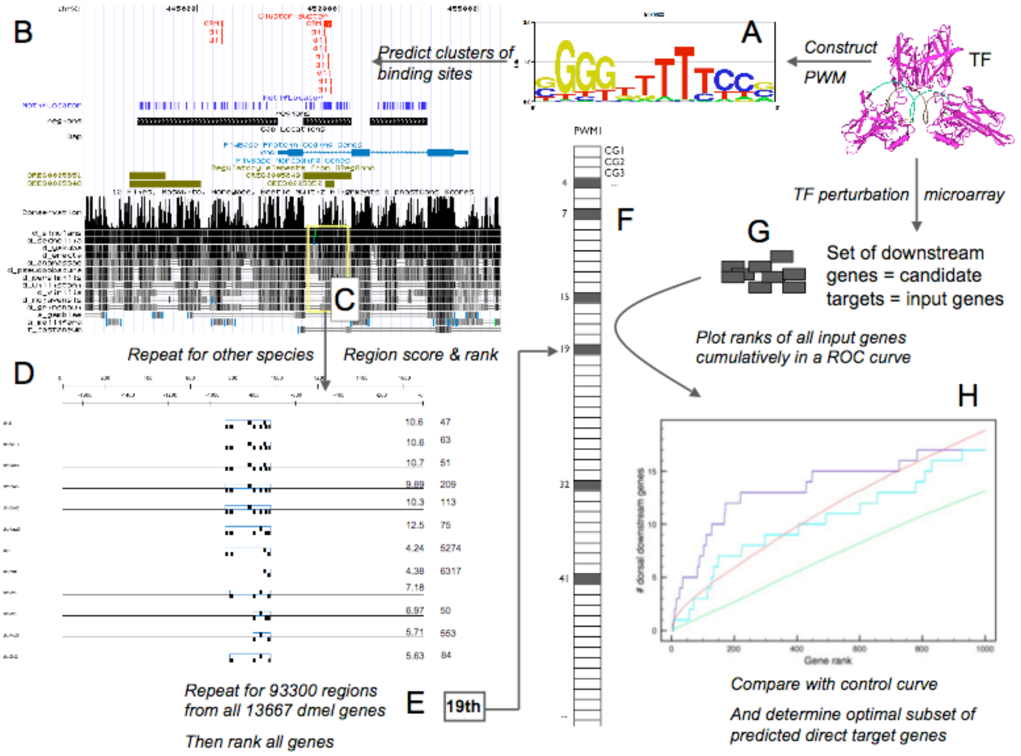

Overview of cisTargetX. (A). Position weight matrix for a TF under study, here dorsal. (B). Cluster-Buster scoring with PWM on all 5kb upstream and all intronic regions of Dmel (93330 regions in total). (C) *net* alignments are used to retrieve sequences for 11 other Drosophila species that are orthologous to the upstream and intronic Dmel sequences. (D) Example of the Cluster-Buster hit overlapping the dl target enhancer in a *vnd* intron. Not all binding site predictions are conserved. (E) To the right of the sequences are the Cluster-Buster scores (first column) and the ranks of the *vnd* gene in each genome independently. (F) The ranks for each species are integrated into one final gene ranking, using Dmel CG numbers as reference. (G) A set of candidate target genes, such as co-expressed genes after TF perturbation, is obtained, for example by microarray studies. (H) The genomic ranks of the candidate genes are plotted in a cumulative recovery curve (blue) and compared to the ranks obtained for the same genes, using genomic rankings by other PWMs.

References

1. Zeitlinger J, Zinzen RP, Stark A, Kellis M, Zhang H, et al. (2007) Whole-genome ChIP-chip analysis of Dorsal, Twist, and Snail suggests integration of diverse patterning processes in the Drosophila embryo. Genes Dev 21: 385-390.
2. Stathopoulos A, Levine M (2005) Genomic regulatory networks and animal development. Dev Cell 9: 449-462.
3. Stathopoulos A, Van Drenth M, Erives A, Markstein M, Levine M (2002) Whole-genome analysis of dorsal-ventral patterning in the Drosophila embryo. Cell 111: 687-701.
4. Frith CM, Li CM, Weng Z (2003) Cluster-Buster: Finding dense clusters of motifs in DNA sequences. Nucleic Acids Res 31: 3666-3668.
5. Kent W, Sugnet C, Furey T, Roskin K, Pringle T, et al. (2002) The human genome browser at UCSC. Genome Res 12: 996-1006.
6. Aerts S, van Helden J, Sand O, Hassan BA (2007) Fine-tuning enhancer models to predict transcriptional targets across multiple genomes. PLoS ONE 2: e1115.
7. Moses AM, Pollard DA, Nix DA, Iyer VN, Li XY, et al. (2006) Large-scale turnover of functional transcription factor binding sites in Drosophila. PLoS Comput Biol 2: e130.
8. Hare EE, Peterson BK, Iyer VN, Meier R, Eisen MB (2008) Sepsid even-skipped enhancers are functionally conserved in Drosophila despite lack of sequence conservation. PLoS Genet 4: e1000106.
9. Veitia RA (2008) One thousand and one ways of making functionally similar transcriptional enhancers. Bioessays 30: 1052-1057.
10. Ludwig MZ, Bergman C, Patel NH, Kreitman M (2000) Evidence for stabilizing selection in a eukaryotic enhancer element. Nature 403: 564-567.
11. Philippakis A, Busser B, Gisselbrecht S, He F, Estrada B, et al. (2006) Expression-guided in silico evaluation of candidate cis regulatory codes for Drosophila muscle founder cells. PLoS Comput Biol 2: e53.
12. Aerts S, Lambrechts D, Maity S, Van Loo P, Coessens B, et al. (2006) Gene prioritization through genomic data fusion. Nat Biotechnol 24: 537-544.
13. Van Loo P, Aerts S, Thienpont B, De Moor B, Moreau Y, et al. (2008) ModuleMiner - improved computational detection of cis-regulatory modules: are there different modes of gene regulation in embryonic development and adult tissues? Genome Biol 9: R66.
14. Warner JB, Philippakis AA, Jaeger SA, He FS, Lin J, et al. (2008) Systematic identification of mammalian regulatory motifs' target genes and functions. Nat Methods 5: 347-353.
15. Taher L, Ovcharenko I (2009) Variable Locus Length in the Human Genome Leads to Ascertainment Bias in Functional Inference For Noncoding Elements. Bioinformatics.
16. Zeitlinger J, Zinzen R, Stark A, Kellis M, Zhang H, et al. (2007) Whole-genome ChIP-chip analysis of Dorsal, Twist, and Snail suggests integration of diverse patterning processes in the Drosophila embryo. Genes Dev 21: 385-390.
