# Supplementary material for: Robust Target Gene Discovery through Transcriptome Perturbations and Genome-Wide Enhancer Predictions in Drosophila Uncovers a Regulatory Basis for Sensory Specification
Source: PLoS Biol. 2010 Jul 27;8(7):e1000435. doi: 10.1371/journal.pbio.1000435 (PMC2910651; doi:10.1371/journal.pbio.1000435)
Supplement: Text S2 — Comparison of cis TargetX with other methods. (0.33 MB PDF) [file pbio.1000435.s024.pdf]

Text S2: Comparison of cisTargetX with other methods

1. Motif discovery

cisTargetX is a meta-method composed of a series of sequential methodologies unified in a single platform and presented as a coherent online tool. The main goal of cisTargetX is to discover motifs in a set of co-expressed genes, define an optimal subset of genes as predicted targets of these motifs, and identify a set of predicted CRMs within these genes. Although, to our knowledge, no other methods with the same functionality exist, the methods that resemble cisTargetX in terms of input and output include the PWM-based motif discovery methods Clover [1], oPOSSUM [2], PASTAA [3], TOUCAN [4], the Genomatix suite [5], PAP [6], and PSCAN [7]. These methods identify significantly enriched motifs in a set of sequences (e.g., from co-expressed genes), compared to background sequences. We compared the results of Clover, PASTAA, and PSCAN with those of cisTargetX, on co-expressed gene sets for which we present cisTargetX analyses in the manuscript . Note that the default settings of these methods include limited sequence and motif, we therefore tested these methods either at default settings or with the same space as used for cisTargetX.

1.A. Default settings

- **PASTAA** is available online for 200bp promoters, but only for human and mouse data. We downloaded the source code and ran PASTAA locally, using all 200bp promoters of *Drosophila*. As PWM library we used the professional TRANSFAC library, similar to the online PASTAA version and the PASTAA publication.

- **Clover** was run locally on 1500 bp upstream sequences using JASPAR motifs, as was done in the Clover publication.

- **PSCAN** is available as an online web application and includes *Drosophila* data. Sequence search spaces available are: [-450,+50], [-500,0], [-200,50], [-950,+50], [-1000,0]. We chose the largest available sequence space, namely the [-1000,0] set together with the JASPAR PWM library.

Gene set	Motif	cisTargetX	Clover	PSCAN	PASTAA
Dl	dl (e.g., M00043-I-DL_01)	r=1	0<r<23 [c] (NF-kappaB)	r=4/125[b] (p value 0.056)	r=12 (NFKB_Q6)
Biniou	biniou (e.g., M00474-V-FOXO1_02)	r=1	Not found	Not found	r=397 (FOXO1_02)
Srp	srp (e.g., GATAAGC)	r=1	r=4 (GATA2)	Not found	r=2 (GATA_C)
posakony	SuH	r=1	Not found	r=4/125[b]	r=46
FC	Pnt (e.g,. M00935-V-	r=4	Not found	Not found	r=51 (ETS1_B)

	NFAT_Q4_01)				
PLE	Pnt (e.g., M00935-V-NFAT_Q4_01)	r=10	Not found	Not found	r=80 (ETS2_B)
Mef2	Mef2 (e.g,. MA0052)	r=3	0<r<10[c] (MEF2A)	Not found	r=19
	CFII	r=1		r=1	r=18
Ey	Ey	r=1	Not found	Not found	Not found[a]
Ato	Ebox (e.g., RACASCTGY)	r=1	Not found	Not found	r=12 (MYOD_Q6_01)
	SuH	r=2	Not found	R=7 [b] but not significant (p=0.078)	

[a] The ey motif from Ostrin et al. [8] is not present in TRANSFAC, but several PAX6 PWMs from vertebrates are present.
[b] PSCAN finds dl_1 and Su(H) motifs in most of the sets, indicating a specificity problem. For nearly all sets, the Trl motif is found as the best motif.
[c] The correct motif is part of all motifs that have the lowest p-value ($p<0.01$).

Overall, the methods perform worse than cisTargetX (Table 1). The main reasons likely are:

- The sequence space: all these methods were developed for proximal promoters while cisTargetX is developed for entire gene loci
- The PWM space: in cisTargetX we use 1981 motifs in the manuscript, and currently more than 2500 motifs are available in the online version. The tested methods have a disadvantage because their motif libraries are far from saturated.
- The comparison across species: the tested methods work on one species. We and others have previously shown that including multiple species improves motif scoring [9], and in cisTargetX we exploit that fact for motif discovery.
- cisTargetX applies a scoring step to predict *clusters* of a motif; therefore motifs do not have to be over-represented across the entire foreground sequence set. The other methods are developed for short sequences and hence rely on over-representation across the entire sequence set.
- The robustness of ROC curves: cisTargetX uses ROC curves, a statistical technique that balances sensitivity and specificity.
- cisTargetX has a number of additional features such as the selection of the optimal subset of target genes and the annotation of CRMs and binding sites in a genome browser. Because these features are not available in the other methods, we limited the benchmark to the discovery of the correct motif.

1.B. Adjusted sequence and motif space

To compensate for the differences in **sequence space** and **PWM space**, we ran Clover and PASTAA using the same sequence space used in this study (5kb upstream sequences and introns). Note that on such large sequence files, Clover is not efficient, and takes for example 54 hours for the dorsal case (80 co-expressed genes) on a standard 32bit linux server. This is mainly due to the selection of a set of random sequence sets to estimate a p-value, by default set to 1000 sets. For larger sequence files this is not feasible, and we were obliged to set r=100 (thus limiting the significance level to 0.01). However, this still required multiple days to run. The main problem is that these methods have not been optimized for large sequence spaces, and the results are below cisTargetX performances (see Table below). In addition to the computational disadvantage of Clover compared to cisTargetX (days compared to ~10 minutes per set), Clover returns too many significant motifs, all with p-value <0.01 without further prioritization. Hence, if the transcription factor or motif is not known *a priori*, it is not feasible to select the true or most likely motif involved. cisTargetX avoids this problem through motif ranking based on AUC-values and z-scores.

In contrast to Clover, our attempts to include all matrices failed For PASTAA. We therefore maintained the complete TRANSFAC pro collection 10.4 as above, which is still significantly larger than the online available TRANSFAC public version or JASPAR).

Gene set	Motif	cisTargetX	Clover2	PASTAA2
Dl	dl (e.g., M00043-I-DL_01)	r=1	0<r<56*	r=39
Biniou	biniou (e.g., M00474-V-FOXO1_02)	r=1	0<r<169*	100 (FOXO1_01)
Srp	srp (e.g., GATAAGC)	r=1	0<r<93*	15 (GATA_C)
posakony	SuH	r=1	Not found	114 (SUH_1)
FC	Pnt (e.g,. M00935-V-NFAT_Q4_01)	r=4	Not found	59 (ETS2_B)
PLE	Pnt (e.g., M00935-V-NFAT_Q4_01)	r=10	Not found	104 (ETS1_B)
Mef2	Mef2 (e.g,. MA0052)	r=3	0<r<112*	r=3 (MEF2_04)
	CFII	r=1	0<r<112*	725
Ey	Ey	r=1	Not found	123 (PAX6_01)
Ato	Ebox (e.g., RACASCTGY)	r=1	Not found	r=71 (TAL1BETAE47_01)
	SuH	r=2	Not found	r=35 (SUH_01)

The table below lists the top 5 motifs for each method. For Clover, more than 5 motifs have p-value<0.01; the five with the highest raw score are given.

Gene set	cisTargetX	Clover2	PASTAA2
Dl	**M00043-I-DL_01** 6.04<br>**MA0101** 5.84<br>**MA0022** 5.60<br>**M00053-V-CREL_01** 5.27<br>**GGGGAMWWCCM-schnurri** 5.10	PF0123 0<br>CAAGTGCA 0<br>tin 0<br>TYAAGTGS-ventral 0<br>PF0088 0	ROAZ_01 1.8638e-53<br>P53_01 3.6100e-49<br>CF1_02 1.2268e-41<br>DEAF1_01<br>6.7796e-30<br>ABF_Q2 9.2950e-27
Biniou	**M00474-V-FOXO1_02** 3.41<br>M00290-V-FREAC2_01 3.40<br>MA0109 3.22<br>TIFDMEM0000112 3.02<br>TTATS-mitochondrial 2.86	Top2 0<br>M00094-I-BRCZ4_01 0<br>CACCCAC 0<br>ACAACAAC 0<br>M00809-V-FOX_Q2 0	PPARG_01<br>1.0090e-15<br>TCF_1 8.0159e-15<br>GCNF_01 4.2387e-13<br>HEN1_02 7.5775e-07<br>PLZF_02 4.8210e-06
Srp	**GATAAGC** 6.01<br>**YGATAAGC** 5.94<br>VRGKTYAWTGAMMYY-Ecdysone 4.49<br>M00487-I-MTTFA_01 3.80<br>M00375-P-TGA1B_Q2 3.66	MA0122 0<br>ara 0<br>M00240-V-NKX25_01 0<br>MA0099-cFOS 0<br>bin 0	HNF4_DR1_Q3<br>3.7556e-06<br>BZIP911_02<br>5.1235e-06<br>HNF4_01_B<br>1.1464e-05<br>DR1_Q3 3.8673e-05<br>AHR_01 5.2245e-05
posakony	**M00234-I-SUH_01** 5.58<br>**M01112-V-RBPJK_01** 5.36<br>**CGTGNGAA** 5.13<br>**M00184-V-MYOD_Q6** 4.18<br>**M01111-V-RBPJK_Q4** 4.05	AAATCAAT 0<br>bin 0<br>MA0122 0<br>M00240-V-NKX25_01 0<br>RTAAATA-biniou 0	PAX1_B 6.3950e-04<br>PCF2_01 7.5147e-04<br>MAZR_01 8.5794e-04<br>NRSF_01 1.6998e-03<br>GCM_01 1.8728e-03
FC	M01003-V-HELIOSA_01 3.77<br>CAGGTAG 3.40<br>PF0076 3.36<br>**M00935-V-NFAT_Q4_01** 3.21<br>M00028-I-HSF_01 3.13	M00130-V-FOXD3_01 0<br>MA0041-HFH2 0<br>M00094-I-BRCZ4_01 0<br>MA0042-HFH3 0<br>exd 0	TCF_1 7.2524e-14<br>PPARG_01<br>1.4012e-09<br>HEN1_02 1.0629e-08<br>GCNF_01 6.3133e-08<br>ADF1_Q6_01<br>1.4307e-07
PLE	**M00233-V-MEF2_04** 4.40<br>M00495-V-BACH1_01 4.03<br>**M01103-I-TWI_Q6** 3.98<br>M00499-V-STAT5A_04 3.76<br>M00003-V-VMYB_01 3.70	Top2 0<br>M00094-I-BRCZ4_01 0<br>ACATATG 0<br>TCCTTGC 0<br>M00129-V-HFH1_01 0	HEN1_01 2.7180e-06<br>NRSF_01 3.9155e-05<br>MAF_Q6_01<br>1.7793e-04<br>SEF1_C 3.6733e-04<br>**MEF2_03** 4.1954e-04
Mef2	**M00012-I-CF2II_01** 4.96<br>MA0015 4.77<br>TIFDMEM0000034 4.74<br>**RTATATRTRB-Chorion** 4.63<br>**SelexConsensus_Cf2** 4.62	**M00013-I-CF2II_02** 0<br>**RTATATRTRB-Chorion** 0<br>**M00012-I-CF2II_01** 0<br>MA0015 0<br>AGAGAGAG 0	PPARG_01<br>8.9309e-08<br>GCNF_01 2.8616e-06<br>**MEF2_04** 1.0725e-05<br>CREBP1CJUN_01<br>7.0266e-05<br>T3R_01 1.3969e-04
Ey	**ey** 3.53<br>M00442-P-ABF_Q2 3.08<br>TIFDMEM0000033 3.04<br>M00399-P-ABF1_01 3.03<br>M00198-F-GAL4_C 2.98	AGAGAGAG 0<br>M00494-V-STAT6_01 0<br>M00723-I-GAGAFACTOR_Q6 0<br>Trl 0<br>M00526-V-GCNF_01 0	PPARG_01<br>7.6099e-12<br>TCF_1 1.0746e-11<br>GCNF_01 1.7578e-07<br>PLZF_02 9.7053e-07<br>SRF_C 3.4162e-06
Ato	**RACASCTGY** 3.86<br>**M00184-V-MYOD_Q6** 3.74<br>**M00693-V-E12_Q6** 3.63<br>**M00001-V-MYOD_01** 3.37<br>**M00973-V-E2A_Q6** 3.27	M00972-V-IRF_Q6_01 0<br>CACCCAC 0<br>M00935-V-NFAT_Q4_01 0<br>PF0091 0<br>PF0055 0	ROAZ_01 5.3310e-37<br>P53_01 1.5523e-24<br>CF1_02 5.3930e-22<br>ABF_Q2 1.0215e-18<br>DEAF1_01<br>1.3693e-17

In conclusion, when the search space and motif space are extended for Clover and PASTAA, these methods are able to detect a significant enrichment of the correct motif for several of the co-expressed gene sets, but overall, too many significant motifs are returned and the correct motif is not ranked in the top 5 motifs, as illustrated in the tables above.

2. Benchmarking cisTargetX components

An alternative benchmark is to compare the different components of the cisTargetX meta-method (Table below) to other methods with similar functions.

Steps of the cisTargetX method

Step	Description	We use
A	Scoring the whole genome with a library of PWMs	Cluster-Buster
B	Using comparative genomics to reward conserved CRMs	Integrated cluster-buster scores using order statistics
C	Testing for a correlation between a set of co-expressed genes and high-ranking CRMs/genes obtained from A+B for a particular motif	ROC curves
D	Selecting the best motif for a set of co-expressed genes	Z-scores, comparing AUC values of ROC curves for 2000 motifs
E	Selecting the best gene subset for a motif selected under D	Z-scores, comparing number of recovered genes with expected number from 2000 motifs

We have extensively benchmarked steps A and B for 34 *Drosophila* transcription factors in previous work [9]. Based on this benchmark analysis we selected particular implementations for A (Cluster-Buster) and B (CRM preservation rather than motif alignment). Recently, other methods with similar functions to step A, notably **SWAN** (also called MotifScan.v6), appeared. We have now compared cisTargetX using Cluster-Buster with cisTargetX using SWAN, for two cases, namely Dorsal and Atonal. For Atonal we find similar performances while for dorsal we find better performances using Cluster-Buster (see Figures below). We therefore developed SWAN-based genome-wide rankings to be used in cisTargetX and added it to the available online toolThe disadvantage of the current SWAN version is that it does not return motif locations, so that the final step of cisTargetX, with visualization of the CRM and motif predictions as genome browser tracks is still only feasible using Cluster-Buster.

In conclusion, we cannot compare cisTargetX directly to methods like SWAN, but since cisTargetX is a meta-method, it uses advanced motif-scoring methods like SWAN or Cluster-Buster as one of its components. The analyses performed by cisTargetX cannot be performed by methods like SWAN alone but require them to be integrated into cisTargetX.

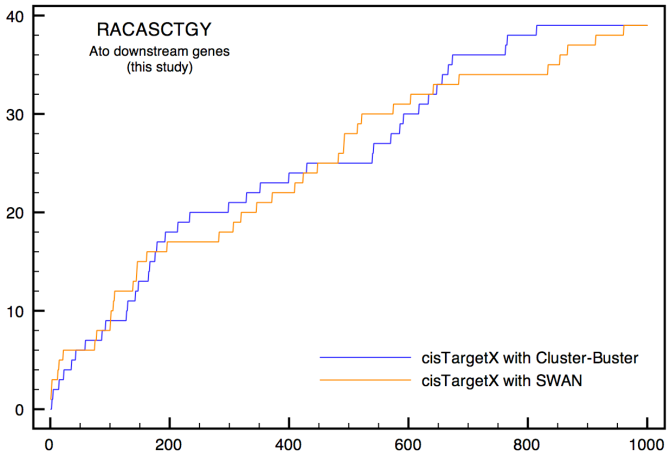

Comparison of cisTargetX-ClusterBuster with cisTargetX-SWAN, for a set of Atonal-downstream genes (204 genes) and the RACASCTGY motif.

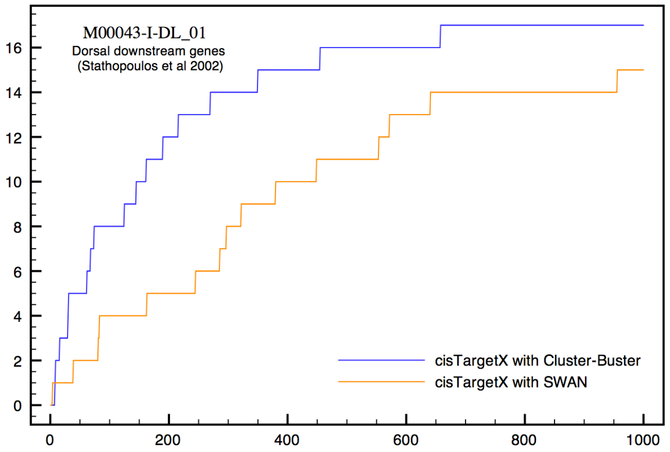

Comparison of cisTargetX-ClusterBuster with cisTargetX-SWAN, for a set of Dorsal-downstream genes (80 genes) and the dorsal motif from TRANSFAC.

3. Further notes on method comparisons

3.A. Comparison with ab initio motif discovery methods

Given that motif discovery is a central aim in cisTargetX, we compared cisTargetX with methods that can discover significantly over-represented motifs given a set of co-expressed genes (see above). cisTargetX uses the entire gene loci, including 5kb upstream sequence and all introns whereas traditional methods such as *ab initio* motif discovery methods on single genomes (e.g., MEME, MotifSampler) or multiple genomes (e.g., PhyloGibbs, PhyloCon) use only proximal promoter sequences . Such methods have been shown to be very inefficient on *Drosophila* sequences [10] and are not applicable to sequences larger than a few kilobases.

3.B. Comparison with methods that use enhancer training sets

Importantly, and contrary to cisTargetX, other methods for genome-wide discovery of CRMs rely on a set of similar – usually short - *regulatory regions* as input to train a

model or to discover motifs or motif combinations enriched in these regions. After training, these methods can usually be used to predict similar regulatory regions in the genome. For example the methods published recently in [11,12,13] start from a set of known regulatory regions. In our study we search for Atonal targets *de novo*, without using previously known enhancers, since only three enhancers were known. Also, when we tested cisTargetX on several validation sets (Table 1), we similarly started from sets of co-expressed genes, not from known enhancer sequences. Therefore, we cannot compare cisTargetX to methods that start from a set of regulatory sequences since they have a different purpose.

3.C. Comparison with other meta-methods
CisTargetX consists of multiple steps and can be considered as a *meta-method*. To our knowledge, cisTargetX is unique and no similar methods exist for *Drosophila*. Previously, we and others have proposed meta-methods for **vertebrate genomes** (Van Loo et al. Apr 2008 [14] and Warner et al. Apr 2008 [15]) based on similar ideas although these methods focus primarily on heterotypic enhancer models. They aim to discover the optimal combination of motifs across a set of co-expressed genes, while cisTargetX aims primarily at discovering individual transcription factors linked to their target genes. The main differences with the Warner et al. method are: (1) the species they are applied to; (2) the availability of a web-based tool and its computational efficiency allowing a gene set to be analyzed in minutes by a biologist; (3) the visualization of ROC curves for each motif; and (4) the selection of the optimal subset of predicted direct target genes and genomic binding sites for a motif, allowing to draw connections in a gene regulatory network. An additional impediment to porting ModuleMiner for whole-genome scoring, is that ModuleMiner is developed for genomes with conserved non-coding sequences (CNS) ('islands') – and such CNSs cannot be used in Drosophila with the currently available genomic sequences [16].

Conclusion

Only few methods exist that start from co-expressed genes, work on sequences larger than proximal promoters, and use PWM libraries. We have compared cisTargetX with Clover, PSCAN, and PASTAA and show that cisTargetX outperforms other methods for motif discovery. We have furthermore compared several components of cisTargetX with other components and present the most optimal combination of tools. Overall, cisTargetX is unique as a meta-method, combining motif discovery with accurate target gene prediction through a user-friendly web interface.

References

1. Frith MC, Fu Y, Yu L, Chen JF, Hansen U, et al. (2004) Detection of functional DNA motifs via statistical over-representation. Nucleic Acids Res 32: 1372-1381.
2. Ho Sui SJ, Fulton DL, Arenillas DJ, Kwon AT, Wasserman WW (2007) oPOSSUM: integrated tools for analysis of regulatory motif over-representation. Nucleic Acids Res 35: W245-252.
3. Roider HG, Manke T, O'Keeffe S, Vingron M, Haas SA (2009) PASTAA: identifying transcription factors associated with sets of co-regulated genes. Bioinformatics 25: 435-442.
4. Aerts S, Thijs G, Coessens B, Staes M, Moreau Y, et al. (2003) Toucan: deciphering the cis-regulatory logic of coregulated genes. Nucleic Acids Res 31: 1753-1764.
5. Cartharius K, Frech K, Grote K, Klocke B, Haltmeier M, et al. (2005) MatInspector and beyond: promoter analysis based on transcription factor binding sites. Bioinformatics 21: 2933-2942.
6. Chang LW, Fontaine BR, Stormo GD, Nagarajan R (2007) PAP: a comprehensive workbench for mammalian transcriptional regulatory sequence analysis. Nucleic Acids Res 35: W238-244.
7. Zambelli F, Pesole G, Pavesi G (2009) Pscan: finding over-represented transcription factor binding site motifs in sequences from co-regulated or co-expressed genes. Nucleic Acids Res 37: W247-252.
8. Ostrin JE, Li Y, Hoffman K, Liu J, Wang K, et al. (2006) Genome-wide identification of direct targets of the Drosophila retinal determination protein Eyeless. Genome Res 16: 466-476.
9. Aerts S, van Helden J, Sand O, Hassan BA (2007) Fine-tuning enhancer models to predict transcriptional targets across multiple genomes. PLoS ONE 2: e1115.
10. Tompa M, Li N, Bailey T, Church G, De Moor B, et al. (2005) Assessing computational tools for the discovery of transcription factor binding sites. Nat Biotechnol 23: 137-144.
11. Sinha S, Liang Y, Siggia E (2006) Stubb: a program for discovery and analysis of cis-regulatory modules. Nucleic Acids Res 34: W555-559.
12. Zinzen RP, Girardot C, Gagneur J, Braun M, Furlong EE (2009) Combinatorial binding predicts spatio-temporal cis-regulatory activity. Nature 462: 65-70.
13. Narlikar L, Sakabe NJ, Blanski AA, Arimura FE, Westlund JM, et al. Genome-wide discovery of human heart enhancers. Genome Res 20: 381-392.
14. Van Loo P, Aerts S, Thienpont B, De Moor B, Moreau Y, et al. (2008) ModuleMiner - improved computational detection of cis-regulatory modules: are there different modes of gene regulation in embryonic development and adult tissues? Genome Biol 9: R66.
15. Warner JB, Philippakis AA, Jaeger SA, He FS, Lin J, et al. (2008) Systematic identification of mammalian regulatory motifs' target genes and functions. Nat Methods 5: 347-353.
16. Peterson BK, Hare EE, Iyer VN, Storage S, Conner L, et al. (2009) Big genomes facilitate the comparative identification of regulatory elements. PLoS One 4: e4688.
